# Glutathione Peroxidase-Like Activity of Functionalized Tellurides: Insights into the Oxidation Mechanism Through Activation Strain Analysis

**DOI:** 10.1021/acs.inorgchem.5c00581

**Published:** 2025-05-09

**Authors:** Alessandro Rubbi, Damiano Tanini, Antonella Capperucci, Laura Orian

**Affiliations:** † Dipartimento di Scienze Chimiche, 9308 Università degli Studi di Padova, Via Marzolo 1, 35131 Padova, Italy; ‡ Dipartimento di Chimica “Ugo Schiff”, Università di Firenze, Via della Lastruccia 3-13, 50019 Sesto Fiorentino, Italy

## Abstract

The recent synthesis of a series of diorganotellurides as glutathione peroxidase mimics has prompted our in silico investigation on their oxidation mechanism by H_2_O_2_ at the ZORA-M06/TZ2P-ae//ZORA-OLYP/TZ2P level of theory. The role of the chalcogen (S and Se vs Te) on the energetics of the reactions has been elucidated within the framework of density functional theory and activation strain analysis. It emerges that the nature of the β-substituent plays a role in the catalytic activity that is found also when tellurium is replaced by its lighter siblings (S or Se). Our results provide general and useful insight for the development of small chalcogen-based organic molecules for the catalytic activation of H_2_O_2_.

## Graphical abstract

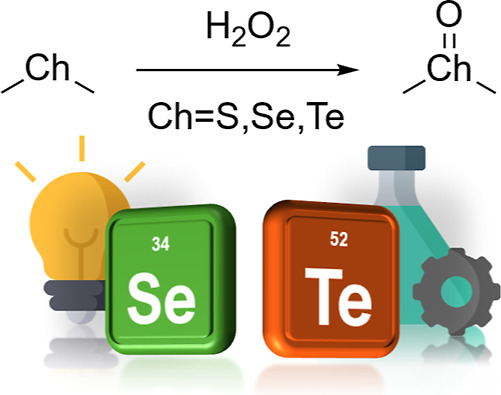

## 1 Introduction

Glutathione peroxidases (GPxs) are a family of sulfur- and selenium-based enzymes that are found in all living organisms.^1^ Among other biological roles, one of their key functions is assisting the homeostatic control of oxidative stress, especially by preventing the accumulation of hydroperoxides.^2–4^ These highly reactive species are harmful byproducts of the cellular metabolism that, when left unchecked, might lead over time to degenerative pathologies.^5^ Organoselenium compounds have been studied for a long time in virtue of their antioxidant capabilities, as they represent prime candidates in the search for small molecular equivalents of GPx,^3,6–9^ the most notable example among them being ebselen.^10,11^ Similarly, organotellurides have also attracted a lot of interest, since they share a nature akin to that of selenides but are often more reactive compared to their Se-based counterparts.^12–23^ Although knowledge on tellurium biochemistry is less extensive than that of the lighter chalcogens, on many occasions Te-containing organic molecules have been shown to display low toxicity, as well as pleasing antioxidant, chemopreventive and anticancer properties.^13,24–33^

Furthermore, organotellurium derivatives have emerged as interesting compounds due to their peculiar redox properties. Indeed, structurally diverse small-size tellurated organic molecules have been reported as efficient catalysts for the reduction of nitrogen compounds, as for example peroxynitrites or hydroperoxydes.^20^ These derivatives behave as dangerous biological oxidants, able to induce DNA damage or to initiate lipid peroxidation in biomembranes. Among the variety of organotellurium derivatives with biological activity, as the thiol-peroxidase-like properties, tellurides, ditellurides and Te-heterocycles are known to act as mimics of glutathione peroxidase.^20^

Alkyl-, aryl- and alkyl-aryl-disubstituted tellurides are classes of organic compounds of tellurium that catalyze the reduction of peroxides by thiols in aqueous or organic solvent.^34^ Detty and co-workers studied the kinetics of the Te^(II)^/Te^(IV)^ redox cycle in methanol in depth, drawing the conclusion that the reaction of diorganotellurides with H_2_O_2_ and thiols (RSH) follows the generalized GPx-like mechanism described in Scheme 1.^35^ The catalyst is initially oxidized from Te^(II)^ to Te^(IV)^ state, forming a telluroxide, and H_2_O_2_ is reduced (a). In the presence of water, the telluroxide reacts rapidly to yield its corresponding hydrated form, i.e., a dihydroxy tellurane (b). However, when the solvent is not aqueous, multiple Te^(IV)^ species are likely to be present at the same time in solution (b,c). Due to the poor solubility of organochalcogenides and the spontaneous oxidation of thiols in water, methanol (or CD_3_OD) has often been the solvent of choice for kinetic studies.^12,36^ Telluroxides are involved in several reversible reactions with MeOH and thiols, leading to an interchange of hydroxide, methoxide and thiolate ligands at the chalcogen center (b,c). These equilibria are assumed to occur much faster than the initial oxidation step.^23,35^ In the presence of thiols, the catalytic cycle is completed by a reductive elimination step, which regenerates the telluride catalyst and yields the oxidized sulfur compounds (d). Depending on the experimental conditions, the formation of the disulfide product occurs either directly, via a nucleophilic attack of RSH to the thiolate ligand, or via thiol-independent pathways, which involve the formation of thiotelluronium (R_2_Te^+^SR) or sulfenic ester (RSOMe) intermediates. In both cases, the actual elimination mechanism has almost no effect on the turnover of the catalyst and the initial Te^(II)^ to Te^(IV)^ oxidation is the rate-limiting step of the whole cycle.^35^

**Scheme 1:**
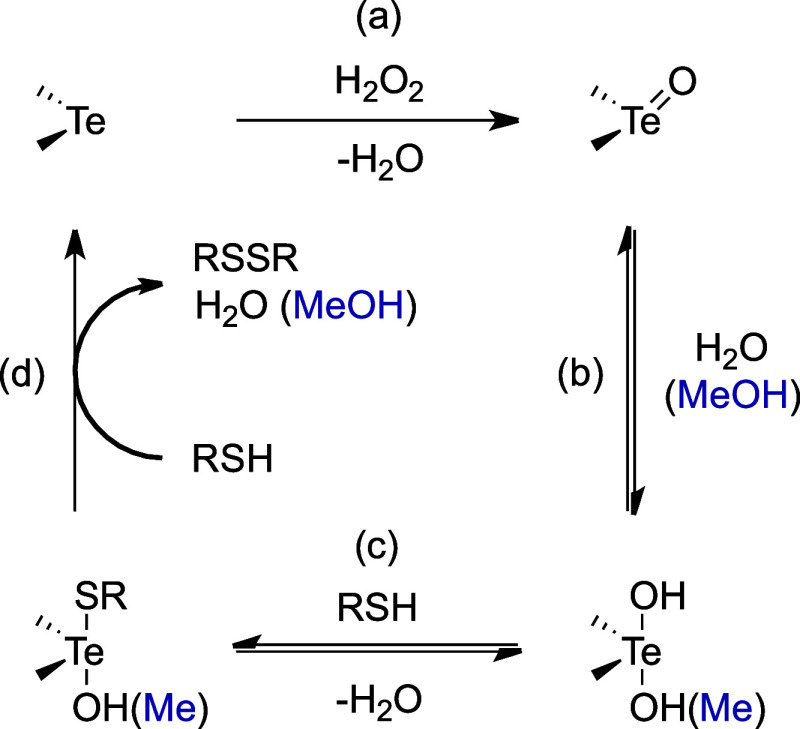
Catalytic Activity of Diorganotellurides^a^

a Proposed catalytic cycle of H_2_O_2_ reduction by diorganotellurides in aqueous solution and in MeOH. Adapted from Detty et al.^35^ Copyright 2003 American Chemical Society.

A few years ago, Tanini et al. synthesized and evaluated a series of functionalized diorganotellurides as catalysts for the thiol peroxidase-like reduction of H_2_O_2_.^37,38^ Differently from selenides, whose GPx mimic action^8,39^ and capacity of activating H_2_O_2_ in organic catalysis^40,41^ have been largely explored, mechanistic studies on organotellurides are scarce. Many of the compounds reported by Tanini et al. displayed a remarkable activity, although the nature of the substituent at β-position relative to the Te-center proved to have a significant influence on reaction rates. In essence, it was found that the introduction of a tosyl (Ts) group on the β-amino function of a phenyltelluro-amine catalyst led to a substantial decrease in activity. A possible explanation for the GPx-activity of the alkyl-aryl-tellurides bearing heteroatoms on the β-position might be the formation of intramolecular chalcogen bonding interactions (ChB), involving Te and the heteroatom at C-2 position, that could prevent the telluride oxidation or slow the thiol addition.^37,38^ This hypothesis was supported when phenyltellurides without a Ts-group displaying a significant catalytic activity as GPx mimics were synthesized and studied.^38^ In a similar manner, a β-disulfide phenyltelluro compound was less active than its β-allyl sulfide analog. The reason behind the effect of different substituting groups has remained unclarified so far, and a further investigation is keen sought-after.

Recently, the catalytic behavior of two heterocycles (tellural and tellenol) was reported, together with a comparative study of the intramolecular Te···X (X = H, N) interactions of these GPx mimics using a density functional theory (DFT) approach.^23^ A previous work on a selenated heterocycle as a GPx mimic, based on DFT calculations in the presence of microsolvation, was described, with methanethiol and thiophenol as nucleophiles.^21^

With the more general intent of understanding fundamental aspects of the reactivity of diorganochalcogenides, we have set up a Kohn–Sham DFT protocol and studied the oxidation mechanism of model functionalized tellurides (Figure 1) by H_2_O_2_. Sulfur and selenium analogs have been included in our analysis, to provide a broader picture of reactivity trends in the chalcogens’ group. Reaction barriers have been rationalized through a combined activation strain analysis (ASA) and energy decomposition analysis (EDA) approach.^42^ When deemed relevant, orbital interaction terms have been further decomposed according to the natural orbitals for chemical valence (NOCV) scheme.^43^

**Figure 1:**
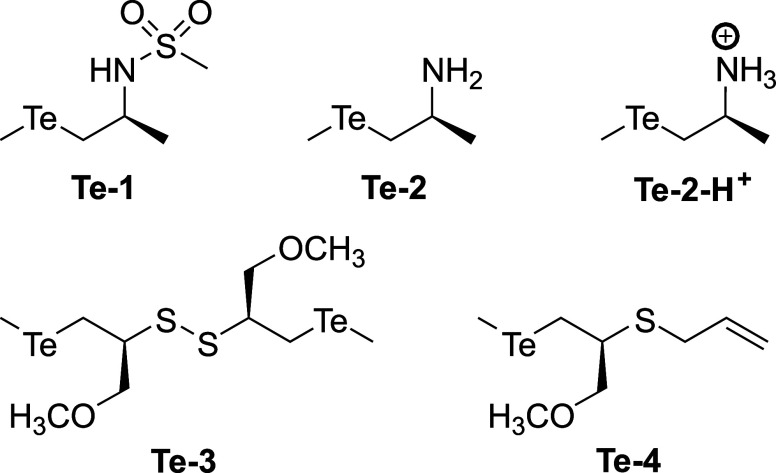
Diorganotellurides studied in this work.

## 2 Computational Details

All DFT calculations have been performed with the Amsterdam density functional (ADF) (v. 2019.307) and the Amsterdam modeling suite (AMS) (v. 2020.109) programs.^44,45^ Geometry optimizations have been carried out using the OLYP functional^46^ and the slater-type TZ2P basis set, with the small frozen core approximation for the treatment of core electrons.^47^ This basis is composed of triple-ζ quality orbitals, augmented with two sets of polarization functions per atom. Scalar relativistic effects were also included by means of the zero-th order regular approximation (ZORA).^48–50^ This level of theory has been recommended for mechanistic and energy landscape investigations involving dichalcogenides^51^ and has been used with accurate results in previous studies by some of us.^7,9,52^ Solvent-assisted proton-exchange (SAPE) calculations featuring two explicit H_2_O molecules have also been computed at the ZORA-OLYP/TZ2P level. Analytical frequencies analysis has been performed for all stationary points using the same level of theory of optimization. All minima display only positive vibrational frequencies, whereas transition states (TS) have a single imaginary frequency corresponding to the normal mode leading from reactants to products. The reaction path from the transition state to the reactant and product complexes have been determined through the intrinsic reaction coordinate (IRC) procedure, as implemented in AMS 2020.^53^ To achieve a more accurate description of electronic energies, all stationary points have been evaluated by single-point calculations with the M06 meta-hybrid functional^54^ on the all-electron TZ2P basis set.^47^ Therefore, we refer to this level of theory in short as ZORA-M06/TZ2P-ae//ZORA-OLYP/TZ2P. The effect of spin–orbit coupling on the energies has been evaluated for Te-species and was found to be negligible (Table S4 in Supporting Information).

To gain insight into the differences in the energy barriers, activation strain analysis (ASA) has been carried out on the minimal models. This fragment-based approach is a quantitative tool developed for the analysis of activation barriers in all sorts of chemical reactions.^42,55^ The energy along the inverse reaction path that connects the transition state (TS) geometry to the reference state (the reactants), i.e. along a suitable reaction coordinate ζ, is separated into two contributions: the *strain energy* and the *interaction energy* (eq 1).

1 $$\Delta E\left(\zeta \right)=\Delta E_{strain}\left(\zeta \right)+\Delta E_{\mathrm{int}}\left(\zeta \right)$$

The reaction strain (Δ*E*_strain_) is a distortion contribution determined by the rigidity of the reactants, also identified with the strength of the bonds and the angles that are deformed as the reaction proceeds. In general, this is a positive term that destabilizes nonequilibrium geometries and gives rise to energy barriers. Conversely, the interaction energy (Δ*E*_int_) is, in most cases, a stabilizing term representing the actual interaction between the deformed fragments. The interplay between strain and interaction determines the energy associated with the TS and its position along the reaction coordinate. Following the energy decomposition analysis (EDA) scheme,^55,56^ Δ*E*_int_ can be further decomposed into three different terms: electrostatic interaction, orbital interaction, and Pauli repulsion (eq 2).

2 $$\Delta E_{\mathrm{int}}\left(\zeta \right)=\Delta V_{elstat}\left(\zeta \right)+\Delta E_{OI}\left(\zeta \right)+\Delta E_{Pauli}\left(\zeta \right)$$

ASA and EDA have been performed along the IRC profile by partitioning the system into two fragments, i.e., the chalcogenide and H_2_O_2_; the program PyFrag 2019 was used.^57^

When the orbital interaction was dominant, its contribution was studied following the extended transition state (ETS) NOCV decomposition method proposed by Mitoraj et al.^43^ In general, the formation of a bond can be interpreted in terms of the resulting difference between the electron density of an adduct (AB) and the individual noninteracting frozen fragments (A and B) (eq 3). By contrast, in the NOCV scheme this quantity is expressed in reference to the antisymmetrized product of the A and B wave functions (eq 4), consisting of a new set of spin–orbitals (ψ_*i*_^0^), which are obtained through the orthogonalization and renormalization of the fragments’ occupied spin–orbitals (ψ_*i*_^A^ and ψ_*i*_^B^).

3 $$\Delta \rho =\sum_{i}{\left|\psi_{i}^{AB}\right|}^{2}-\sum_{i}{\left|\psi_{i}^{A}\right|}^{2}-\sum_{i}|\psi_{i}^{B}{|}^{2}$$

4 $$\Delta \rho^{\prime }=\sum_{i}{\left|\psi_{i}^{AB}\right|}^{2}-\sum_{i}{\left|\psi_{i}^{0}\right|}^{2}$$

Then, the deformation density matrix (Δρ′) can be diagonalized in terms of NOCVs, which are the eigenfunctions of the Nalewajski-Mrozek valence operator (eq 5).^58,59^

5 $$V=\sum_{i}\left(|\psi_{i}^{AB}\rangle \left\langle \psi_{i}^{AB}\left|-\right|\psi_{i}^{0}\right\rangle \langle \psi_{i}^{0}|\right)$$

At this point, each eigenvalue pair ±*v*_k_ resulting from the diagonalization corresponds to the transfer of a fraction of electron density from a φ_–k_ orbital to a φ_k_ orbital that is occurring when the adduct is formed from the frozen fragments. Although none of the terms that have been introduced here are physical observables, the strength of this approach lies in the visualization of the change in the electronic structure of the system, due to orbital interactions, which is particularly useful to quantify donation and backdonation contributions in donor–acceptor systems,^60^ such as those examined in this article.

When specified, solvation effects have been included via a continuum approach, by means of the conductor-like screening model (COSMO).^61^ After the reoptimization of gas-phase stationary points in solution, their single-point energies have been evaluated at the COSMO-ZORA-M06/TZ2P-ae//COSMO-ZORA-OLYP/TZ2P level.

## 3 Results and Discussion

### 3.1 Effect of the Substituent Group

As reported, the functionalization at the β-position of tellurium has a nontrivial effect on the rate of thiol oxidation.^37^ To delve into this chemistry, we have studied in silico the reaction of the tellurides shown in Figure 1 with H_2_O_2_. **Te-1** has a methane-sulfonamide functional group, which allows to look at the influence of the *N*-sulfonyl moiety by comparison to the free amino-telluride **Te-2**. Due to possible acid–base equilibria between **Te-2** and thiol substrates, the oxidation of its protonated form **Te-2-H** ^**+**^ has also been considered. **Te-3** is characterized by a β-disulfide group, whereas in the case of **Te-4** the sulfur heteroatom is bonded to an allyl residue.

Table 1 reports the activation and reaction energies for the studied oxidations of the chalcogenides by H_2_O_2_. Restricting for the moment our discussion to the oxidation of the tellurides, we note that all energy values, with the notable exception of **Te-2-H** ^**+**^, particularly Δ*E*_RC_ and Δ*E*_r_, do not change much from case to case. Interestingly, the trend of the TS energies and, consequently, of the activation energies, reflects very well the experimental data on the catalytic activity of their analogs.^37^ Substitution of the *N*-sulfonyl moiety in **Te-1** with H in **Te-2** reduces the energy barrier by 2.7 kcal mol^–1^. Likewise, Δ*E* ^‡^ decreases by 3.0 kcal mol^–1^ when β-disulfide **Te-3** and β-allyl sulfide **Te-4** are compared. The case of **Te-2-H** ^**+**^ stands out because of its low-energy TS: even though the adduct with H_2_O_2_ is more stable, the activation energy is 3.2 kcal mol^–1^ lower for the protonated β-amino catalyst. By inspecting the TS structure (Figure 2, **Te-2-H** ^**+**^ **-TS**), it is observed that a hydrogen bond interaction is present between the β-NH_3_^+^ group and the oxygen atom that is approaching the Te center. Additionally, after the transition state is reached, the NH_3_^+^ substituent undergoes a deprotonation step, transferring a H^+^ to the O-atom furthest from Te and yielding H_2_O (**Te-2-H** ^**+**^ **-PC**). In the process, the positive charge is (formally) transferred from the ammonium group to the more electropositive tellurium atom. Taking both remarks into consideration, we hypothesize that this specific intramolecular interaction makes the reduction of H_2_O_2_ more favorable by influencing (decreasing) its electron density, thus enhancing its electron acceptor character. The higher experimental activity of β-amino tellurides^37^ may be then ascribed to the acidic form of the catalysts.

**Table 1:** Oxidation of Diorganochalcogenides by H_2_O_2_ ^a^

	Δ*E*_RC_	Δ*E*_TS_	Δ*E* ^‡^	Δ*E*_r_
**S-1**	–4.5	31.0	35.5	–47.2
**S-2**	–4.5	26.2	30.7	–50.2
**S-2-H** ^**+**^	–14.1	7.9	22.1	–55.3
**S-3**	–8.8	28.4	37.2	–52.0
**S-4**	–6.4	24.9	31.4	–53.6
**Se-1**	–4.5	26.2	30.7	–37.5
**Se-2**	–4.8	21.1	25.8	–40.6
**Se-2-H** ^**+**^	–13.9	5.0	19.0	–52.2
**Se-3**	–8.7	22.3	31.0	–40.7
**Se-4**	–6.6	19.7	26.2	–42.7
**Te-1**	–4.0	16.9	20.9	–47.6
**Te-2**	–6.7	11.4	18.2	–46.6
**Te-2-H** ^**+**^	–12.0	2.9	14.9	–63.9
**Te-3**	–7.0	11.6	18.5	–47.9
**Te-4**	–5.6	10.0	15.5	–47.1

a Gas-phase electronic energies (in kcal mol^–1^) of reactant complexes (RC) and transition states (TS) of the studied compounds, energy barriers and reaction energies (level of theory: ZORA-M06/TZ2P-ae//ZORA-OLYP/TZ2P). RC, TS, and reaction energies are referred to the free reactants and products. Activation energies are computed as the difference between the energies of the transition state and the reactant complex.

**Figure 2:**
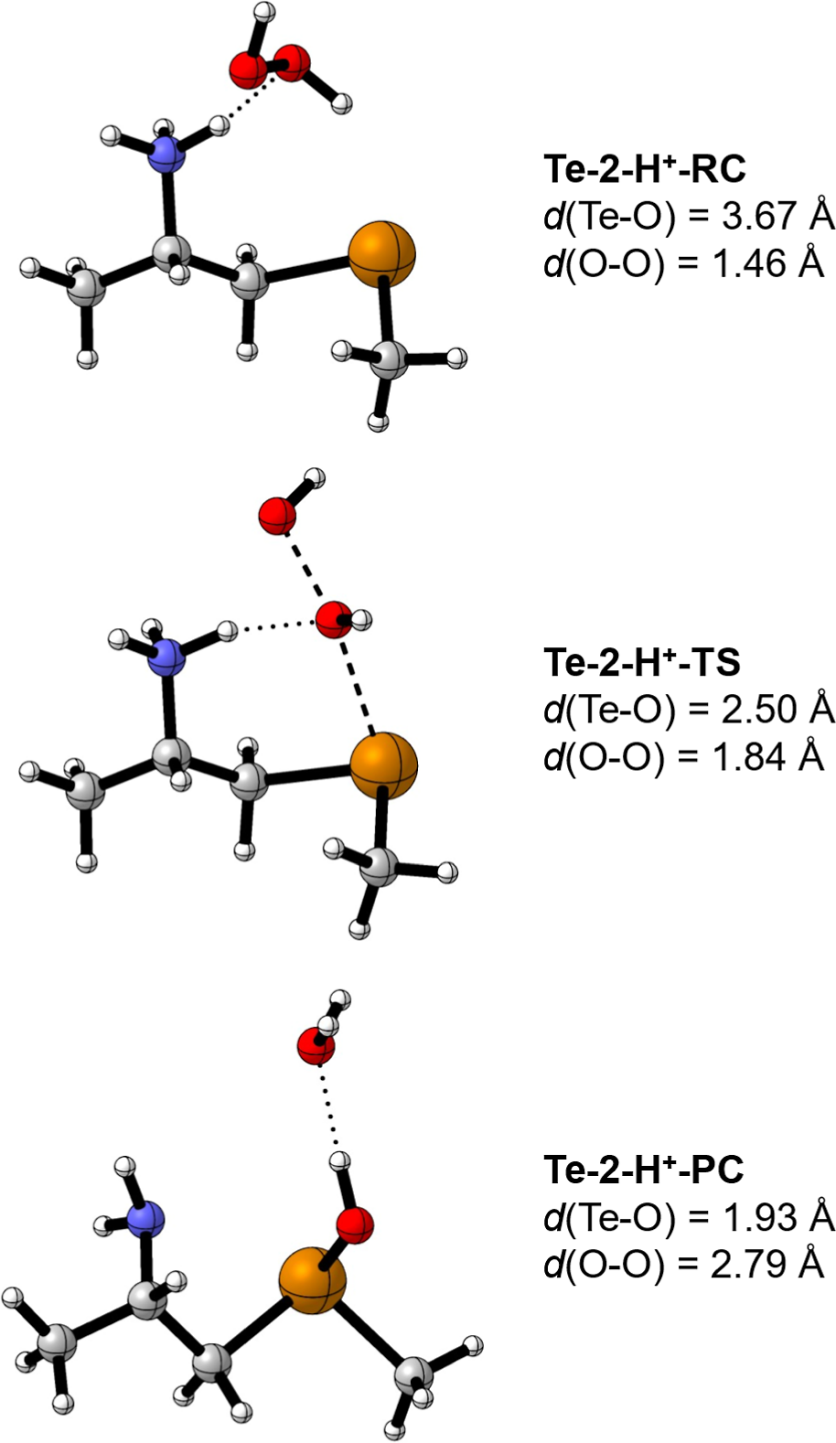
Molecular geometries of the stationary points and the corresponding Te–O and O–O interatomic distances (in Å) for the oxidation of **Te-2-H** ^**+**^ by H_2_O_2_ (level of theory: ZORA-OLYP/TZ2P).

### 3.2 Comparison with Sulfides and Selenides

In order to rationalize the chalcogen’s role in these oxidations, sulfur and selenium analogs **S-1:4** and **Se-1:4** have been studied too. By comparing these data to those of Te-species, it emerges that for all chalcogens the influence of the substituent on the activation energy is similar, except for small deviations. The effect of the *N*-sulfonyl group (cases **S-1** and **Se-1**) is almost unperturbed when the chalcogen changes and is equal to ∼5 kcal mol^–1^. Correspondingly, the energy barrier differences between β-allyl and β-disulfide species (cases **Ch-3** and **Ch-4**) range from 3.0 for Te to 5.8 kcal mol^–1^ for S. However, the protonation of β-NH_2_ chalcogenides affects more the lightest chalcogens’ derivatives: upon acquisition of H^+^, Δ*E* ^‡^ decreases by 8.6 kcal mol^–1^ for **S-2-H** ^**+**^, by 6.8 for **Se-2-H** ^**+**^ and by 3.3 for **Te-2-H** ^**+**^, respectively. This trend correlates with the increasing metallic character of the chalcogen, i.e., a stronger tendency to acquire and stabilize a positive charge.

As expected, the nature of the chalcogen has a remarkable influence on the activation energies, although its extent is highly case-dependent. For example, the energy barrier for any selenide is always higher if compared to its corresponding telluride, varying in a range from 4 to 12 kcal mol^–1^. As a general observation, we point out that the chalcogen effect is more pronounced when the β-heteroatom is sulfur, rather than nitrogen.

### 3.3 Activation Strain and Energy Decomposition Analysis

To identify the reasons behind the deactivating effect of the methanesulfonyl functionalization on the nitrogen atom, ASA was performed (Figure 3, graph A). The energy profile from the reactants to the TS is significantly higher for **Te-1** than for **Te-2**. The difference in Δ*E* ^‡^ is determined by the interaction, rather than by the distortion. The decomposition of the former contribution reveals that it is ascribed to the more strongly stabilizing orbital (Δ*E*_OI_) and to a lesser extent to the electrostatic interaction (Δ*V*_elstat_) (Figure 3, graph B). The slightly higher value of strain energy is due to the stronger deformation of the H_2_O_2_ fragment along the reaction coordinate in the case of **Te-2**.

**Figure 3:**
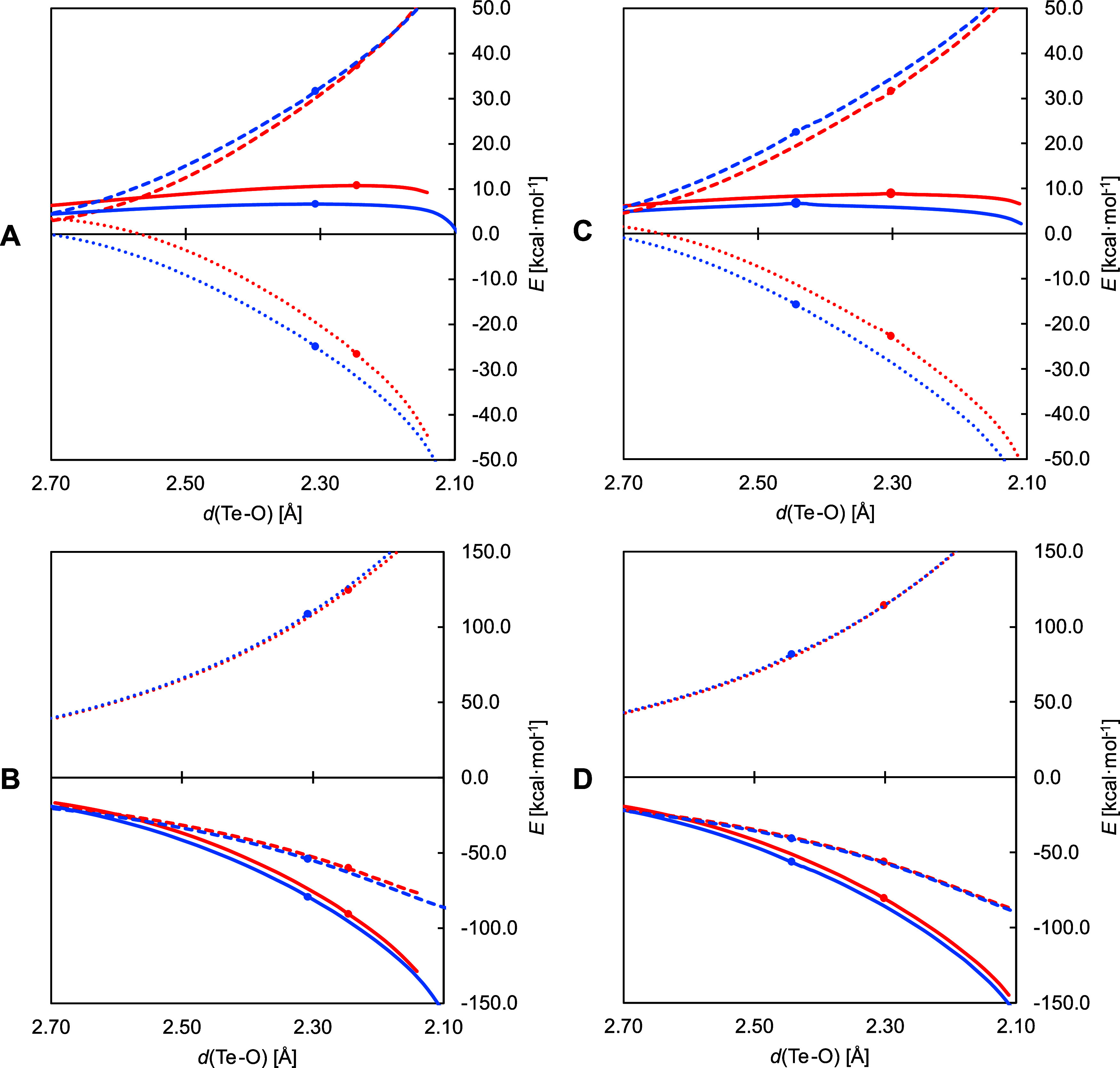
ASA and EDA plots comparing the oxidation by H_2_O_2_ of **Te-1** (red) and **Te-2** (blue) (graphs A and B), and of **Te-3** (red) and **Te-4** (blue) (graphs C and D); the reaction coordinate is the Te–O distance (in Å). ASA (graphs A and C): total energy (solid lines), strain (dashed lines) and interaction (dotted lines); EDA (graphs B and D): Pauli repulsion (dotted lines), electrostatic interaction (dashed lines) and orbital interaction (solid lines) (level of theory: ZORA-OLYP/TZ2P). TS position is denoted by a dot.

The different effects of disulfide and *S*-allyl β-functional groups on the reaction were also elucidated by ASA carried out on the energy profiles of the oxidations by H_2_O_2_ of **Te-3** and **Te-4** (Figure 3, graph C). Similarly to the previous case, the total energy is higher for **Te-3** along the whole IRC path, and its transition state is reached at a shorter Te–O distance. Although the energy differences are quite narrow, the interaction contribution is clearly decisive in this comparison too, since the strain follows the opposite trend, favoring **Te-3** over **Te-4**. From EDA, it resulted that the individual contributions to the total Δ*E*_int_ are numerically close in the two cases (Figure 3, graph D). However, while electrostatic and Pauli terms (Δ*V*_elstat_ and Δ*E*_Pauli_, respectively) tend to cancel each other out, the magnitude of the difference in orbital interaction between **Te-3** and **Te-4** is larger and is worth ∼5 kcal mol^–1^ at both transition states.

### 3.4 NOCV Analysis

After recognizing its significance, the nature of the orbital interaction has been further examined by inspecting the MOs that are involved in the studied reactions. The stabilizing effect of Δ*E*_OI_ largely arises from the charge transfer from the HOMO of the telluride fragment (the reductant) to the LUMO of the H_2_O_2_ fragment (the oxidant) (Figure 4). While the former can be reasonably identified with a lone pair localized on the Te atom, the latter corresponds to the σ* antibonding orbital associated with the O–O bond (Figure 5). To delve into this effect, for all tellurides the fragments’ electronic structures have been inspected at a consistent point along the main reaction coordinate (the Te–O distance) rather than at the TS, to allow comparisons among all four reactions. Furthermore, the orbital interaction has been decomposed according to the ETS-NOCV scheme. The energies of HOMOs, LUMOs and the corresponding Δ*E*_OI_ terms, along with the EDA terms, are reported in Table 2. There is a correlation between the orbital interaction and the activation energies from Table 1. The tellurides displaying the highest (**Te-1**) and lowest (**Te-4**) energy barriers are associated with the least negative and largest negative total orbital interaction (Δ*E*_OI_), respectively. In contrast, **Te-2** and **Te-3** display very similar energy values. The analysis of NOCVs points out that the deformation density associated with the HOMO–LUMO charge transfer between the fragments accounts for most of the stabilization (Figure S1, Table S3 in Supporting Information). Intuitively, even though their relative energy difference might vary significantly along the reaction path, the interaction is also greater when the HOMO of the telluride fragment is more destabilized or, likewise, when the LUMO of the peroxide fragment is more stabilized.

**Figure 4:**
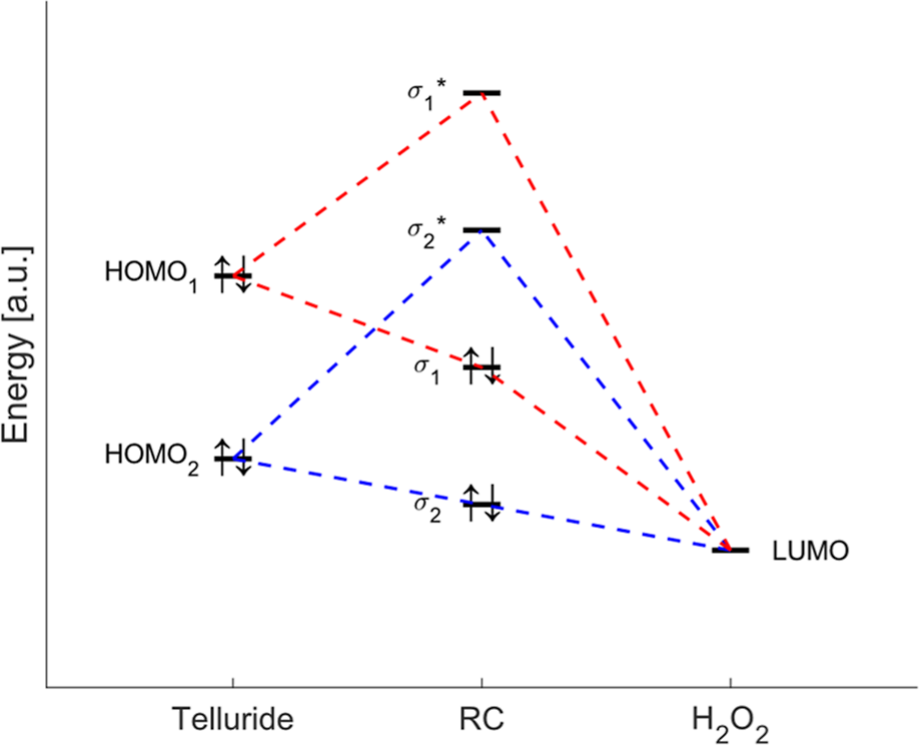
Schematic representation of the MO interaction diagram between the telluride fragment (left) and the H_2_O_2_ fragment (right), to illustrate the effect of deactivating (red) and activating (blue) groups on the energy levels. The substituents influence the charge transfer from the HOMO of the donor to the LUMO of the acceptor: the lower the HOMO of the telluride is (compared to the LUMO of H_2_O_2_), the less negative the resulting orbital interaction.

**Figure 5:**
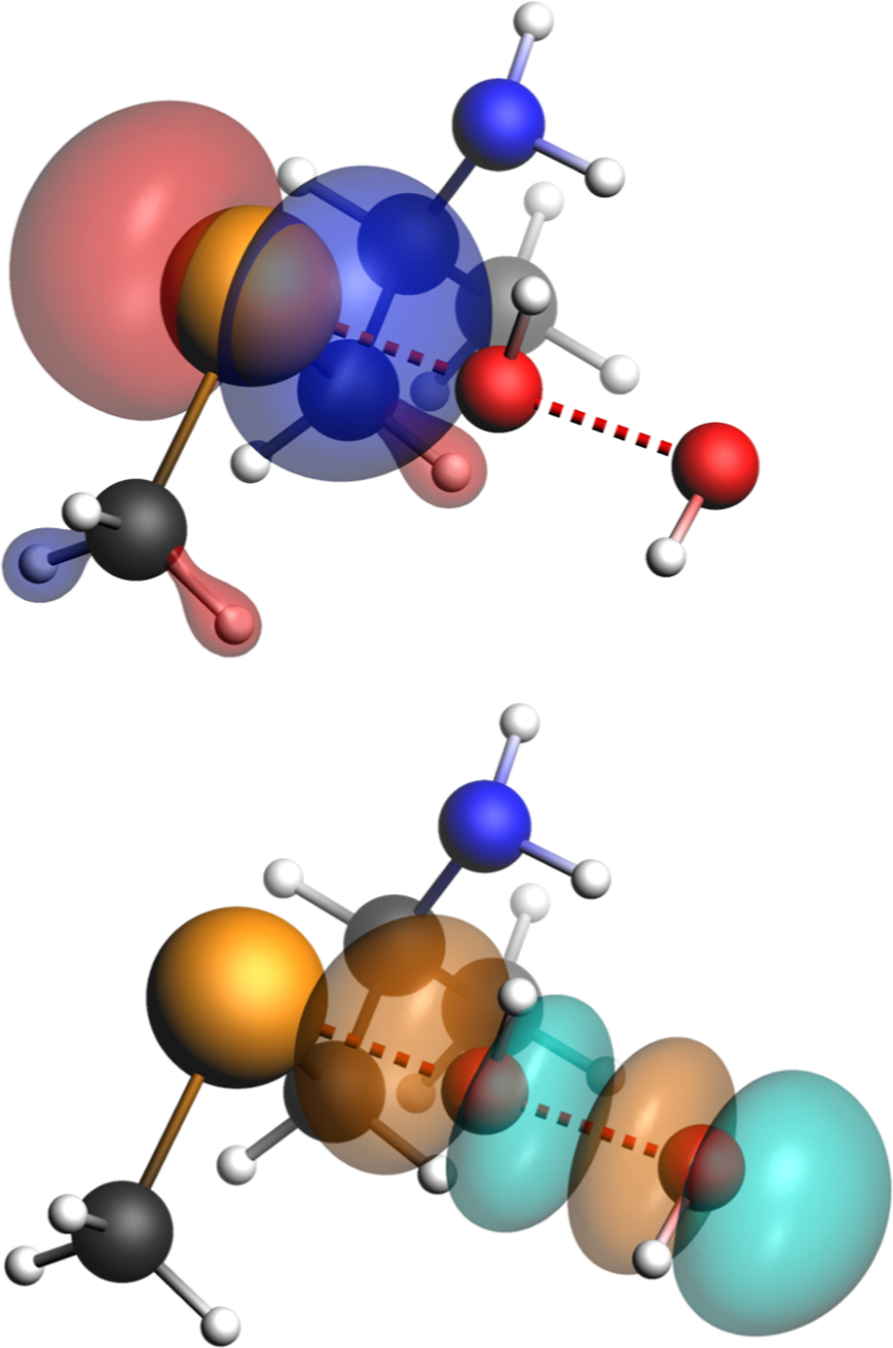
Fragments’ orbitals at the TS of **Te-2** (ρ > 0.05): HOMO of the telluride fragment (top) and LUMO of the H_2_O_2_ fragment (bottom) (level of theory: ZORA-OLYP/TZ2P).

**Table 2:** HOMO, LUMO and ETS-NOCV Analysis

*d*(Te–O) [Å]^a^	2.45			
reaction	**Te-1**	**Te-2**	**Te-3**	**Te-4**
Δ*E*_Int_ ^b^	–3.2	–12.8	–11.1	–15.8
Δ*E*_OI_ ^c^	–36.7	–50.0	–50.8	–56.4
*E*_OI,k_ ^d^	–30.4	–43.0	–42.8	–48.1
Δ*E*_HOMO–LUMO_ ^e^	+12.0	–19.4	–13.4	–24.4

a Comparison between relevant fragment orbitals and orbital interaction contributions of the studied tellurides: Te–O distance considered for the decomposition.

b Total interaction energy from ASA.

c Total orbital interaction from EDA.

d NOCV energy contribution associated with the HOMO–LUMO interaction.

e HOMO–LUMO energy difference (*E*_LUMO_ – *E*_HOMO_), (level of theory: ZORA-OLYP/TZ2P). All energies are in kcal mol^–1^.

### 3.5 Effect of the Chalcogen

To complete the analysis of oxidations of diorganochalcogenides by H_2_O_2_ we looked at the more fundamental effect of the chalcogen atom, focusing on the reaction of the β-amino compounds **S-2**, **Se-2** and **Te-2**. This reaction was chosen due to the greater simplicity of the β-amino chalcogenides; by doing so, the inclusion of more complex substituents is avoided, as they would ultimately make the analysis more challenging. In this case, the absolute chalcogen–oxygen distance does not represent the best choice for the ASA reaction coordinate. Due to the significant differences of atomic radii, interatomic distances vary drastically and the energy profiles end up being too displaced from each other for the analysis to be meaningful. Adopting the peroxide oxygen–oxygen bond extension as the reaction coordinate represents an intuitive alternative. However, this issue would persist, because the O–O bond breaks to a similar extent but at a greater distance when the center is Te rather than S. Thus, at any point along this projection of the PES, the different chalcogen centers would be at considerably different distances from the H_2_O_2_ fragment, which may lead to misinterpretations of the energy terms, especially those which contribute to the interaction energy. Hence, we have chosen a translated chalcogen–oxygen distance (Δ*d*) as our reaction coordinate, by subtracting from the absolute distance the corresponding equilibrium bond distance of the Ch=O bond in the oxide product, namely 1.50 Å for S=O, 1.66 Å for Se=O and 1.83 Å for Te=O. In doing so, it is possible to see that all three TS occur at similar values of Δ*d*, between 0.49 and 0.44 Å (Figure 6, graph A). This corroborates the assumption that the Ch–O distance relative to the formed chalcogenoxide bond is a sensible reaction coordinate. Most interestingly, in this region of the plot, the interaction energies do not follow the trend of the activation energies (S > Se > Te) and do not differ dramatically from case to case. EDA also shows that only the differences in Pauli repulsion correspond to the expected progression; conversely, electrostatic and orbital interactions are more stabilizing in the case of sulfur (Figure 6, graph B). As such, the key-factor that determines the energy barrier is strain, which is almost exclusively due to the deformation of the H_2_O_2_ fragment (Figure 6, graph C). In proximity of the TS, when the Ch–O bond is formed to a similar extent, the peroxide fragment is more distorted when it is reacting with a sulfide compared to a selenide and, likewise, with a selenide compared to a telluride. In other words, for the chalcogenoxide to be formed, the smaller the chalcogen is, the more energy is “spent” to distort H_2_O_2_ and make it react—a penalty that is not compensated by the two fragments’ interaction.

**Figure 6:**
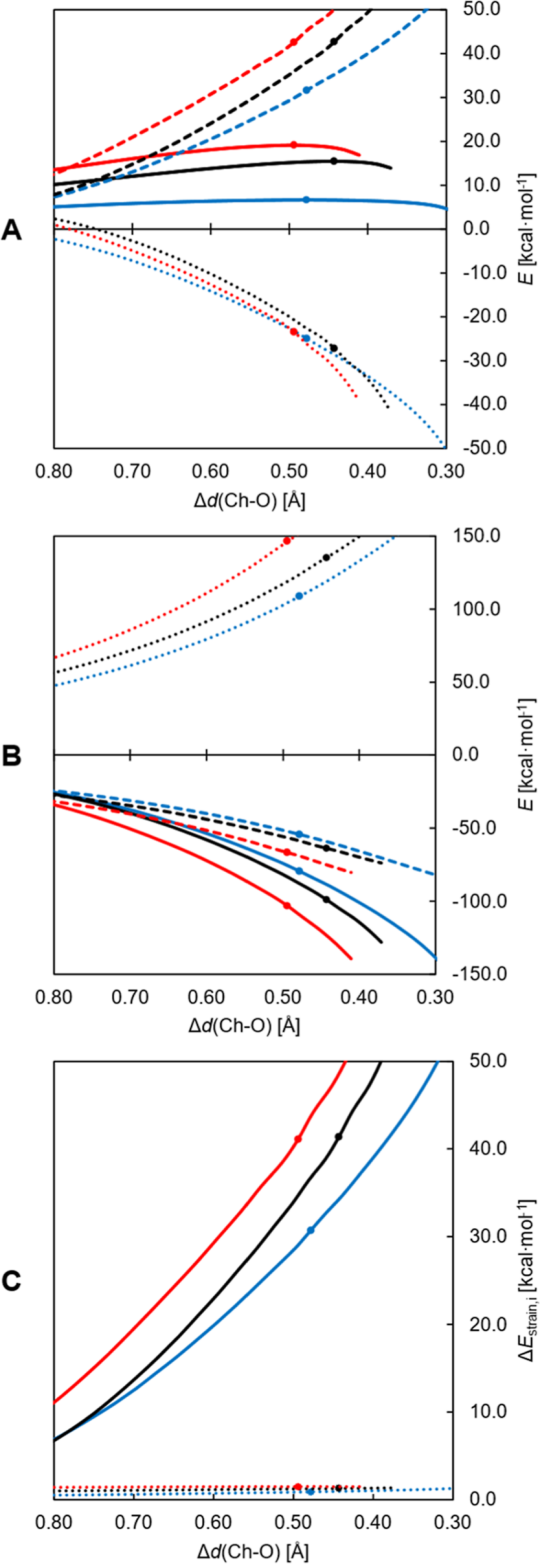
ASA (A), EDA (B) and strain energy decomposition (C) plots for the oxidation of **S-2** (red), **Se-2** (black) and **Te-2** (blue) by H_2_O_2_, along the translated Ch-O distance (Δ*d*, in Å) (level of theory: ZORA-OLYP/TZ2P). ASA: total energy (solid lines), strain (dashed lines) and interaction (dotted lines); EDA: Pauli repulsion (dotted lines), electrostatic interaction (dashed lines) and orbital interaction (solid lines); strain energy decomposition: H_2_O_2_ fragment strain energy (solid lines) and chalcogenoxide fragment strain energy (dotted lines). TS coordinates are denoted by a dot.

### 3.6 Solvation Effects and SAPE

Finally, to establish a possible role of the solvent on the energy barriers computed so far, we have modeled the oxidation of all species in aqueous solution. In a polar medium, all energy barriers are considerably lower than in gas-phase, apart from the case of protonated catalysts **S-2-H** ^**+**^, **Se-2-H** ^**+**^, and **Te-2-H** ^**+**^, when the TS energy becomes much higher (Table 3). As a result, all β-NH_2_ chalcogenides display lower activation energies than their acidic counterparts. This is due to the positively charged chalcogenide fragments being more stabilized by the solvent due to more negative solvation energies. In all other instances, gas-phase trends for the effects of the chalcogen atom and of the functional group are preserved in solution, although it must be emphasized that energy differences are modest, often even less than 2 kcal mol^–1^.

**Table 3:** Oxidation of Diorganochalcogenides by H_2_O_2_ in Aqueous Solution^a^

	Δ*G*_TS_		
	Te	Se	S
1	8.1	13.5	19.3
2	5.6	10.0	14.4
2-H^+^	7.9	13.8	17.7
3	10.5	14.4	19.0
4	7.9	12.8	15.7

a Transition state Gibbs free energies (in kcal mol^–1^) in H_2_O solution of the studied compounds, relative to the free solvated reactants (level of theory: COSMO-ZORA-M06/TZ2P-ae//COSMO-ZORA-OLYP/TZ2P).

To explore how the solvent affects the mechanism of oxidation, we have further modeled the reaction employing the so-called solvent-assisted proton-exchange approach (Scheme 2). In this mechanism, a network of H_2_O molecules connected by H-bonds allows the shuttling of a proton in a concerted manner, providing a closer description of what would happen in aqueous solution. This methodology has been extensively applied by Bayse et al. to organochalcogen reactivity^62–65^ and by Orian et al.^66–69^ Figure 7 shows the RC, TS, and PC structures for the two representative reactions of β-amino tellurides **Te-2** and **Te-2-H** ^**+**^. In the simple yet exemplary case of **Te-2**, the reactants display the anticipated arrangement: a complex stabilized by three H-bonds with the solvent molecules is initially formed (**Te-2-RC**), which then leads to a TS where three protons are being transferred (**Te-2-TS**). The distortion the reactants undergo to react is diminished by the inclusion of explicit solvent molecules, causing the energy barriers for the oxidation to drop by much (Table 4). This configuration remains virtually identical when Te is replaced by S or Se or the β-substituent changes. However, this is not the case for the protonated species **S-2-H** ^**+**^, **Se-2-H** ^**+**^, and **Te-2-H** ^**+**^, when the most favorable arrangement is achieved in a different way (Figure 7). In the example of **Te-2-H** ^**+**^ **-TS**, the β-NH_3_^+^ group is involved in the H-bond network, stabilizing the positive charge. No concerted H^+^ exchange occurs, aside from the transfer of a proton between the oxygen atoms of H_2_O_2_ (from the closest to the farthest with respect to Te). Despite being stabilized by an intramolecular H-bond between Te and β-NH_3_^+^, the persistence of the PC in solution is unlikely, as it would rapidly hydrate to dihydroxytellurane. Overall, gas-phase trends hold for the SAPE model too, both in terms of substituents’ and chalcogen’s effects.

**Scheme 2:**
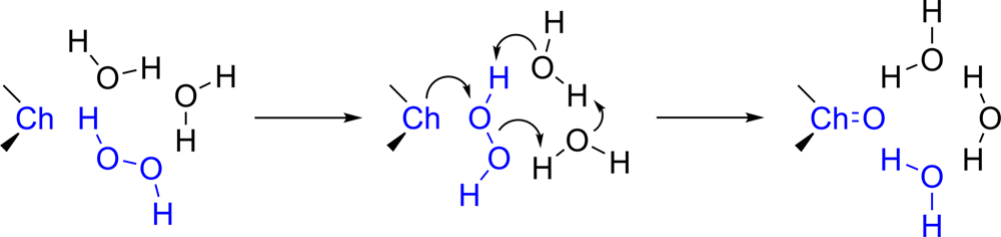
SAPE Model for the Oxidation of Diorganochalcogenides by H_2_O_2_

**Figure 7:**
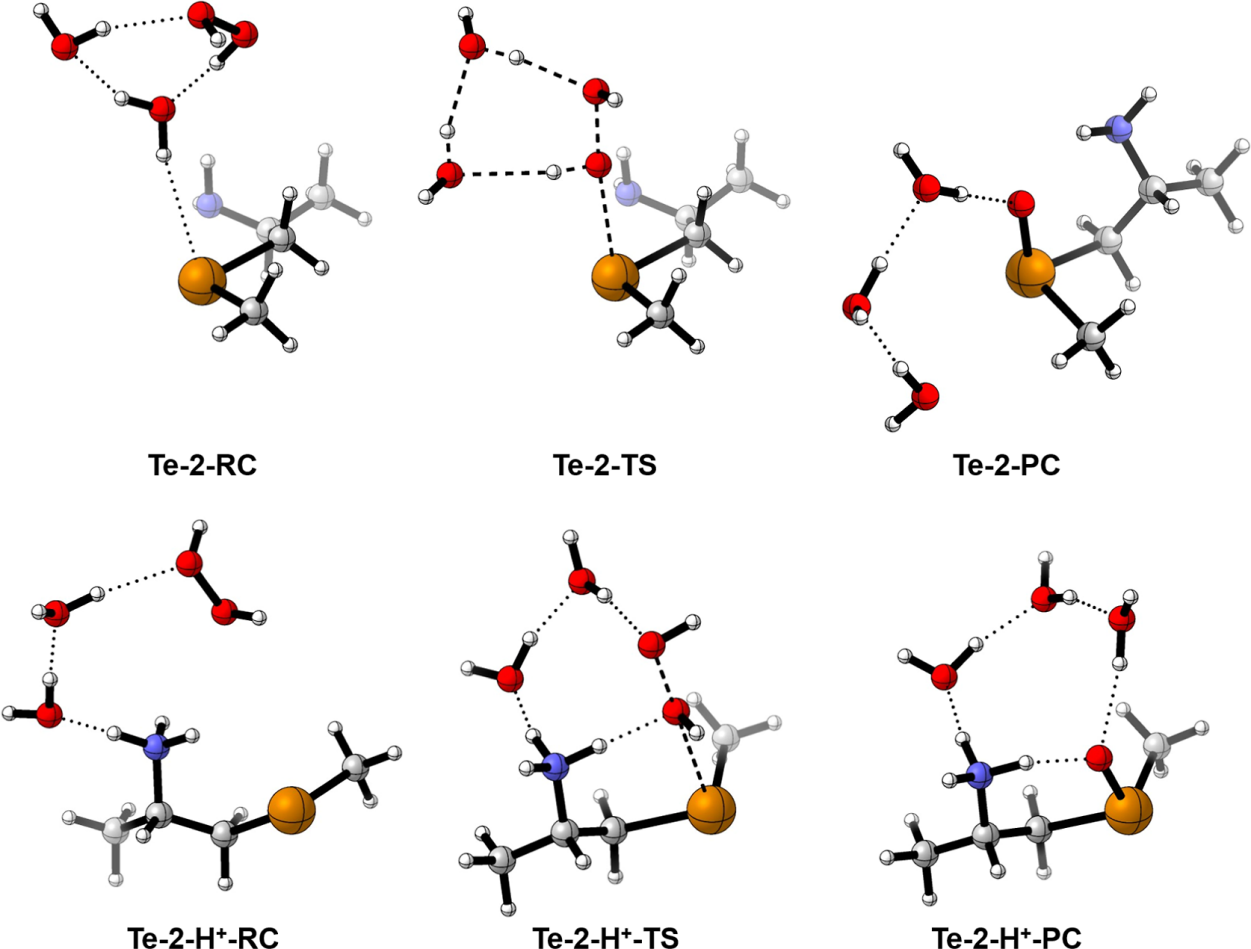
Stationary points for the oxidation of tellurides **Te-2** and **Te-2-H** ^**+**^ by H_2_O_2_ according to the SAPE mechanism (level of theory: ZORA-OLYP/TZ2P).

**Table 4:** Oxidation of Diorganochalcogenides by H_2_O_2_ Along the SAPE Pathway^a^

	Δ*E* ^‡^		
	Te	Se	S
1	17.1 (20.9)	23.0 (30.7)	27.5 (35.5)
2	11.3 (18.2)	19.2 (25.8)	23.1 (30.7)
2-H^+^	7.7 (14.9)	13.6 (19.0)	19.0 (22.1)
3	12.6 (18.5)	21.1 (31.0)	23.8 (37.2)
4	10.4 (15.5)	16.2 (26.2)	19.3 (31.4)

a Activation energies (in kcal mol^–1^) of the studied compounds for the SAPE pathway, relative to the reactant complex (level of theory: ZORA-M06/TZ2P-ae//ZORA-OLYP/TZ2P). Bracketed values are gas-phase activation energies from Table 1 for comparison.

## 4 Conclusions

In this study, the oxidation mechanism of functionalized diorganotellurides and their sulfur and selenium analogs by H_2_O_2_ has been investigated by a computational approach, elucidating the role of β-position substituents in modulating the GPx-like activity. In particular, the protonation of the β-amino chalcogenides lowers significantly the activation energies. The substituent effect is ascribed to a difference in orbital interaction between the reactants’ fragments. NOCV comparative analysis reveals that activating groups behave as such by increasing the energy of the chalcogenide’s HOMO compared with the LUMO of H_2_O_2_, i.e., decreasing the HOMO–LUMO gap and enhancing the charge-transfer between the two orbitals. Expectedly, the oxidation of sulfides and selenides is less facile than that of the corresponding tellurides. ASA applied to the oxidation of **S-2**, **Se-2**, and **Te-2** reveals that this is in fact a consequence of the difference in strain energy contributions to the activation energy. At the TS, when the chalcogenoxide bond is about to be formed, the energy required to deform H_2_O_2_ is the highest in the case of sulfides and the lowest in the case of tellurides. The comparative evaluation of the chalcogen reactivity highlights the subtle interplay between electronic and geometric factors that steers the oxidation mechanisms, expanding the current knowledge on the reactivity of chalcogenides with H_2_O_2_. By elucidating solvation and protonation effects, a deeper understanding of the influence of varying conditions is also provided. Even though both the inclusion of continuum solvation effects and of explicit water molecules lowers significantly the activation energies, gas-phase trends are preserved in solution. Collectively, these findings offer useful insight into develop and optimize organochalcogen catalysts for the activation of hydroperoxides in synthetic and pharmaceutical applications.

## Supplementary Material



## Notes

Single point energies of gas-phase, COSMO and SAPE geometries; EDA and ETS-NOCV supporting information; Cartesian coordinates, electronic energies and imaginary frequencies (PDF)
