# Supplementary material for: Glutathione Peroxidase-Like Activity of Functionalized Tellurides: Insights into the Oxidation Mechanism Through Activation Strain Analysis
Source: Inorg Chem. 2025 May 9;64(20):10022–31. doi: 10.1021/acs.inorgchem.5c00581 (PMC12117569; doi:10.1021/acs.inorgchem.5c00581)
Supplement: Supplementary file 1 [file ic5c00581_si_001.pdf]

Supporting Information

Glutathione Peroxidase-like Activity of Functionalized Tellurides: Insights into the Oxidation Mechanism through Activation Strain Analysis

Alessandro Rubbi,[†] Damiano Tanini,[‡] Antonella Capperucci,[‡] Laura Orian*[†]

[†] Dipartimento di Scienze Chimiche, Università degli Studi di Padova, via Marzolo 1, 35131 Padova, Italy.

[‡] Dipartimento di Chimica "Ugo Schiff", Università di Firenze, via della Lastruccia 3-13, 50019 Sesto Fiorentino, Italy.

TABLE OF CONTENTS

Table S1. Gas-phase single point energies S2
Table S2. Single point energies in solution S2
Table S3. EDA and ETS-NOCV data S3
Table S4. EDA and ETS-NOCV cartesian coordinates S3
Figure S1. Deformation densities S5
Table S5: Spin-orbit coupling S6
Table S6: SAPE single point energies S7
Table S7. Gas-phase cartesian coordinates S8
Table S8. COSMO cartesian coordinates S32
Table S9. SAPE cartesian coordinates S42

Table S1. Gas-phase single point energies.

Gas-phase single-point electronic energies relative to the atomic fragments (in kcal·mol^{-1}) of compounds reported in Table 1 of the main text (coordinates at Table S7) (level of theory: ZORA-M06/TZ2P-ae//ZORA-OLYP/TZ2P).

	**R**	**RC (H_2O_2)**	**TS**	**P**
S-1	-3423.9	-3990.5	-3955.0	-3616.0
S-2	-2439.9	-3006.5	-2975.8	-2635.0
S-2-H$^+$	-2395.3	-2971.5	-2949.4	-2595.5
S-3	-5527.8	-6098.6	-6061.5	-5724.6
S-4	-3969.4	-4537.9	-4506.6	-4167.9
Se-1	-3402.5	-3969.0	-3938.3	-3584.8
Se-2	-2418.6	-2985.4	-2959.5	-2604.1
Se-2-H$^+$	-2374.8	-2950.7	-2931.8	-2571.8
Se-3	-5485.7	-6056.4	-6025.4	-5671.3
Se-4	-3949.4	-4518.0	-4491.8	-4136.9
Te-1	-3374.6	-3940.6	-3919.7	-3567.1
Te-2	-2390.8	-2959.6	-2941.5	-2582.4
Te-2-H$^+$	-2348.0	-2922.1	-2907.2	-2556.8
Te-3	-5431.9	-6000.9	-5982.4	-5624.6
Te-4	-3923.8	-4491.4	-4475.9	-4115.8
H_2O	-417.2			
H_2O_2	-562.0			

Table S2. Single point energies in solvent.

Solution single-point electronic energies relative to the atomic fragments (in kcal·mol^{-1}) of compounds reported in Table 4 (coordinates at Table S8) (level of theory: COSMO-ZORA-M06/TZ2P-ae//COSMO-ZORA-OLYP/TZ2P).

	**R**	**TS**
S-1	-3434.2	-3983.7
S-2	-2443.1	-2997.6
S-2-H$^+$	-2455.1	-3006.2
S-3	-5535.2	-6085.0
S-4	-3975.0	-4528.1
Se-1	-3412.6	-3968.0
Se-2	-2422.0	-2980.8
Se-2-H$^+$	-2435.3	-2931.8
Se-3	-5492.7	-6047.1
Se-4	-3954.8	-4510.8
Te-1	-3385.8	-3946.4
Te-2	-2395.0	-2958.3
Te-2-H$^+$	-2408.1	-2969.1
Te-3	-5438.9	-5997.2
Te-4	-3928.7	-4489.6
H_2O	-423.3	
H_2O_2	-568.8	

Table S3. EDA and ETS-NOCV data

Additional data on the EDA and ETS-NOCV analysis on tellurides Te-1:4 reported in Table 2 of the main text: [a] interaction energy, [b] Pauli repulsion, [c] electrostatic interaction and [d] orbital interaction from EDA; [e] largest deformation density contribution to ΔE_{OI}, [f] module of its corresponding eigenvalue (in Lowdin) and [g] sum of all the other deformation density contributions, from ETS-NOCV. The analysis has been performed at a consistent *d*(Te-O) reaction coordinate of ~2.45 Å, as obtained from the IRC profile, to allow for a comparison between all four cases.

Reaction	ΔE_{Int} [a]	ΔE_{Pauli} [b]	ΔV_{Elstat} [c]	ΔE_{OI} [d]	$E_{OI,i}$ [e]	$\lvert\kappa_i\rvert$ [f]	$\sum_{j\neq i} E_{OI,j}$ [g]
Te-1	-3.2	65.8	-32.3	-36.7	-30.4	0.707	-6.2
Te-2	-12.8	75.2	-38.0	-49.9	-43.0	0.827	-6.9
Te-3	-11.1	79.3	-39.6	-50.8	-42.8	0.830	-7.9
Te-4	-15.8	81.7	-41.0	-56.4	-48.1	0.882	-8.3

Table S4. EDA and ETS-NOCV cartesian coordinates

Cartesian Coordinates (in Å) of the geometries reported in Table 2 of the main text and Table S3 (level of theory: ZORA-OLYP/TZ2P).

Te-1

C	-0.297900000	0.156500000	2.572300000
H	0.666300000	0.313500000	2.079600000
H	-0.687500000	-0.821000000	2.273300000
C	-0.157800000	0.222900000	4.098700000
H	-1.154500000	0.082700000	4.530900000
H	-1.003500000	0.910100000	2.214700000
C	0.755100000	-0.906700000	4.585300000
H	0.366500000	-1.873900000	4.261700000
H	1.769300000	-0.798400000	4.188000000
Te	1.056000000	-1.117900000	6.730300000
C	-0.998900000	-1.434100000	7.271800000
H	-1.577200000	-0.530000000	7.088600000
H	-1.378600000	-2.284600000	6.705500000
H	-0.998000000	-1.666600000	8.337800000
N	0.397400000	1.526900000	4.533300000
H	0.921300000	1.438200000	5.402800000
S	-0.606300000	2.859400000	4.759900000
O	-1.993800000	2.471200000	4.593300000
O	-0.146400000	3.502300000	5.975000000
C	-0.182700000	3.941600000	3.397400000
H	-0.417300000	3.451500000	2.452800000
H	-0.794100000	4.837600000	3.522600000
H	0.877300000	4.187600000	3.461700000
O	1.187900000	-5.300300000	6.344100000
O	1.162300000	-3.596100000	6.544800000
H	2.122400000	-3.582200000	6.675600000
H	0.692100000	-5.495000000	7.154100000

Te-2

C	-0.876600000	-0.551800000	2.105800000
H	0.012300000	-1.143600000	1.862400000
H	-1.744200000	-1.221300000	2.096500000
C	-0.722100000	0.146300000	3.468700000
H	-1.637200000	0.724100000	3.654800000
H	-1.020500000	0.188600000	1.311700000
C	-0.574300000	-0.873900000	4.596400000
H	-1.498800000	-1.428100000	4.769000000
H	0.244500000	-1.574400000	4.410900000
Te	-0.035000000	0.139800000	6.432400000
C	-0.433100000	-1.565400000	7.694700000
H	-1.487900000	-1.837000000	7.641600000
H	0.205600000	-2.388000000	7.371400000
H	-0.175400000	-1.276600000	8.715400000
N	0.385000000	1.106000000	3.558600000
H	0.293900000	1.830800000	2.852400000
H	1.277500000	0.642500000	3.401700000
O	3.802800000	-1.428600000	5.535400000
O	2.213700000	-0.733200000	6.005300000
H	2.675600000	0.072800000	6.278500000
H	3.790200000	-2.105300000	6.228000000

Te-3

C	-0.628100000	1.819400000	1.357900000
H	0.398700000	1.571300000	1.053300000
H	-1.289100000	1.005300000	1.016300000
C	-0.675300000	1.942300000	2.883800000
H	-0.024700000	2.758500000	3.199200000
C	-2.087700000	2.195700000	3.397400000
H	-2.479100000	3.109100000	2.946200000
H	-2.764600000	1.369100000	3.166900000
Te	-2.207200000	2.522500000	5.547400000
C	-3.739800000	4.029600000	5.422100000
H	-3.315800000	4.901400000	4.923900000

H	-4.603300000	3.634400000	4.886300000
H	-4.020900000	4.281300000	6.446100000
O	-1.026300000	3.060700000	0.791500000
C	-0.887200000	3.092200000	-0.618500000
H	0.157900000	2.936200000	-0.927400000
H	-1.514500000	2.330500000	-1.108500000
H	-1.210500000	4.083000000	-0.947800000
S	-0.033700000	0.400500000	3.686300000
S	1.993200000	0.460900000	3.491500000
C	2.488300000	0.684100000	6.218100000
H	1.463500000	0.295300000	6.307500000
H	3.175000000	-0.178400000	6.219700000
C	2.630400000	1.467600000	4.908300000
H	2.022900000	2.372500000	4.964800000
C	4.087900000	1.824800000	4.601700000
H	4.562400000	2.188400000	5.515200000
H	4.652800000	0.949600000	4.267200000
Te	4.465300000	3.374300000	3.114700000
C	4.404000000	5.035500000	4.501100000
H	3.394000000	5.162100000	4.888200000
H	5.127300000	4.869700000	5.300000000
H	4.693400000	5.916800000	3.924600000
O	2.775100000	1.557200000	7.303500000
C	2.686400000	0.914700000	8.562500000
H	1.676300000	0.516100000	8.748500000
H	3.407800000	0.086700000	8.650700000
H	2.916000000	1.667200000	9.321400000
O	-0.531800000	4.279500000	5.254800000
O	0.665300000	5.599500000	4.973300000
H	0.593200000	5.541200000	4.010300000
H	-0.042900000	4.059300000	6.061100000

Te-4

S	2.508900000	-0.778500000	0.875500000
C	0.414800000	-2.124400000	-0.263600000
H	1.124700000	-2.459300000	-1.032200000
H	0.470700000	-2.834200000	0.579100000
C	0.799200000	-0.715300000	0.187200000
H	0.835600000	-0.065600000	-0.687900000
C	-0.121300000	-0.102500000	1.239800000
H	-0.244500000	-0.753900000	2.108100000
H	0.272800000	0.858300000	1.571700000
Te	-2.135800000	0.404700000	0.544600000
C	-2.332200000	2.109100000	1.853800000
H	-1.608500000	2.867000000	1.553300000
H	-3.347600000	2.492800000	1.738800000
H	-2.171900000	1.797400000	2.887100000
O	-0.908200000	-2.095100000	-0.790100000
C	-1.345800000	-3.350800000	-1.279600000
H	-2.364300000	-3.212600000	-1.651400000
H	-0.709600000	-3.704500000	-2.104900000
H	-1.354500000	-4.116600000	-0.488400000
C	3.351800000	0.575600000	-0.050100000
H	2.711400000	1.461100000	-0.040200000
H	4.223400000	0.785400000	0.581200000
C	3.774100000	0.205800000	-1.435900000
H	4.530400000	-0.576600000	-1.502600000
C	3.292900000	0.761400000	-2.550900000
H	2.517100000	1.522500000	-2.533600000
H	3.657100000	0.448000000	-3.526600000
O	-0.921100000	1.674700000	-1.152600000
O	0.179400000	2.421400000	-2.420100000
H	-1.519400000	1.455200000	-1.881400000
H	0.079600000	3.323600000	-2.085000000

Figure S1. Deformation densities

Deformation densities (ρ>0.003) of tellurides Te-1:4 associated with the largest orbital interaction contribution from ETS-NOCV decomposition: blue and red colors indicate a positive and a negative sign respectively (level of theory: ZORA-OLYP/TZ2P). The analysis has been performed at a consistent *d*(Te-O) reaction coordinate of ~2.45 Å, as obtained from the IRC profile, to allow for a comparison between all four cases.

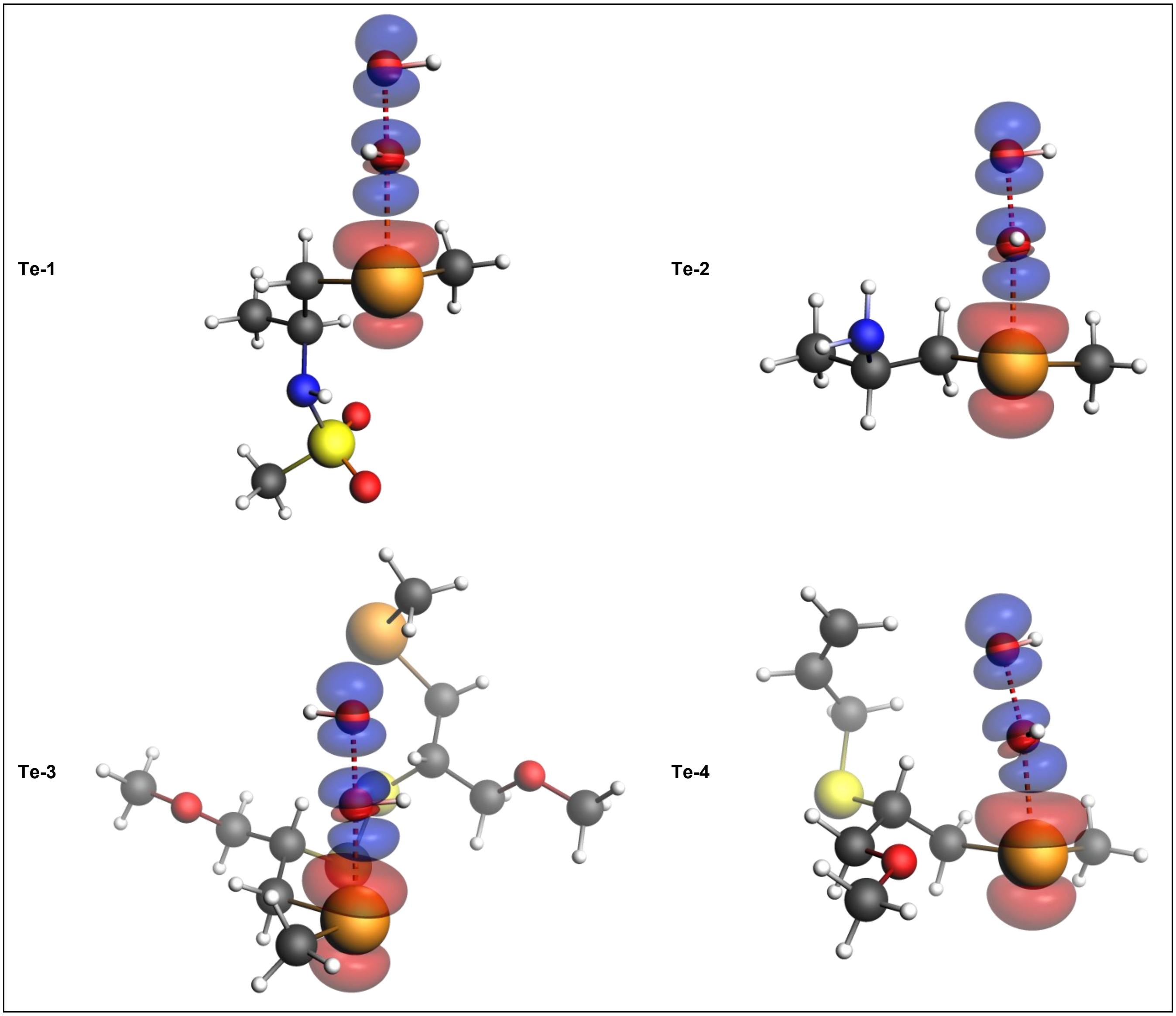

Table S5. Spin-orbit coupling

Effect of spin-orbit coupling on the energy of Te-geometries reported in Table 1 in the main text: (a) gas-phase single-point electronic energies relative to the atomic fragments (in kcal·mol^{-1}) computed on Te-geometries reported in Table 1 of the main text (level of theory: SO-ZORA-M06/TZ2P-ae//ZORA-OLYP/TZ2P); (b) energies of RC, TS and free products relative to the reactants; (c) deviation of ΔE_{so} from the corresponding scalar-ZORA values from Table 1 of the main text.

	E_{so} [a] [kcal·mol^{-1}]	ΔE_{so} [b] [kcal·mol^{-1}]	$\Delta\Delta E_{so}$ [c] [kcal·mol^{-1}]
Te-1	-4657.5	0.0	
Te-1-RC	-5223.5	-4.0	-0.007
Te-1-TS	-5202.6	17.0	0.039
Te-1-P	-4849.8	-47.4	0.236
Te-2	-3673.4	0.0	
Te-2-RC	-4242.2	-6.8	-0.007
Te-2-TS	-4224.0	11.5	0.030
Te-2-P	-3864.7	-46.4	0.246
Te-2-H^+	-3630.6	0.0	
Te-2-H^+-RC	-4204.7	-12.0	-0.002
Te-2-H^+-TS	-4189.8	2.9	-0.003
Te-2-H^+-P	-3839.2	-63.7	0.207
Te-3	-7997.7	0.0	
Te-3-RC	-8566.7	-7.0	-0.005
Te-3-TS	-8548.2	11.6	0.023
Te-3-P	-8190.2	-47.6	0.253
Te-4	-5206.7	0.0	
Te-4-RC	-5774.4	-5.6	-0.003
Te-4-TS	-5758.8	9.9	0.001
Te-4-P	-5398.5	-46.9	0.254
H_2O	-417.2		
H_2O_2	-562.1		

Table S6. SAPE single point energies.

Gas-phase single-point electronic energies relative to the atomic fragments (in kcal·mol^{-1}) of SAPE geometries reported in Table 4 of the main text (coordinates at Table S9) (level of theory: ZORA-M06/TZ2P-ae//ZORA-OLYP/TZ2P).

	RC	TS	PC
S-1	-4843.1	-4815.5	-4897.5
S-2	-3857.2	-3834.1	-3908.5
S-2-H^+	-3827.0	-3808.0	-3887.0
S-3	-6948.1	-6924.3	-7000.9
S-4	-5386.4	-5367.1	-5441.6
Se-1	-4822.0	-4799.0	-4868.7
Se-2	-3835.7	-3816.9	-3880.0
Se-2-H^+	-3805.0	-3791.4	-3852.3
Se-3	-6904.7	-6883.6	-6951.1
Se-4	-5366.7	-5350.6	-5413.1
Te-1	-4794.8	-4777.7	-4846.8
Te-2	-3808.2	-3796.9	-3861.1
Te-2-H^+	-3778.1	-3770.4	-3840.1
Te-3	-6852.9	-6840.3	-6905.8
Te-4	-5340.4	-5330.0	-5395.5

Table S7. Gas-phase cartesian coordinates.

Cartesian Coordinates (in Å), electronic energies relative to the atomic fragments (in Hartree) and imaginary frequencies (in cm^{-1}) of the compounds investigated (Table 1 in the main text) (level of theory: ZORA-OLYP/TZ2P).

Gas-phase geometries

H_2O

-0.51495479 Nimag=0

O	0.000000000	0.000000000	-0.395601000
H	0.000000000	0.760343000	0.198994000
H	0.000000000	-0.760343000	0.198994000

H_2O_2

-0.65281998 Nimag=0

O	-0.015407000	-0.730641000	-0.278247000
O	0.015407000	0.730641000	-0.278247000
H	0.800760000	0.883499000	0.272116000
H	-0.800760000	-0.883499000	0.272116000

S-1 $C_5NH_{13}S_2O_2$

-4.32884625 Nimag=0

C	-0.340764000	-0.039850000	2.580673000
H	0.657990000	0.107493000	2.158048000
H	-0.726848000	-1.002139000	2.230081000
C	-0.302416000	-0.016582000	4.111180000
H	-1.328926000	-0.137907000	4.476817000
H	-1.005880000	0.735120000	2.193906000
C	0.547819000	-1.191007000	4.632970000
H	0.105616000	-2.129009000	4.282504000
H	1.559600000	-1.130502000	4.217048000
S	0.828498000	-1.297555000	6.426011000
C	-0.818408000	-1.708457000	7.063959000
H	-1.533307000	-0.895073000	6.923269000
H	-1.196433000	-2.628641000	6.609272000
H	-0.687195000	-1.875414000	8.135934000
N	0.276036000	1.238546000	4.634154000
H	0.673821000	1.086161000	5.561955000
S	-0.641568000	2.638884000	4.743585000
O	-2.020331000	2.366690000	4.382954000
O	-0.306657000	3.245906000	6.016851000
C	0.051231000	3.683484000	3.463161000
H	-0.084973000	3.209899000	2.491227000
H	-0.498924000	4.625641000	3.507331000
H	1.107818000	3.844809000	3.677414000

S-2 $C_4NH_{11}S$

-3.15181489 Nimag=0

C	-0.274525000	0.196102000	2.534295000
H	0.687691000	0.024569000	2.041679000
H	-0.946713000	-0.634342000	2.295655000
C	-0.094718000	0.341122000	4.049006000
H	-1.100302000	0.475673000	4.485608000
H	-0.711290000	1.106684000	2.109575000
C	0.503891000	-0.943333000	4.642031000
H	-0.083987000	-1.815079000	4.336391000
H	1.521650000	-1.075014000	4.259044000
S	0.703019000	-0.994903000	6.453739000
C	-0.991117000	-1.310344000	7.017893000
H	-1.667404000	-0.481637000	6.794580000
H	-1.383108000	-2.237441000	6.588609000
H	-0.930523000	-1.426311000	8.103119000
N	0.796959000	1.470602000	4.332533000
H	0.933695000	1.545087000	5.337917000
H	0.363542000	2.339358000	4.032199000

S-2-H$^+$ $C_4NH_{12}S^+$

-3.06097505 Nimag=0

C	-0.162798000	0.241924000	2.404334000
H	0.843808000	0.112844000	1.992903000
H	-0.775847000	-0.588956000	2.045397000
C	-0.158334000	0.235187000	3.924934000
H	-1.169972000	0.402727000	4.305842000
H	-0.595320000	1.163018000	2.002045000
C	0.421190000	-1.043287000	4.558807000
H	-0.247429000	-1.881181000	4.345076000
H	1.391680000	-1.285949000	4.111076000
S	0.742053000	-0.923335000	6.346015000
C	-0.896928000	-1.175251000	7.088787000
H	-1.617203000	-0.404190000	6.809659000
H	-1.273336000	-2.168110000	6.833768000
H	-0.730829000	-1.127214000	8.167756000
N	0.644517000	1.406896000	4.476328000
H	0.832972000	1.151741000	5.479284000
H	0.142610000	2.294084000	4.418260000
H	1.540624000	1.513949000	3.994084000

S-3 $C_{10}H_{22}S_4O_2$

-7.03965056 Nimag=0

C	-0.798284000	1.223680000	1.754979000
H	0.225973000	0.945484000	1.468132000
H	-1.447626000	0.352029000	1.567691000
C	-0.822439000	1.592802000	3.240268000
H	-0.203799000	2.479680000	3.390671000
C	-2.246898000	1.839899000	3.767476000
H	-2.832003000	2.334880000	2.987545000
H	-2.747792000	0.889143000	3.981596000
S	-2.394224000	2.814924000	5.297059000
C	-2.274852000	4.506898000	4.655922000
H	-1.309469000	4.702946000	4.182160000

H	-3.083775000	4.724038000	3.952497000
H	-2.377076000	5.163715000	5.523768000
O	-1.233793000	2.344730000	0.994993000
C	-1.194589000	2.107938000	-0.401042000
H	-0.174487000	1.878836000	-0.747035000
H	-1.856667000	1.277394000	-0.693844000
H	-1.537348000	3.023555000	-0.890123000
S	-0.071590000	0.227561000	4.240240000
S	1.942354000	0.438777000	4.045528000
C	2.512377000	0.769640000	6.741498000
H	1.549771000	0.260431000	6.891971000
H	3.298046000	-0.003254000	6.698324000
C	2.467112000	1.553333000	5.427489000
H	1.712214000	2.337333000	5.513889000
C	3.829690000	2.162873000	5.053535000
H	4.329106000	2.497830000	5.966816000
H	4.478068000	1.404877000	4.599878000
S	3.811846000	3.546900000	3.872052000
C	3.412445000	4.941755000	4.959736000
H	2.427097000	4.837878000	5.421388000
H	4.172922000	5.072522000	5.734918000
H	3.405856000	5.827986000	4.319768000
O	2.760095000	1.676361000	7.809471000
C	2.756089000	1.043227000	9.076608000
H	1.785329000	0.570155000	9.292562000
H	3.542604000	0.275457000	9.152970000
H	2.945520000	1.819784000	9.822339000

S-4 $C_8H_{16}S_2O$

-5.10184333 Nimag=0

S	2.741268000	-0.520221000	0.837306000
C	0.654491000	-1.980671000	-0.095875000
H	1.486547000	-2.517957000	-0.575582000
H	0.468482000	-2.459204000	0.880945000
C	1.050185000	-0.512443000	0.103191000
H	1.136147000	-0.051560000	-0.885168000
C	0.104684000	0.308925000	0.991674000
H	-0.063373000	-0.216351000	1.938486000
H	0.581996000	1.263913000	1.224999000
S	-1.510083000	0.716622000	0.217348000
C	-2.000546000	2.087134000	1.297074000
H	-1.288637000	2.917675000	1.249170000
H	-2.969241000	2.435595000	0.929066000
H	-2.119697000	1.763043000	2.335641000
O	-0.504612000	-2.045195000	-0.908688000
C	-1.013577000	-3.358583000	-1.039338000
H	-1.894580000	-3.295322000	-1.683017000
H	-0.279957000	-4.038199000	-1.502512000
H	-1.312619000	-3.779400000	-0.065976000
C	3.468371000	1.033256000	0.182349000
H	2.842673000	1.886126000	0.461701000
H	4.400659000	1.109496000	0.755014000
C	3.751132000	1.008250000	-1.284608000
H	4.451076000	0.241202000	-1.615718000
C	3.206458000	1.832910000	-2.182150000
H	2.496853000	2.606895000	-1.897285000
H	3.456816000	1.763498000	-3.237216000

Se-1 $C_5NH_{13}SeSO_2$

-4.29447240 Nimag=0

C	-0.348687000	-0.021584000	2.565897000
H	0.639182000	0.139401000	2.123309000
H	-0.733483000	-0.983658000	2.213838000
C	-0.276097000	-0.014981000	4.096897000
H	-1.293688000	-0.149199000	4.482648000
H	-1.029763000	0.751221000	2.203193000
C	0.590812000	-1.186753000	4.580550000
H	0.148060000	-2.126742000	4.243270000
H	1.599525000	-1.118865000	4.160596000
Se	0.922500000	-1.338716000	6.520498000
C	-0.898537000	-1.731842000	7.143799000
H	-1.558771000	-0.876339000	7.001509000
H	-1.287635000	-2.615384000	6.634743000
H	-0.802328000	-1.941227000	8.211015000
N	0.301164000	1.242580000	4.617400000
H	0.701985000	1.090743000	5.544744000
S	-0.622970000	2.638577000	4.734591000
O	-2.005702000	2.359425000	4.394817000
O	-0.272991000	3.251147000	6.001080000
C	0.046572000	3.683094000	3.441520000
H	-0.100507000	3.206605000	2.472644000
H	-0.507967000	4.622422000	3.490989000
H	1.105123000	3.850575000	3.640753000

Se-2 $C_4NH_{11}Se$

-3.11780417 Nimag=0

C	-0.283949000	0.211118000	2.513464000
H	0.666076000	0.041001000	1.997380000
H	-0.960488000	-0.620124000	2.291269000
C	-0.066900000	0.355324000	4.024832000
H	-1.061661000	0.492803000	4.484886000
H	-0.732653000	1.121707000	2.100869000
C	0.539298000	-0.928744000	4.598820000
H	-0.063224000	-1.800408000	4.331555000
H	1.550116000	-1.074109000	4.205537000
Se	0.786704000	-0.985212000	6.560719000
C	-1.066261000	-1.356437000	7.099865000
H	-1.727482000	-0.526858000	6.847227000
H	-1.414905000	-2.281675000	6.636897000

H	-1.042540000	-1.481493000	8.184457000
N	0.832968000	1.484602000	4.283423000
H	0.968049000	1.580213000	5.287526000
H	0.403611000	2.349083000	3.965145000

Se-2-H^{+} C4NH12Se^{+}

-3.02814340 Nimag=0

C	-0.169589000	0.251873000	2.384536000
H	0.830079000	0.145665000	1.950405000
H	-0.775094000	-0.585963000	2.029467000
C	-0.131678000	0.228080000	3.906313000
H	-1.138539000	0.373677000	4.308370000
H	-0.627050000	1.169730000	2.002468000
C	0.477157000	-1.045803000	4.506485000
H	-0.184887000	-1.890340000	4.306388000
H	1.447312000	-1.272526000	4.052122000
Se	0.844641000	-0.953417000	6.446597000
C	-0.971001000	-1.190581000	7.165261000
H	-1.636497000	-0.376308000	6.880568000
H	-1.360196000	-2.156288000	6.841608000
H	-0.842701000	-1.192527000	8.249476000
N	0.656283000	1.416092000	4.444836000
H	0.852348000	1.180948000	5.452693000
H	0.141267000	2.295046000	4.374306000
H	1.549641000	1.529960000	3.959026000

Se-3 $C_{10}H_{22}Se_2S_2O_2$

-6.97186992 Nimag=0

C	-0.814974000	1.162067000	1.738451000
H	0.204919000	0.875671000	1.443262000
H	-1.469642000	0.289910000	1.573978000
C	-0.818689000	1.555779000	3.219030000
H	-0.184646000	2.434929000	3.349752000
C	-2.229705000	1.827506000	3.753204000
H	-2.811890000	2.353482000	2.994344000
H	-2.756606000	0.895422000	3.980007000
Se	-2.345717000	2.903817000	5.404398000
C	-2.308164000	4.688149000	4.581411000
H	-1.378445000	4.846300000	4.033205000
H	-3.169098000	4.823444000	3.924626000
H	-2.366213000	5.398562000	5.408925000
O	-1.256642000	2.271999000	0.966224000
C	-1.245087000	2.009982000	-0.425590000
H	-0.233283000	1.767299000	-0.786590000
H	-1.918206000	1.179057000	-0.690980000
H	-1.590679000	2.919114000	-0.924703000
S	-0.072867000	0.193327000	4.227641000
S	1.942272000	0.405574000	4.045712000
C	2.531090000	0.716757000	6.737983000
H	1.574156000	0.198560000	6.895761000
H	3.321747000	-0.049408000	6.673910000
C	2.460774000	1.516885000	5.433494000
H	1.691601000	2.285197000	5.534475000
C	3.805295000	2.149828000	5.056491000
H	4.301286000	2.515168000	5.957562000
H	4.473436000	1.418774000	4.590661000
Se	3.734024000	3.667143000	3.794493000
C	3.438596000	5.106078000	5.100028000
H	2.503370000	4.956771000	5.641259000
H	4.278286000	5.169355000	5.794154000
H	3.377310000	6.027986000	4.517656000
O	2.787737000	1.612155000	7.813089000
C	2.817520000	0.962357000	9.071171000
H	1.857414000	0.474043000	9.300760000
H	3.614922000	0.203659000	9.121105000
H	3.012601000	1.731301000	9.823346000

Se-4 $C_8H_{16}SeSO$

-5.06926161 Nimag=0

S	2.757068000	-0.553796000	0.830696000
C	0.696676000	-2.018835000	-0.140665000
H	1.499645000	-2.499300000	-0.719390000
H	0.611712000	-2.554624000	0.820385000
C	1.059555000	-0.550424000	0.105428000
H	1.132699000	-0.051423000	-0.865069000
C	0.101611000	0.208898000	1.025113000
H	-0.061461000	-0.345405000	1.953006000
H	0.526921000	1.180592000	1.278185000
Se	-1.672627000	0.632463000	0.218200000
C	-2.014753000	2.257425000	1.271926000
H	-1.255933000	3.017800000	1.074500000
H	-2.989051000	2.628689000	0.945845000
H	-2.057837000	2.028114000	2.338518000
O	-0.528989000	-2.082577000	-0.850836000
C	-1.000922000	-3.405983000	-1.021929000
H	-1.938328000	-3.339791000	-1.579827000
H	-0.287907000	-4.022905000	-1.591846000
H	-1.194454000	-3.898169000	-0.055592000
C	3.460710000	1.021132000	0.201800000
H	2.822089000	1.859056000	0.496583000
H	4.392453000	1.102037000	0.774646000
C	3.742130000	1.025606000	-1.265619000
H	4.452496000	0.274357000	-1.610503000
C	3.185549000	1.858264000	-2.148373000
H	2.465716000	2.617475000	-1.849749000
H	3.436149000	1.810757000	-3.204602000

Te-1 $C_5NH_{13}TeSO_2$

-4.26159715 Nimag=0

C	-0.354361000	-0.000564000	2.544838000

H	0.620007000	0.169110000	2.076440000
H	-0.744263000	-0.961251000	2.195275000
C	-0.237993000	-0.008734000	4.074457000
H	-1.244235000	-0.152081000	4.486126000
H	-1.049481000	0.772066000	2.209080000
C	0.647019000	-1.179685000	4.519309000
H	0.206895000	-2.117436000	4.175279000
H	1.649406000	-1.099374000	4.086959000
Te	1.035175000	-1.420234000	6.652704000
C	-1.013147000	-1.769798000	7.259353000
H	-1.610268000	-0.869415000	7.121254000
H	-1.424674000	-2.608041000	6.696546000
H	-0.972964000	-2.025029000	8.319785000
N	0.342208000	1.252716000	4.585281000
H	0.763419000	1.108038000	5.505057000
S	-0.589157000	2.643093000	4.721622000
O	-1.978922000	2.353252000	4.421956000
O	-0.208066000	3.263291000	5.975202000
C	0.036757000	3.687404000	3.406394000
H	-0.133472000	3.206960000	2.443346000
H	-0.523186000	4.622799000	3.468241000
H	1.099103000	3.863412000	3.575797000

Te-2 $C_4NH_{11}Te$

-3.08459938 Nimag=0

C	-0.926550000	-0.552677000	2.152725000
H	-0.030375000	-1.088762000	1.819594000
H	-1.746396000	-1.276523000	2.222982000
C	-0.684166000	0.155895000	3.498991000
H	-1.612706000	0.668355000	3.781698000
H	-1.191437000	0.175471000	1.378166000
C	-0.359656000	-0.849214000	4.602028000
H	-1.195421000	-1.531916000	4.768841000
H	0.526713000	-1.440454000	4.352313000
Te	0.024784000	0.157160000	6.500775000
C	-0.546508000	-1.550746000	7.714135000
H	-1.592359000	-1.806395000	7.538936000
H	0.101494000	-2.401172000	7.498769000
H	-0.415154000	-1.250615000	8.755833000
N	0.353335000	1.196182000	3.468605000
H	0.107857000	1.921650000	2.800561000
H	1.239439000	0.804549000	3.156111000

Te-2-H$^+$ $C_4NH_{12}Te^+$

-2.99681923 Nimag=0

C	-0.966013000	-0.541894000	2.238200000
H	-0.066652000	-0.648353000	1.622698000
H	-1.507644000	-1.490209000	2.198837000
C	-0.624817000	-0.224769000	3.689497000
H	-1.544452000	-0.088063000	4.266214000
H	-1.612139000	0.222026000	1.794122000
C	0.256709000	-1.271472000	4.374915000
H	-0.308162000	-2.199666000	4.477562000
H	1.148759000	-1.495134000	3.780902000
Te	1.015135000	-0.719779000	6.350981000
C	-0.831841000	-0.989118000	7.445376000
H	-1.596433000	-0.286233000	7.118757000
H	-1.159149000	-2.022514000	7.333382000
H	-0.577032000	-0.794557000	8.488792000
N	0.069899000	1.129996000	3.781654000
H	-0.567922000	1.908339000	3.605980000
H	0.847048000	1.200454000	3.119592000
H	0.461106000	1.190744000	4.757497000

Te-3 $C_{10}H_{22}Te_2S_2O_2$

-6.90809536 Nimag=0

C	-0.779014000	1.136728000	1.729640000
H	0.252041000	0.873125000	1.451970000
H	-1.413846000	0.254518000	1.543609000
C	-0.820035000	1.514991000	3.214394000
H	-0.200211000	2.400594000	3.369857000
C	-2.243396000	1.757193000	3.720385000
H	-2.795912000	2.348375000	2.987699000
H	-2.786651000	0.818469000	3.860868000
Te	-2.434898000	2.855523000	5.594072000
C	-2.765612000	4.784652000	4.664194000
H	-1.912389000	5.047405000	4.038930000
H	-3.686537000	4.764701000	4.081321000
H	-2.860656000	5.506325000	5.477923000
O	-1.225909000	2.246362000	0.960467000
C	-1.176651000	2.001128000	-0.433435000
H	-0.151765000	1.784425000	-0.773700000
H	-1.825245000	1.159144000	-0.723918000
H	-1.530226000	2.908490000	-0.930188000
S	-0.083256000	0.148977000	4.224508000
S	1.935239000	0.348206000	4.054128000
C	2.508469000	0.672298000	6.747986000
H	1.540348000	0.175705000	6.908351000
H	3.283255000	-0.110283000	6.691279000
C	2.457579000	1.463904000	5.436637000
H	1.698401000	2.243943000	5.525301000
C	3.811266000	2.070007000	5.062090000
H	4.272724000	2.509304000	5.948459000
H	4.499028000	1.312991000	4.674970000
Te	3.777951000	3.675745000	3.587293000
C	3.852860000	5.283852000	5.037786000
H	2.992373000	5.224032000	5.704109000
H	4.786378000	5.237113000	5.598662000
H	3.812166000	6.214270000	4.467975000

O	2.781741000	1.571204000	7.815671000
C	2.792541000	0.932483000	9.079532000
H	1.820820000	0.468136000	9.310302000
H	3.572293000	0.156318000	9.139187000
H	3.002539000	1.703646000	9.825389000

Te-4 **$C_8H_{16}TeSO$**

-5.03888139 Nimag=0

S	2.791404000	-0.597579000	0.808319000
C	0.736628000	-2.058423000	-0.172332000
H	1.516551000	-2.513303000	-0.800428000
H	0.702713000	-2.618011000	0.777925000
C	1.087658000	-0.590838000	0.092453000
H	1.156071000	-0.077263000	-0.870607000
C	0.122363000	0.136488000	1.023707000
H	-0.018906000	-0.420600000	1.952042000
H	0.507279000	1.126043000	1.272203000
Te	-1.863823000	0.566922000	0.182554000
C	-2.093490000	2.423692000	1.284243000
H	-1.319650000	3.135704000	0.993817000
H	-3.073525000	2.824440000	1.016261000
H	-2.062264000	2.233552000	2.357695000
O	-0.522104000	-2.123915000	-0.825359000
C	-0.984988000	-3.450168000	-1.007205000
H	-1.948289000	-3.384260000	-1.518888000
H	-0.290966000	-4.040958000	-1.625246000
H	-1.125591000	-3.967564000	-0.045251000
C	3.479986000	0.995655000	0.209738000
H	2.837505000	1.822019000	0.527579000
H	4.415007000	1.070505000	0.778018000
C	3.751708000	1.034737000	-1.258980000
H	4.464494000	0.295959000	-1.625189000
C	3.184443000	1.883559000	-2.119261000
H	2.462059000	2.631494000	-1.799152000
H	3.428643000	1.861547000	-3.177826000

RC-S-1 **$C_5NH_{15}S_2O_4$**

-4.98602341 Nimag=0

C	-0.350431000	0.288821000	2.513892000
H	0.646113000	0.500334000	2.114321000
H	-0.665163000	-0.696796000	2.157471000
C	-0.349865000	0.318152000	4.045125000
H	-1.374493000	0.130241000	4.386601000
H	-1.055292000	1.018747000	2.110413000
C	0.561446000	-0.798143000	4.585457000
H	0.197038000	-1.762868000	4.220980000
H	1.580603000	-0.667647000	4.205624000
S	0.788827000	-0.895041000	6.389425000
C	-0.850684000	-1.397601000	6.981431000
H	-1.595895000	-0.615423000	6.825410000
H	-1.167631000	-2.330535000	6.508724000
H	-0.739240000	-1.564009000	8.055676000
N	0.130101000	1.612215000	4.574179000
H	0.530075000	1.497280000	5.505029000
S	-0.892734000	2.940725000	4.673264000
O	-2.242012000	2.563354000	4.297425000
O	-0.618131000	3.566941000	5.951609000
C	-0.267072000	4.037299000	3.402508000
H	-0.356690000	3.557300000	2.428341000
H	-0.887707000	4.934810000	3.442537000
H	0.771798000	4.277868000	3.628704000
O	2.394645000	-4.008435000	6.064742000
O	1.218367000	-4.850145000	5.848161000
H	1.961967000	-3.159638000	6.284427000
H	1.296445000	-5.446477000	6.609973000

RC-S-2 **$C_4NH_{13}SO_2$**

-3.80914284 Nimag=0

C	-0.074337000	-0.598946000	2.251454000
H	0.958089000	-0.702600000	1.902917000
H	-0.614156000	-1.526276000	2.036081000
C	-0.116685000	-0.269438000	3.747986000
H	-1.180502000	-0.214757000	4.039246000
H	-0.546522000	0.200174000	1.669676000
C	0.534765000	-1.406398000	4.550032000
H	0.137238000	-2.375144000	4.230685000
H	1.613167000	-1.407768000	4.359618000
S	0.408136000	-1.351100000	6.370311000
C	-1.310485000	-1.852302000	6.658477000
H	-2.026278000	-1.126081000	6.267427000
H	-1.502486000	-2.839469000	6.228592000
H	-1.430992000	-1.912300000	7.743024000
N	0.600804000	0.985836000	3.988590000
H	0.524685000	1.258033000	4.964584000
H	0.167737000	1.734995000	3.455601000
O	0.291145000	1.598462000	8.253936000
O	0.612316000	2.673361000	7.318321000
H	0.393261000	0.820626000	7.669218000
H	1.494973000	2.921400000	7.638006000

RC-S-2-H$^+$ **$C_4NH_{14}SO_2$**

-3.73184269 Nimag=0

C	-0.669556000	0.022142000	2.523785000
H	0.273666000	0.102301000	1.972947000
H	-1.236268000	-0.805709000	2.090030000
C	-0.440022000	-0.248418000	4.003818000
H	-1.397846000	-0.285559000	4.529434000
H	-1.250903000	0.934847000	2.363947000
C	0.353317000	-1.535915000	4.290551000
H	-0.239807000	-2.398052000	3.974131000

H	1.278884000	-1.553037000	3.703610000
S	0.889135000	-1.738765000	6.016990000
C	-0.611568000	-2.331709000	6.850735000
H	-1.425901000	-1.605108000	6.830289000
H	-0.931000000	-3.282841000	6.419152000
H	-0.315227000	-2.498744000	7.889136000
N	0.297721000	0.905484000	4.657280000
H	0.650916000	0.532173000	5.564777000
H	-0.304686000	1.728401000	4.825239000
H	1.103263000	1.198373000	4.100275000
O	-2.456481000	2.389614000	6.061394000
O	-1.401534000	3.190574000	5.451866000
H	-2.533663000	2.819514000	6.932290000
H	-1.919622000	3.804457000	4.901657000

RC-S-3 **$C_{10}H_{24}S_4O_4$**

-7.69997505 Nimag=0

C	-0.139890000	0.641422000	2.106363000
H	0.890817000	0.289904000	1.958804000
H	-0.806141000	-0.235805000	2.051797000
C	-0.253458000	1.307314000	3.479771000
H	0.374329000	2.200677000	3.490182000
C	-1.708078000	1.665065000	3.837306000
H	-2.242377000	1.925863000	2.919980000
H	-2.224249000	0.799385000	4.266952000
S	-1.974160000	3.017623000	5.028005000
C	-1.831013000	4.474995000	3.956956000
H	-0.843894000	4.550094000	3.495177000
H	-2.605485000	4.469269000	3.185549000
H	-1.980941000	5.341223000	4.605962000
O	-0.481640000	1.594319000	1.107662000
C	-0.341032000	1.083507000	-0.206792000
H	0.695514000	0.776011000	-0.414984000
H	-1.003282000	0.220978000	-0.383128000
H	-0.616795000	1.888454000	-0.892951000
S	0.374600000	0.164130000	4.795227000
S	2.397278000	0.100849000	4.576532000
C	3.095264000	0.939635000	7.119548000
H	2.119871000	0.507767000	7.382677000
H	3.854672000	0.151063000	7.235826000
C	3.069391000	1.434721000	5.670708000
H	2.391707000	2.285634000	5.593054000
C	4.467178000	1.819679000	5.153191000
H	5.017899000	2.314252000	5.958702000
H	5.037121000	0.922168000	4.887610000
S	4.531426000	2.884812000	3.679886000
C	4.308035000	4.530338000	4.410814000
H	3.341201000	4.636863000	4.907455000
H	5.115937000	4.765749000	5.109616000
H	4.351985000	5.233510000	3.574891000
O	3.390634000	2.036543000	7.988485000
C	3.613586000	1.626683000	9.332153000
H	2.728878000	1.130476000	9.758302000
H	4.471117000	0.941959000	9.404411000
H	3.828618000	2.529288000	9.909468000
O	0.790514000	4.411749000	6.758699000
O	1.061681000	4.096888000	8.158467000
H	0.017721000	3.842914000	6.583702000
H	1.831030000	3.506030000	8.059277000

RC-S-4 **$C_8H_{18}S_2O_3$**

-5.76114994 Nimag=0

S	2.919647000	-0.283543000	1.434117000
C	0.916879000	-1.830517000	0.427794000
H	1.781318000	-2.375037000	0.020086000
H	0.682123000	-2.269330000	1.412951000
C	1.286783000	-0.350931000	0.581654000
H	1.442130000	0.060028000	-0.419799000
C	0.282954000	0.507226000	1.365668000
H	0.068813000	0.037953000	2.331826000
H	0.731702000	1.483767000	1.562080000
S	-1.311645000	0.831901000	0.507186000
C	-1.940167000	2.152785000	1.579381000
H	-1.274990000	3.020630000	1.587157000
H	-2.905221000	2.453460000	1.164084000
H	-2.095192000	1.789443000	2.599263000
O	-0.194095000	-1.946878000	-0.445223000
C	-0.662980000	-3.276586000	-0.565839000
H	-1.507005000	-3.254581000	-1.259799000
H	0.112483000	-3.948958000	-0.966632000
H	-1.006505000	-3.677517000	0.401170000
C	3.744408000	1.125727000	0.588465000
H	3.114592000	2.017381000	0.655955000
H	4.622132000	1.293168000	1.224302000
C	4.155533000	0.838122000	-0.818989000
H	4.875612000	0.029134000	-0.942102000
C	3.698982000	1.484567000	-1.894519000
H	2.970776000	2.288907000	-1.822621000
H	4.044290000	1.225025000	-2.891862000
O	-0.069900000	2.905984000	-1.918155000
O	-0.780386000	4.157699000	-1.664486000
H	-0.518143000	2.308984000	-1.279316000
H	-1.313372000	4.217414000	-2.473501000

RC-Se-1 **$C_5NH_{15}SeSO_4$**

-4.95162886 Nimag=0

C	0.310530000	0.180566000	2.347613000
H	1.395533000	0.220936000	2.211437000
H	-0.059259000	-0.753335000	1.913749000

C	-0.063721000	0.254163000	3.832539000
H	-1.157541000	0.242312000	3.904216000
H	-0.147234000	1.000037000	1.789399000
C	0.490296000	-0.974674000	4.564730000
H	0.077109000	-1.881960000	4.120050000
H	1.580211000	-1.022339000	4.476476000
Se	0.194433000	-1.075808000	6.516617000
C	-1.763325000	-1.237040000	6.560045000
H	-2.235668000	-0.311845000	6.230951000
H	-2.083667000	-2.082673000	5.950008000
H	-2.017592000	-1.423523000	7.605198000
N	0.472122000	1.473847000	4.475125000
H	0.590977000	1.331296000	5.478237000
S	-0.311959000	2.951281000	4.316287000
O	-1.573809000	2.779493000	3.621620000
O	-0.254846000	3.578564000	5.621965000
C	0.774241000	3.879884000	3.235732000
H	0.846367000	3.379425000	2.270492000
H	0.317583000	4.864905000	3.119441000
H	1.752128000	3.962968000	3.710141000
O	0.551038000	-4.683647000	6.444076000
O	1.432280000	-4.886405000	5.295685000
H	0.590889000	-3.709512000	6.526700000
H	2.189025000	-5.306201000	5.735493000

RC-Se-2 **$C_4NH_{13}SeO_2$**

-3.7750862 Nimag=0

C	-0.063799000	-0.341975000	2.205083000
H	0.970643000	-0.432619000	1.859039000
H	-0.561204000	-1.309499000	2.087297000
C	-0.114183000	0.131391000	3.663685000
H	-1.178339000	0.171621000	3.956357000
H	-0.575678000	0.374143000	1.552746000
C	0.585861000	-0.891457000	4.563679000
H	0.221183000	-1.902682000	4.367706000
H	1.664581000	-0.871691000	4.382676000
Se	0.434181000	-0.645466000	6.523488000
C	-1.369455000	-1.377170000	6.793876000
H	-2.119480000	-0.776821000	6.278737000
H	-1.402595000	-2.414211000	6.455495000
H	-1.551207000	-1.339765000	7.869864000
N	0.551034000	1.433533000	3.771531000
H	0.435409000	1.812268000	4.707416000
H	0.100922000	2.098419000	3.148371000
O	-0.631442000	2.582645000	7.724408000
O	-0.106640000	3.556959000	6.770793000
H	0.594381000	3.961997000	7.306676000
H	-0.279533000	1.752361000	7.341029000

RC-Se-2-H$^+$ **$C_4NH_{14}SeO_2^+$**

-3.69877162 Nimag=0

C	-0.332326000	0.181360000	3.831173000
H	0.684721000	0.240647000	4.233146000
H	-0.269432000	-0.274533000	2.839906000
C	-1.241198000	-0.663454000	4.715289000
H	-2.253966000	-0.673809000	4.304172000
H	-0.721242000	1.195746000	3.702982000
C	-0.754813000	-2.104783000	4.911326000
H	-0.807878000	-2.636712000	3.959460000
H	0.288395000	-2.128773000	5.243582000
Se	-1.732000000	-3.140289000	6.281187000
C	-3.392687000	-3.533542000	5.303957000
H	-3.958476000	-2.630096000	5.078442000
H	-3.154497000	-4.091935000	4.398106000
H	-3.972251000	-4.165439000	5.979759000
N	-1.403477000	-0.021309000	6.081111000
H	-0.500815000	0.257500000	6.471445000
H	-1.807750000	-0.763955000	6.695647000
H	-2.025316000	0.804229000	6.066271000
O	-3.294778000	2.271705000	5.964756000
O	-4.416919000	1.510546000	5.427458000
H	-3.696928000	2.671466000	6.756491000
H	-4.746332000	2.124388000	4.746429000

RC-Se-3 **$C_{10}H_{24}Se_2S_2O_4$**

-7.63210381 Nimag=0

C	-0.130425000	0.603394000	2.105174000
H	0.906981000	0.263422000	1.979237000
H	-0.786169000	-0.280786000	2.039239000
C	-0.279510000	1.270551000	3.476262000
H	0.340776000	2.169124000	3.498203000
C	-1.741530000	1.610062000	3.792202000
H	-2.239503000	1.937115000	2.877702000
H	-2.282794000	0.733341000	4.161546000
Se	-2.090571000	3.018642000	5.133107000
C	-1.902243000	4.585744000	3.961682000
H	-0.901561000	4.632212000	3.531262000
H	-2.659647000	4.559550000	3.177014000
H	-2.063096000	5.456281000	4.600571000
O	-0.462111000	1.550675000	1.097910000
C	-0.293106000	1.037365000	-0.211987000
H	0.749509000	0.737458000	-0.400373000
H	-0.945094000	0.168998000	-0.397915000
H	-0.562606000	1.838060000	-0.905598000
S	0.331514000	0.132994000	4.805091000
S	2.355076000	0.056192000	4.601614000
C	3.072671000	0.916051000	7.131478000
H	2.094494000	0.499970000	7.409996000
H	3.822683000	0.118166000	7.243236000

C	3.035371000	1.401147000	5.678247000
H	2.356555000	2.251324000	5.598516000
C	4.424860000	1.777312000	5.149547000
H	4.974221000	2.318601000	5.922498000
H	5.008158000	0.885456000	4.899964000
Se	4.470761000	2.903390000	3.528190000
C	4.404149000	4.666105000	4.395513000
H	3.482042000	4.789468000	4.963706000
H	5.276930000	4.807102000	5.035304000
H	4.430262000	5.394019000	3.581587000
O	3.394972000	2.015257000	7.987564000
C	3.650905000	1.611594000	9.327124000
H	2.775107000	1.123095000	9.779756000
H	4.506167000	0.922295000	9.380433000
H	3.886427000	2.516265000	9.893127000
O	0.789100000	4.426554000	6.829395000
O	1.062364000	4.061469000	8.216653000
H	0.018548000	3.859791000	6.632312000
H	1.834932000	3.479172000	8.095224000

RC-Se-4 $C_8H_{18}SeSO_3$

-5.72859428 Nimag=0

S	3.217269000	-0.750458000	0.985778000
C	0.897963000	-1.979267000	0.261122000
H	1.588167000	-2.624784000	-0.301391000
H	0.780069000	-2.414203000	1.268434000
C	1.491971000	-0.570687000	0.357190000
H	1.572752000	-0.170951000	-0.657320000
C	0.731409000	0.401189000	1.260934000
H	0.595524000	-0.025744000	2.257811000
H	1.294037000	1.330235000	1.357722000
Se	-1.066632000	0.962380000	0.596131000
C	-1.179709000	2.615550000	1.655781000
H	-0.373590000	3.302402000	1.392994000
H	-2.139338000	3.071732000	1.403973000
H	-1.158193000	2.383046000	2.721930000
O	-0.357096000	-1.907062000	-0.396864000
C	-1.033674000	-3.150149000	-0.438883000
H	-1.975321000	-2.982537000	-0.967602000
H	-0.450906000	-3.912837000	-0.979162000
H	-1.253928000	-3.528834000	0.571718000
C	4.118171000	0.531427000	0.023349000
H	3.638170000	1.503981000	0.164830000
H	5.087425000	0.564734000	0.535266000
C	4.292661000	0.199937000	-1.423080000
H	4.866800000	-0.702714000	-1.632172000
C	3.793888000	0.913712000	-2.435383000
H	3.203943000	1.813384000	-2.276938000
H	3.962091000	0.615687000	-3.466940000
O	0.208141000	2.735008000	-2.191223000
O	-0.459608000	4.031988000	-2.106703000
H	-0.244302000	2.247490000	-1.467186000
H	-1.019349000	3.987186000	-2.898517000

RC-Te-1 $C_5NH_{15}TeSO_4$

-4.91841275 Nimag=0

C	0.832211000	0.154027000	1.927758000
H	1.855676000	-0.227399000	2.017578000
H	0.260110000	-0.562216000	1.329685000
C	0.185917000	0.344274000	3.310868000
H	-0.824713000	0.731941000	3.164905000
H	0.861046000	1.102878000	1.387877000
C	0.116187000	-0.990733000	4.062476000
H	-0.301370000	-1.771678000	3.423361000
H	1.110785000	-1.322996000	4.371072000
Te	-1.097497000	-1.034384000	5.873610000
C	-2.854055000	-1.894362000	4.942141000
H	-3.246262000	-1.212720000	4.187441000
H	-2.604855000	-2.862039000	4.507000000
H	-3.592078000	-2.025623000	5.735643000
N	0.898541000	1.340718000	4.132448000
H	1.906722000	1.220800000	4.138769000
S	0.586075000	2.982447000	4.015921000
O	1.876840000	3.642329000	3.990345000
O	-0.406291000	3.215562000	2.985546000
C	-0.181795000	3.370087000	5.586095000
H	0.524353000	3.152184000	6.387652000
H	-0.413572000	4.436716000	5.557281000
H	-1.091673000	2.780417000	5.695287000
O	0.951874000	-4.173041000	6.645868000
O	0.799364000	-4.929065000	5.403449000
H	0.392564000	-5.739435000	5.749504000
H	0.303111000	-3.454563000	6.497357000

RC-Te-2 $C_4NH_{13}TeO_2$

-3.74405801 Nimag=0

C	-0.799570000	-0.399695000	2.207481000
H	-0.000673000	-1.106945000	1.958438000
H	-1.754975000	-0.933820000	2.152803000
C	-0.579586000	0.218667000	3.600746000
H	-1.404016000	0.917781000	3.792824000
H	-0.811504000	0.381690000	1.439773000
C	-0.620082000	-0.852373000	4.689162000
H	-1.617877000	-1.285422000	4.783713000
H	0.094051000	-1.655920000	4.489882000
Te	-0.101687000	0.011752000	6.624751000
C	-0.968218000	-1.618821000	7.768433000
H	-2.044822000	-1.663019000	7.599750000
H	-0.493740000	-2.561449000	7.494014000

H	-0.771367000	-1.404845000	8.820786000
N	0.653199000	1.003978000	3.741938000
H	0.668637000	1.766949000	3.071178000
H	1.471769000	0.428902000	3.554756000
O	3.666593000	-1.068237000	4.794723000
O	3.210036000	-1.657734000	6.051297000
H	2.425010000	-1.099185000	6.246867000
H	4.447444000	-0.583780000	5.107090000

RC-Te-2-H$^+$ **$C_4NH_{14}TeO_2^+$**

-3.66181874 Nimag=0

C	1.086691000	-3.895441000	6.115897000
H	1.729430000	-4.244755000	6.930565000
H	0.091151000	-4.315640000	6.275296000
C	0.991005000	-2.370464000	6.077649000
H	0.362806000	-2.064796000	5.235916000
H	1.462314000	-4.301233000	5.170452000
C	0.464401000	-1.772292000	7.381156000
H	-0.466937000	-2.282176000	7.634732000
H	1.156183000	-1.957268000	8.210021000
Te	-0.011114000	0.362964000	7.483795000
C	-1.903332000	0.237574000	6.443613000
H	-1.765575000	-0.079271000	5.410806000
H	-2.563499000	-0.437978000	6.987341000
H	-2.315108000	1.248300000	6.465498000
N	2.364471000	-1.802356000	5.743140000
H	2.741491000	-2.257554000	4.909247000
H	3.025057000	-1.972238000	6.505052000
H	2.351318000	-0.769362000	5.542297000
O	2.543170000	0.874174000	4.903593000
O	2.246918000	0.656156000	3.490105000
H	2.816739000	1.332285000	3.082523000
H	1.721057000	1.299019000	5.224240000

RC-Te-3 **$C_{10}H_{24}Te_2S_2O_4$**

-7.56712921 Nimag=0

C	-0.706554000	1.728192000	1.328553000
H	0.341738000	1.534520000	1.056844000
H	-1.296079000	0.840526000	1.044876000
C	-0.798772000	1.959515000	2.841725000
H	-0.219503000	2.847708000	3.103139000
C	-2.239852000	2.113342000	3.319098000
H	-2.720420000	2.904522000	2.741075000
H	-2.808378000	1.189951000	3.183170000
Te	-2.445461000	2.722274000	5.409702000
C	-4.157776000	4.001364000	5.053129000
H	-3.893948000	4.785881000	4.343798000
H	-4.995296000	3.406516000	4.688660000
H	-4.414784000	4.447052000	6.015882000
O	-1.183026000	2.884773000	0.656361000
C	-1.080274000	2.780210000	-0.752985000
H	-0.036610000	2.642565000	-1.076512000
H	-1.680252000	1.943614000	-1.145261000
H	-1.460373000	3.716117000	-1.170302000
S	-0.039813000	0.520617000	3.731920000
S	1.978696000	0.771029000	3.575487000
C	2.559418000	0.619996000	6.281489000
H	1.584525000	0.116414000	6.361487000
H	3.319587000	-0.151627000	6.074611000
C	2.508318000	1.632085000	5.130420000
H	1.755561000	2.389916000	5.357570000
C	3.860685000	2.292472000	4.877928000
H	4.227232000	2.712250000	5.816226000
H	4.595946000	1.573960000	4.506805000
Te	3.797066000	3.972455000	3.479441000
C	5.394822000	5.066779000	4.450280000
H	5.121355000	5.290601000	5.481439000
H	6.318612000	4.489847000	4.404903000
H	5.515103000	5.996359000	3.890777000
O	2.857150000	1.310386000	7.485828000
C	2.860013000	0.460727000	8.619510000
H	1.879582000	-0.016666000	8.773484000
H	3.623546000	-0.329107000	8.536292000
H	3.089357000	1.086755000	9.485560000
O	-0.172773000	5.901746000	5.005253000
O	1.055270000	5.756358000	5.781590000
H	1.668688000	5.438112000	5.091900000
H	-0.650784000	5.089966000	5.264833000

RC-Te-4 **$C_8H_{18}TeSO_3$**

-5.69769984 Nimag=0

S	2.582826000	-1.089073000	0.846670000
C	0.391504000	-2.281108000	-0.236325000
H	1.122652000	-2.751498000	-0.909181000
H	0.303838000	-2.916986000	0.661033000
C	0.885193000	-0.884026000	0.148691000
H	0.997840000	-0.300117000	-0.768580000
C	0.008908000	-0.145710000	1.156839000
H	-0.144078000	-0.745721000	2.056269000
H	0.477191000	0.796064000	1.444458000
Te	-1.980554000	0.468525000	0.442230000
C	-2.120587000	2.130311000	1.832178000
H	-1.309992000	2.838913000	1.659123000
H	-3.078759000	2.617924000	1.641165000
H	-2.099485000	1.759187000	2.857337000
O	-0.868731000	-2.164493000	-0.881604000
C	-1.452210000	-3.417416000	-1.193151000
H	-2.404886000	-3.212100000	-1.687491000
H	-0.814764000	-4.002568000	-1.873846000

H	-1.640472000	-4.014967000	-0.287518000
C	3.473729000	0.356545000	0.140497000
H	2.935380000	1.276007000	0.387739000
H	4.407082000	0.355971000	0.716241000
C	3.751893000	0.248195000	-1.323498000
H	4.382884000	-0.589475000	-1.620731000
C	3.278375000	1.080184000	-2.254150000
H	2.634253000	1.921241000	-2.009780000
H	3.521069000	0.943410000	-3.304567000
O	-0.543854000	3.015509000	-1.911891000
O	-0.306287000	2.494290000	-3.255791000
H	-0.963206000	2.239053000	-1.480207000
H	-1.007498000	2.954325000	-3.744667000

PC-S-1 $C_5NH_{15}S_2O_4$

-5.06225902 Nimag=0

C	0.115654000	0.002174000	2.395765000
H	1.165232000	0.010097000	2.085403000
H	-0.314188000	-0.964962000	2.118152000
C	-0.020338000	0.243336000	3.909030000
H	-1.083543000	0.233945000	4.159961000
H	-0.409268000	0.781827000	1.837477000
C	0.690390000	-0.874922000	4.683075000
H	0.342466000	-1.857096000	4.352536000
H	1.772095000	-0.839057000	4.507902000
S	0.601306000	-0.883770000	6.535006000
C	-1.146908000	-1.355855000	6.727280000
H	-1.779065000	-0.528936000	6.398766000
H	-1.354141000	-2.277943000	6.179906000
H	-1.286859000	-1.515324000	7.798731000
N	0.477616000	1.577335000	4.276994000
H	1.449034000	1.657287000	4.548214000
S	-0.429165000	2.764149000	4.994924000
O	-1.650455000	2.179496000	5.514507000
O	0.480691000	3.509702000	5.838035000
C	-0.910549000	3.842765000	3.646323000
H	-1.515129000	3.281154000	2.933179000
H	-1.501334000	4.647388000	4.089033000
H	-0.012509000	4.242306000	3.174439000
O	1.431482000	-2.069790000	6.946611000
O	0.810920000	-4.573283000	5.473015000
H	1.120856000	-3.856597000	6.055518000
H	1.202172000	-5.371783000	5.845221000

PC-S-2 $C_4NH_{13}SO_2$

-3.88738807 Nimag=0

C	-0.391338000	-0.464886000	2.404462000
H	0.433812000	-0.876814000	1.815410000
H	-1.213278000	-1.187406000	2.417714000
C	0.066213000	-0.119467000	3.826535000
H	-0.819720000	0.243305000	4.373625000
H	-0.751064000	0.435821000	1.894970000
C	0.595601000	-1.377100000	4.532829000
H	-0.010993000	-2.261730000	4.313000000
H	1.620763000	-1.575135000	4.203701000
S	0.715156000	-1.352431000	6.376750000
C	-1.059004000	-1.493192000	6.780201000
H	-1.630761000	-0.667839000	6.353038000
H	-1.425566000	-2.459047000	6.423639000
H	-1.116223000	-1.457281000	7.870047000
N	1.137158000	0.881899000	3.765353000
H	1.398554000	1.154498000	4.708851000
H	0.787312000	1.721106000	3.310472000
O	1.161302000	0.015213000	6.823509000
O	-0.636462000	2.356733000	7.435761000
H	0.009379000	1.642817000	7.302072000
H	-0.239917000	2.908198000	8.120121000

PC-S-2-H⁺ $C_4NH_{14}SO_2^+$

-3.81440351 Nimag=0

C	-0.428568000	0.617472000	2.968307000
H	0.523205000	1.144607000	2.858202000
H	-0.434941000	-0.220525000	2.266758000
C	-0.631577000	0.102034000	4.388677000
H	-1.588303000	-0.425270000	4.445849000
H	-1.240285000	1.292384000	2.677658000
C	0.495180000	-0.821593000	4.864441000
H	0.597719000	-1.674779000	4.189332000
H	1.454772000	-0.294825000	4.882028000
S	0.308770000	-1.497697000	6.583557000
C	-0.952790000	-2.771439000	6.304196000
H	-1.859576000	-2.364500000	5.853265000
H	-0.513930000	-3.559656000	5.686350000
H	-1.178706000	-3.170861000	7.295819000
N	-0.760923000	1.245638000	5.377038000
H	-0.744058000	0.790410000	6.342790000
H	-1.631099000	1.758314000	5.235676000
H	0.023238000	1.919377000	5.318414000
O	-0.351907000	-0.380325000	7.377136000
O	1.473435000	3.122614000	5.453241000
H	1.900577000	3.325363000	6.296737000
H	1.678781000	3.880104000	4.888949000

PC-S-3 $C_{10}H_{24}S_4O_4$

-7.77834613 Nimag=0

C	-0.291308000	2.716600000	1.734396000
H	0.680054000	2.524096000	1.256141000
H	-1.001520000	1.955401000	1.369543000
C	-0.120693000	2.599101000	3.252395000
H	0.586231000	3.362440000	3.585443000

C	-1.439694000	2.774164000	4.002708000
H	-1.948676000	3.667339000	3.629964000
H	-2.101059000	1.907536000	3.886143000
S	-1.185973000	3.002459000	5.819531000
C	-2.920175000	3.412974000	6.207659000
H	-3.242114000	4.289522000	5.641596000
H	-3.546139000	2.543273000	5.990520000
H	-2.946829000	3.626360000	7.278339000
O	-0.749452000	4.022599000	1.418422000
C	-0.850215000	4.246548000	0.022668000
H	0.123794000	4.134417000	-0.478418000
H	-1.568326000	3.558284000	-0.450948000
H	-1.202036000	5.272740000	-0.109961000
S	0.617879000	0.950073000	3.674509000
S	2.622171000	1.186321000	3.415477000
C	3.455482000	0.622027000	5.998782000
H	2.522803000	0.041213000	6.049588000
H	4.246905000	-0.049767000	5.625709000
C	3.264815000	1.800591000	5.039286000
H	2.507534000	2.469613000	5.451218000
C	4.569773000	2.574584000	4.783596000
H	5.121282000	2.645156000	5.725464000
H	5.211032000	2.027915000	4.082919000
S	4.414803000	4.243805000	4.078940000
C	3.920604000	5.212204000	5.531040000
H	2.935349000	4.926265000	5.906111000
H	4.664713000	5.136896000	6.329072000
H	3.874281000	6.250210000	5.191153000
O	3.790659000	1.125639000	7.285123000
C	3.938153000	0.102844000	8.253593000
H	3.008860000	-0.474522000	8.381502000
H	4.748788000	-0.595631000	7.991176000
H	4.184077000	0.592277000	9.199534000
O	-0.370741000	4.253960000	5.999622000
O	-1.653482000	6.913585000	5.391292000
H	-1.092619000	7.436221000	4.806541000
H	-1.120794000	6.124367000	5.589184000

PC-S-4 **$C_8H_{186}S_2O_3$**

-5.84377659 Nimag=0

S	2.824491000	0.025637000	1.240494000
C	0.967385000	-1.761375000	0.431230000
H	1.773169000	-2.255713000	-0.130473000
H	1.013833000	-2.133019000	1.470160000
C	1.205562000	-0.247412000	0.406400000
H	1.333064000	0.074965000	-0.630545000
C	0.107726000	0.586157000	1.080693000
H	-0.293025000	0.079425000	1.964497000
H	0.497731000	1.561635000	1.378984000
S	-1.336433000	0.968937000	-0.023922000
C	-2.252848000	1.950948000	1.214693000
H	-1.633088000	2.767776000	1.591858000
H	-3.126413000	2.349929000	0.694583000
H	-2.576747000	1.291720000	2.024476000
O	-0.297306000	-2.061460000	-0.137208000
C	-0.568668000	-3.451114000	-0.166936000
H	-1.555990000	-3.573315000	-0.619567000
H	0.171921000	-3.998053000	-0.771297000
H	-0.582053000	-3.886651000	0.845122000
C	3.590635000	1.349396000	0.218922000
H	2.928543000	2.217314000	0.176793000
H	4.461475000	1.624313000	0.826717000
C	4.014813000	0.898261000	-1.140944000
H	4.750135000	0.093789000	-1.164807000
C	3.552883000	1.404983000	-2.286903000
H	2.816019000	2.204094000	-2.305400000
H	3.907752000	1.036914000	-3.246055000
O	-0.836637000	1.933342000	-1.067708000
O	0.823213000	4.271200000	-0.459715000
H	0.228329000	3.551919000	-0.743218000
H	0.588645000	5.013404000	-1.028487000

PC-Se-1 **$C_5NH_{15}SeSO_4$**

-5.01247205 Nimag=0

C	0.789105000	-0.061141000	2.247784000
H	1.877106000	-0.149759000	2.165294000
H	0.346547000	-0.997092000	1.894505000
C	0.361637000	0.226699000	3.698264000
H	-0.726972000	0.318223000	3.719488000
H	0.461787000	0.745209000	1.585777000
C	0.787619000	-0.925077000	4.612731000
H	0.431707000	-1.887036000	4.239396000
H	1.877504000	-0.984466000	4.703157000
Se	0.217575000	-0.890978000	6.546775000
C	-1.694011000	-1.311945000	6.234845000
H	-2.174559000	-0.450034000	5.770256000
H	-1.758099000	-2.211983000	5.623480000
H	-2.112011000	-1.490629000	7.226832000
N	0.885018000	1.523280000	4.149493000
H	1.844861000	1.566770000	4.466003000
S	-0.013731000	2.774305000	4.745728000
O	-1.320467000	2.270158000	5.121758000
O	0.838413000	3.468994000	5.687054000
C	-0.268210000	3.872152000	3.351038000
H	-0.821719000	3.345158000	2.572893000
H	-0.852567000	4.716525000	3.722513000
H	0.701799000	4.210574000	2.985526000
O	0.866594000	-2.321833000	7.105482000

O	0.236607000	-4.536933000	5.344972000
H	0.506436000	-3.892282000	6.030047000
H	0.424393000	-5.397330000	5.736491000

PC-Se-2 **$C_4NH_{13}SeO_2$**

-3.83700171 Nimag=0

C	-0.460359000	-0.623609000	2.216488000
H	0.477140000	-0.683176000	1.655226000
H	-0.822673000	-1.637990000	2.409765000
C	-0.270346000	0.156838000	3.523942000
H	-1.239355000	0.143731000	4.052749000
H	-1.201169000	-0.122704000	1.583230000
C	0.767079000	-0.543106000	4.405655000
H	0.603587000	-1.623150000	4.467851000
H	1.777097000	-0.352379000	4.032004000
Se	0.844384000	0.038924000	6.330623000
C	-0.760993000	-0.953363000	6.937167000
H	-1.609660000	-0.678343000	6.311260000
H	-0.549599000	-2.023820000	6.884752000
H	-0.929618000	-0.637959000	7.967120000
N	0.190907000	1.516273000	3.226851000
H	0.219142000	2.052283000	4.091688000
H	-0.480409000	1.976172000	2.617736000
O	0.358419000	1.637753000	6.348223000
O	-1.253830000	2.221697000	8.777421000
H	-0.782364000	2.845690000	9.341111000
H	-0.704478000	2.163668000	7.974565000

PC-Se-2-H$^+$ **$C_4NH_{14}SeO_2^+$**

-3.76650978 Nimag=0

C	-0.031664000	-0.858851000	2.603668000
H	0.440790000	-1.826126000	2.805587000
H	-0.939787000	-1.037696000	2.019035000
C	-0.335621000	-0.099475000	3.903906000
H	-0.799408000	0.857211000	3.641617000
H	0.650193000	-0.269617000	1.984167000
C	-1.350939000	-0.876585000	4.747136000
H	-2.350332000	-0.918813000	4.309419000
H	-1.012925000	-1.894025000	4.982094000
Se	-1.507183000	-0.044495000	6.525850000
C	-3.154158000	-0.933137000	7.089684000
H	-3.931486000	-0.752160000	6.347332000
H	-2.932639000	-1.998935000	7.189472000
H	-3.421206000	-0.509031000	8.058655000
N	0.817477000	0.217772000	4.754530000
H	1.438290000	-0.584478000	4.844381000
H	-1.739332000	4.097184000	7.923598000
H	1.373902000	0.957926000	4.336167000
O	-2.216768000	1.496716000	6.036953000
O	-1.122846000	3.471609000	7.519166000
H	-1.768621000	2.221836000	6.560957000
H	-0.383367000	4.011931000	7.210055000

PC-Se-3 **$C_{10}H_{24}Se_2S_2O_4$**

-7.69188162 Nimag=0

C	-0.087243000	0.716518000	2.184462000
H	0.943568000	0.414629000	1.952317000
H	-0.732116000	-0.171877000	2.082031000
C	-0.132909000	1.248591000	3.618428000
H	0.523406000	2.119965000	3.703612000
C	-1.539818000	1.617914000	4.081276000
H	-2.223675000	1.739388000	3.240037000
H	-1.967531000	0.899372000	4.785953000
Se	-1.592551000	3.388750000	5.091569000
C	-2.180792000	4.478406000	3.544594000
H	-1.490220000	4.305107000	2.719592000
H	-3.203237000	4.196797000	3.281089000
H	-2.141173000	5.515307000	3.881297000
O	-0.511700000	1.745299000	1.297983000
C	-0.417406000	1.365691000	-0.064829000
H	0.618896000	1.127326000	-0.349074000
H	-1.051410000	0.493621000	-0.290823000
H	-0.760692000	2.217228000	-0.658127000
S	0.521178000	-0.018731000	4.804762000
S	2.540904000	-0.023801000	4.576551000
C	3.163061000	0.814360000	7.147497000
H	2.164810000	0.421858000	7.388567000
H	3.888188000	-0.005030000	7.279864000
C	3.181996000	1.305602000	5.696129000
H	2.505486000	2.155352000	5.597538000
C	4.591025000	1.684626000	5.225535000
H	5.095245000	2.239514000	6.019292000
H	5.193462000	0.794093000	5.018691000
Se	4.724984000	2.777434000	3.587628000
C	4.344835000	4.539003000	4.370910000
H	3.325049000	4.589785000	4.752186000
H	5.066077000	4.768256000	5.157064000
H	4.461134000	5.252875000	3.552465000
O	3.481121000	1.901926000	8.011658000
C	3.538185000	1.515808000	9.376153000
H	2.572259000	1.122538000	9.728293000
H	4.312461000	0.752529000	9.548667000
H	3.786668000	2.411155000	9.951427000
O	-0.000446000	3.835264000	5.276710000
O	1.221812000	4.514495000	8.002863000
H	0.786685000	4.353187000	7.149750000
H	1.912609000	3.840735000	8.030745000

PC-Se-4 **$C_8H_{18}SeSO_3$**

-5.79470638 Nimag=0

S	3.139687000	-0.437226000	0.753000000
C	0.934946000	-1.938719000	0.296533000
H	1.560706000	-2.550658000	-0.368594000
H	1.083697000	-2.309910000	1.325590000
C	1.382749000	-0.477749000	0.192991000
H	1.388487000	-0.182698000	-0.859952000
C	0.552952000	0.509586000	1.009969000
H	0.305506000	0.120441000	2.002650000
H	1.069650000	1.463779000	1.113313000
Se	-1.206157000	1.032302000	0.154802000
C	-1.619760000	2.384548000	1.550860000
H	-0.794988000	3.095433000	1.606776000
H	-2.535488000	2.881039000	1.225897000
H	-1.779951000	1.875387000	2.504388000
O	-0.434112000	-2.046123000	-0.066336000
C	-0.908722000	-3.380797000	-0.035168000
H	-1.960604000	-3.352596000	-0.330657000
H	-0.357086000	-4.024305000	-0.737886000
H	-0.831038000	-3.817911000	0.973040000
C	3.912975000	0.739497000	-0.430757000
H	3.373713000	1.689449000	-0.413642000
H	4.895843000	0.904957000	0.027357000
C	4.057489000	0.199382000	-1.816533000
H	4.666032000	-0.699980000	-1.913157000
C	3.495972000	0.737464000	-2.902183000
H	2.878478000	1.630403000	-2.846382000
H	3.646020000	0.299642000	-3.885684000
O	-0.754402000	1.943135000	-1.168651000
O	1.338152000	3.874155000	-0.889432000
H	0.587362000	3.264079000	-1.048947000
H	1.127722000	4.654031000	-1.415342000

PC-Te-1 **$C_5NH_{15}TeSO_4$**

-4.98734396 Nimag=0

C	-0.092681000	-0.438575000	2.388038000
H	0.921623000	-0.166579000	2.081746000
H	-0.266034000	-1.490447000	2.138407000
C	-0.280703000	-0.212499000	3.895577000
H	-1.299659000	-0.524377000	4.161584000
H	-0.799331000	0.169918000	1.823157000
C	0.708420000	-1.090093000	4.667826000
H	0.528464000	-2.147921000	4.453166000
H	1.742594000	-0.858539000	4.391151000
Te	0.783913000	-0.928472000	6.854522000
C	-1.341566000	-1.083129000	7.223726000
H	-1.831644000	-0.218588000	6.777483000
H	-1.712174000	-2.021574000	6.804793000
H	-1.465966000	-1.080011000	8.308031000
N	-0.066198000	1.215335000	4.256512000
H	0.323680000	1.296360000	5.205848000
S	-1.413377000	2.221435000	4.190808000
O	-1.933076000	2.193315000	2.837804000
O	-2.335349000	1.964231000	5.287807000
C	-0.621230000	3.796401000	4.495082000
H	0.090046000	3.998363000	3.694792000
H	-1.426467000	4.533692000	4.497682000
H	-0.131335000	3.771638000	5.469572000
O	1.061743000	0.889081000	7.026059000
O	3.924923000	1.441333000	6.633782000
H	4.288533000	1.970902000	7.352613000
H	2.975686000	1.365126000	6.846092000

PC-Te-2 **$C_4NH_{13}TeO_2$**

-3.81772561 Nimag=0

C	-0.614387000	-0.555491000	2.246491000
H	0.234647000	-1.223662000	2.069965000
H	-1.536149000	-1.140556000	2.150471000
C	-0.512162000	0.106381000	3.632156000
H	-1.381779000	0.768463000	3.748689000
H	-0.618812000	0.205526000	1.458540000
C	-0.562426000	-0.928403000	4.757154000
H	-1.551946000	-1.375700000	4.880750000
H	0.178199000	-1.723610000	4.626362000
Te	0.045863000	0.017643000	6.635578000
C	-0.916619000	-1.527116000	7.831888000
H	-2.002267000	-1.468304000	7.719329000
H	-0.537173000	-2.494485000	7.498992000
H	-0.634602000	-1.351521000	8.871485000
N	0.669522000	0.950132000	3.839780000
H	0.680793000	1.730372000	3.190151000
H	1.531706000	0.423673000	3.703815000
O	3.189796000	-1.097507000	4.443907000
O	1.793628000	-0.525098000	6.817825000
H	2.793861000	-0.919660000	5.327954000
H	4.137731000	-0.990203000	4.579927000

PC-Te-2-H$^+$ **$C_4NH_{14}TeO_2^+$**

-3.75421600 Nimag=0

C	-2.870484000	-0.849555000	3.616914000
H	-2.439746000	-1.642617000	2.996140000
H	-3.691204000	-1.282646000	4.196870000
C	-1.816752000	-0.223637000	4.535668000
H	-2.291531000	0.571419000	5.119229000
H	-3.296072000	-0.088947000	2.956053000
C	-1.238931000	-1.235461000	5.531859000
H	-1.890964000	-1.461359000	6.377901000
H	-0.929803000	-2.175029000	5.056987000
Te	0.601321000	-0.354069000	6.245129000
C	0.572191000	-1.315590000	8.159980000

H	-0.356520000	-1.045742000	8.664280000
H	0.636479000	-2.395224000	8.003963000
H	1.437941000	-0.962642000	8.722082000
N	-0.669012000	0.405910000	3.851102000
H	-0.916792000	1.314288000	3.468202000
H	-0.349818000	-0.161565000	3.067917000
H	-0.119963000	4.372246000	5.994537000
O	0.443243000	3.658943000	5.663777000
O	-0.116905000	1.273236000	6.998515000
H	0.162033000	2.065254000	6.474690000
H	1.322934000	4.055673000	5.604389000

PC-Te-3 **$C_{10}H_{24}Te_2S_2O_4$**

-7.63727580 Nimag=0

C	-0.633224000	1.799000000	1.382795000
H	0.394288000	1.492772000	1.140970000
H	-1.306827000	0.966251000	1.119272000
C	-0.722039000	2.093080000	2.884561000
H	-0.050546000	2.924330000	3.117168000
C	-2.142373000	2.429411000	3.324563000
H	-2.536151000	3.240605000	2.707686000
H	-2.815209000	1.567044000	3.257775000
Te	-2.289230000	3.205594000	5.372394000
C	-4.391175000	3.675985000	5.106113000
H	-4.461480000	4.384413000	4.279406000
H	-4.954551000	2.763171000	4.899289000
H	-4.740150000	4.135811000	6.032117000
O	-0.980282000	2.973922000	0.665689000
C	-0.796752000	2.837839000	-0.732665000
H	0.252166000	2.619035000	-0.985640000
H	-1.431602000	2.041425000	-1.153107000
H	-1.079099000	3.792267000	-1.184268000
S	-0.169836000	0.617087000	3.863967000
S	1.839704000	0.447108000	3.590818000
C	2.576145000	0.665501000	6.251611000
H	1.529131000	0.400138000	6.462407000
H	3.149191000	-0.273499000	6.171218000
C	2.647263000	1.439339000	4.928890000
H	2.093567000	2.375175000	5.030672000
C	4.087937000	1.716818000	4.491385000
H	4.659106000	2.069896000	5.351871000
H	4.577817000	0.809712000	4.124850000
Te	4.391240000	3.221640000	2.944370000
C	4.507271000	4.904852000	4.301333000
H	3.560389000	5.029706000	4.824256000
H	5.333676000	4.757003000	4.996950000
H	4.705126000	5.778900000	3.677158000
O	3.093324000	1.482984000	7.290424000
C	3.018506000	0.868187000	8.563177000
H	1.978838000	0.635463000	8.843852000
H	3.606550000	-0.062927000	8.603730000
H	3.428703000	1.579281000	9.284799000
O	-1.534742000	4.865039000	5.169328000
O	1.116625000	5.668573000	6.020822000
H	1.104480000	6.630821000	5.964169000
H	0.213505000	5.409625000	5.749858000

PC-Te-4 **$C_8H_{18}TeSO_3$**

-5.77199253 Nimag=0

S	2.547581000	-0.804770000	0.707210000
C	0.446286000	-2.318983000	-0.095341000
H	1.147980000	-2.826679000	-0.771616000
H	0.517112000	-2.810466000	0.890184000
C	0.837104000	-0.843416000	0.015107000
H	0.908128000	-0.425208000	-0.992669000
C	-0.096145000	0.003678000	0.873912000
H	-0.319710000	-0.473052000	1.833935000
H	0.333761000	0.988954000	1.058761000
Te	-2.041057000	0.503883000	-0.047704000
C	-2.452234000	1.967551000	1.507933000
H	-1.646600000	2.702807000	1.493818000
H	-3.405077000	2.441225000	1.264694000
H	-2.516374000	1.467255000	2.477374000
O	-0.883074000	-2.417192000	-0.592890000
C	-1.310985000	-3.756484000	-0.774279000
H	-2.333423000	-3.716634000	-1.158362000
H	-0.677150000	-4.288455000	-1.499886000
H	-1.305317000	-4.317233000	0.173517000
C	3.345635000	0.527348000	-0.279986000
H	2.758420000	1.445315000	-0.201655000
H	4.284029000	0.681727000	0.266537000
C	3.613231000	0.153105000	-1.701767000
H	4.274005000	-0.701245000	-1.850277000
C	3.098302000	0.784457000	-2.760115000
H	2.431103000	1.635774000	-2.651778000
H	3.338159000	0.466735000	-3.771655000
O	-1.515972000	1.588121000	-1.437700000
O	0.558104000	3.446029000	-0.951665000
H	-0.188284000	2.846417000	-1.178178000
H	0.341130000	4.272811000	-1.396957000

P-S-1 **$C_5NH_{13}S_2O_3$**

-4.53921881 Nimag=0

C	0.096985000	0.009281000	2.397879000
H	1.144417000	0.018510000	2.080517000
H	-0.336449000	-0.955817000	2.117863000
C	-0.028895000	0.242838000	3.913179000
H	-1.090551000	0.230544000	4.170993000
H	-0.431263000	0.791326000	1.845886000

C	0.689394000	-0.872484000	4.683505000
H	0.337352000	-1.856660000	4.360242000
H	1.769305000	-0.839041000	4.496960000
S	0.621741000	-0.887665000	6.541178000
C	-1.128839000	-1.365242000	6.734060000
H	-1.771564000	-0.546631000	6.404540000
H	-1.328067000	-2.290374000	6.187164000
H	-1.267591000	-1.528616000	7.805003000
N	0.472497000	1.577500000	4.281736000
H	1.426292000	1.637153000	4.614583000
S	-0.449354000	2.765137000	4.985386000
O	-1.688312000	2.186977000	5.469537000
O	0.441762000	3.501040000	5.856676000
C	-0.886353000	3.852134000	3.628533000
H	-1.475189000	3.297723000	2.896880000
H	-1.483516000	4.658779000	4.058823000
H	0.026920000	4.247187000	3.182946000
O	1.451790000	-2.068291000	6.937179000

P-S-2 $C_4NH_{11}SO$

-3.36556397 Nimag=0

C	-0.367827000	-0.465128000	2.381815000
H	0.480540000	-0.849159000	1.807124000
H	-1.174402000	-1.204717000	2.360555000
C	0.047493000	-0.137703000	3.821113000
H	-0.861588000	0.195188000	4.351247000
H	-0.732751000	0.437464000	1.878862000
C	0.580760000	-1.396332000	4.520244000
H	-0.033921000	-2.277546000	4.309027000
H	1.600527000	-1.598835000	4.177128000
S	0.732751000	-1.359452000	6.365139000
C	-1.042814000	-1.463168000	6.791936000
H	-1.597130000	-0.622571000	6.370233000
H	-1.444300000	-2.418428000	6.443800000
H	-1.081701000	-1.422447000	7.882526000
N	1.098500000	0.885163000	3.807858000
H	1.344782000	1.126865000	4.764441000
H	0.745288000	1.731091000	3.368783000
O	1.203718000	-0.000772000	6.792274000

P-S-2-H$^+$ $C_4NH_{12}SO^+$

-3.28164478 Nimag=0

C	-0.414043000	0.639255000	2.965453000
H	0.532116000	1.183068000	2.879779000
H	-0.393878000	-0.185122000	2.248083000
C	-0.630639000	0.095526000	4.371998000
H	-1.587751000	-0.432261000	4.414266000
H	-1.230945000	1.307076000	2.673624000
C	0.493619000	-0.832012000	4.847620000
H	0.587398000	-1.689706000	4.176988000
H	1.458760000	-0.313745000	4.856424000
S	0.313048000	-1.492642000	6.572100000
C	-0.960112000	-2.758202000	6.324754000
H	-1.866004000	-2.354938000	5.869057000
H	-0.527563000	-3.561720000	5.722073000
H	-1.183128000	-3.135894000	7.325729000
N	-0.775285000	1.220006000	5.385101000
H	-0.706043000	0.731089000	6.361360000
H	-1.662029000	1.712637000	5.274630000
H	-0.023698000	1.906360000	5.296923000
O	-0.337600000	-0.350011000	7.349531000

P-S-3 $C_{10}H_{22}S_4O_3$

-7.25642422 Nimag=0

C	-0.284170000	2.710987000	1.751154000
H	0.695096000	2.531085000	1.284535000
H	-0.981724000	1.943779000	1.374689000
C	-0.131225000	2.590018000	3.270533000
H	0.568717000	3.354002000	3.616699000
C	-1.456999000	2.761769000	4.007681000
H	-1.953826000	3.661815000	3.635038000
H	-2.123119000	1.900958000	3.874733000
S	-1.217587000	2.973349000	5.832652000
C	-2.936477000	3.488216000	6.179759000
H	-3.176495000	4.393539000	5.616734000
H	-3.619215000	2.671483000	5.929564000
H	-2.980866000	3.690074000	7.252083000
O	-0.752484000	4.013332000	1.431252000
C	-0.817483000	4.244720000	0.035103000
H	0.171208000	4.147760000	-0.439723000
H	-1.513625000	3.550744000	-0.462808000
H	-1.177836000	5.267539000	-0.101645000
S	0.605781000	0.941027000	3.695438000
S	2.609918000	1.173617000	3.429753000
C	3.474199000	0.607862000	5.999838000
H	2.546776000	0.019614000	6.061073000
H	4.264804000	-0.055533000	5.610210000
C	3.259769000	1.790420000	5.050032000
H	2.496914000	2.446861000	5.471608000
C	4.553091000	2.581676000	4.788904000
H	5.104036000	2.664211000	5.730195000
H	5.201323000	2.040087000	4.090605000
S	4.377152000	4.244369000	4.074271000
C	3.846737000	5.212566000	5.513745000
H	2.859177000	4.916078000	5.873431000
H	4.579467000	5.151000000	6.323505000
H	3.791781000	6.247985000	5.167345000
O	3.826068000	1.104148000	7.284844000
C	4.001834000	0.074519000	8.240757000

H	3.081519000	-0.514683000	8.379719000
H	4.815424000	-0.612562000	7.957690000
H	4.259114000	0.557766000	9.186927000
O	-0.341680000	4.171329000	6.031632000

P-S-4 **$C_8H_{16}S_2O_2$**

-5.32056365 Nimag=0

S	2.875047000	-0.047789000	1.201628000
C	0.979065000	-1.827566000	0.454796000
H	1.763066000	-2.344625000	-0.117384000
H	1.037536000	-2.187848000	1.497069000
C	1.238543000	-0.317744000	0.406283000
H	1.359665000	-0.018057000	-0.638422000
C	0.140237000	0.527241000	1.069408000
H	-0.378487000	-0.032292000	1.853445000
H	0.556137000	1.441227000	1.499572000
S	-1.132500000	1.128375000	-0.143440000
C	-2.178535000	1.967796000	1.101244000
H	-1.586071000	2.690686000	1.668029000
H	-2.958757000	2.484263000	0.538047000
H	-2.630596000	1.221242000	1.759808000
O	-0.303052000	-2.106951000	-0.085325000
C	-0.607466000	-3.490056000	-0.082915000
H	-1.605886000	-3.597276000	-0.514570000
H	0.108125000	-4.066245000	-0.690247000
H	-0.611717000	-3.906009000	0.937480000
C	3.539117000	1.362447000	0.226245000
H	2.812046000	2.179184000	0.227419000
H	4.399376000	1.678580000	0.828110000
C	3.963573000	0.997613000	-1.159468000
H	4.779634000	0.278256000	-1.228870000
C	3.410031000	1.478533000	-2.275558000
H	2.583757000	2.184894000	-2.250819000
H	3.769901000	1.174767000	-3.255131000
O	-0.463858000	2.198777000	-0.952576000

P-Se-1 **$C_5NH_{13}SeSO_3$**

-4.48771209 Nimag=0

C	0.761302000	-0.063382000	2.241561000
H	1.847612000	-0.163303000	2.150660000
H	0.305499000	-0.995967000	1.895424000
C	0.347997000	0.235542000	3.694032000
H	-0.739884000	0.335194000	3.723104000
H	0.437111000	0.742794000	1.577607000
C	0.773826000	-0.906995000	4.619683000
H	0.416195000	-1.872818000	4.255764000
H	1.863659000	-0.968387000	4.709411000
Se	0.206864000	-0.866683000	6.562852000
C	-1.689297000	-1.370065000	6.237637000
H	-2.202868000	-0.554067000	5.727056000
H	-1.703013000	-2.297134000	5.663172000
H	-2.118828000	-1.529180000	7.227958000
N	0.886062000	1.533765000	4.128638000
H	1.826567000	1.549526000	4.501846000
S	-0.012418000	2.785865000	4.733217000
O	-1.330948000	2.294127000	5.083501000
O	0.832791000	3.460714000	5.695276000
C	-0.230363000	3.899484000	3.344943000
H	-0.776963000	3.386084000	2.552961000
H	-0.810783000	4.747266000	3.714787000
H	0.749586000	4.229041000	2.998513000
O	0.905221000	-2.255347000	7.136470000

P-Se-2 **$C_4NH_{11}SeO$**

-3.31411965 Nimag=0

C	-0.450392000	-0.621561000	2.202458000
H	0.490827000	-0.668030000	1.646151000
H	-0.806147000	-1.640796000	2.382489000
C	-0.272357000	0.146354000	3.519038000
H	-1.245376000	0.122764000	4.041235000
H	-1.191010000	-0.119026000	1.569913000
C	0.761164000	-0.554422000	4.403735000
H	0.589531000	-1.632643000	4.474597000
H	1.771861000	-0.373912000	4.026480000
Se	0.846589000	0.060433000	6.325681000
C	-0.757240000	-0.941223000	6.943985000
H	-1.615415000	-0.644910000	6.340863000
H	-0.562944000	-2.013958000	6.871314000
H	-0.902083000	-0.646964000	7.984481000
N	0.182800000	1.511337000	3.238297000
H	0.213430000	2.033798000	4.111947000
H	-0.493425000	1.977590000	2.639449000
O	0.353763000	1.647546000	6.340218000

P-Se-2-H^+ **$C_4NH_{12}SeO^+$**

-3.23866419 Nimag=0

C	1.042646000	-0.255548000	3.171495000
H	2.034460000	-0.571802000	3.510393000
H	0.630385000	-1.046294000	2.537999000
C	0.114147000	0.046344000	4.351000000
H	-0.858438000	0.351562000	3.951922000
H	1.160322000	0.638052000	2.551029000
C	-0.080600000	-1.198493000	5.226522000
H	-0.525067000	-2.028054000	4.670029000
H	0.859037000	-1.545560000	5.668383000
Se	-1.258158000	-0.962133000	6.817414000
C	-2.994696000	-0.685696000	5.954168000
H	-2.926678000	0.086248000	5.189527000
H	-3.304523000	-1.648670000	5.539338000
H	-3.669013000	-0.383324000	6.757785000

N	0.572126000	1.172596000	5.212906000
H	1.566895000	1.089014000	5.419878000
H	0.454210000	2.059955000	4.728409000
O	-0.813643000	0.651203000	7.289485000
H	-0.226371000	1.027232000	6.460299000

P-Se-3 $C_{10}H_{22}Se_2S_2O_3$

-7.16856020 Nimag=0

C	-0.104367000	0.703601000	2.198882000
H	0.916742000	0.378714000	1.954583000
H	-0.765557000	-0.176062000	2.129631000
C	-0.114466000	1.268792000	3.620621000
H	0.566861000	2.123976000	3.679258000
C	-1.500538000	1.688467000	4.100565000
H	-2.210898000	1.773317000	3.277064000
H	-1.916514000	1.025009000	4.863572000
Se	-1.471173000	3.522004000	5.007268000
C	-2.100775000	4.532536000	3.417216000
H	-1.445381000	4.297183000	2.579164000
H	-3.137648000	4.257486000	3.208275000
H	-2.029466000	5.586583000	3.689654000
O	-0.528738000	1.716329000	1.293486000
C	-0.473418000	1.297046000	-0.058973000
H	0.551064000	1.029306000	-0.360062000
H	-1.129540000	0.431895000	-0.246044000
H	-0.813366000	2.138220000	-0.668941000
S	0.532793000	0.007574000	4.817000000
S	2.552580000	-0.004326000	4.586570000
C	3.234072000	0.763727000	7.158834000
H	2.237921000	0.379093000	7.422532000
H	3.947748000	-0.074007000	7.234067000
C	3.200927000	1.305267000	5.725169000
H	2.503707000	2.143224000	5.680239000
C	4.588310000	1.730771000	5.231697000
H	5.097733000	2.273714000	6.030205000
H	5.206692000	0.861650000	4.983964000
Se	4.664114000	2.870225000	3.622366000
C	4.138384000	4.578423000	4.439065000
H	3.103578000	4.548235000	4.779532000
H	4.814507000	4.829176000	5.258308000
H	4.237606000	5.322975000	3.646009000
O	3.602717000	1.814622000	8.043036000
C	3.602632000	1.413254000	9.400174000
H	2.606209000	1.074376000	9.725828000
H	4.324204000	0.601679000	9.587835000
H	3.890032000	2.286522000	9.991764000
O	0.131790000	3.931876000	5.119560000

P-Se-4 $C_8H_{16}SeSO_2$

-5.26953721 Nimag=0

S	3.180265000	-0.544351000	0.704905000
C	0.936736000	-2.012011000	0.324288000
H	1.534756000	-2.658542000	-0.334047000
H	1.091933000	-2.358353000	1.361025000
C	1.415044000	-0.564437000	0.174920000
H	1.421109000	-0.304998000	-0.887719000
C	0.593682000	0.454337000	0.961860000
H	0.234222000	0.053581000	1.914125000
H	1.159382000	1.369159000	1.141914000
Se	-1.011217000	1.149382000	-0.061854000
C	-1.574136000	2.356628000	1.420497000
H	-0.734879000	3.005466000	1.674738000
H	-2.406494000	2.944223000	1.030123000
H	-1.895468000	1.758288000	2.276663000
O	-0.440494000	-2.091140000	-0.013437000
C	-0.953438000	-3.408519000	0.077398000
H	-2.009206000	-3.359496000	-0.201212000
H	-0.434208000	-4.095573000	-0.608772000
H	-0.871045000	-3.808948000	1.100434000
C	3.885447000	0.744686000	-0.401017000
H	3.305450000	1.666360000	-0.300784000
H	4.871653000	0.921022000	0.044329000
C	4.011882000	0.321127000	-1.828536000
H	4.693655000	-0.508432000	-2.016214000
C	3.349065000	0.875415000	-2.846829000
H	2.646176000	1.692422000	-2.701762000
H	3.489211000	0.521740000	-3.864964000
O	-0.326484000	2.168739000	-1.178486000

P-Te-1 $C_5NH_{13}TeSO_3$

-4.46475998 Nimag=0

C	-0.379599000	-0.159673000	2.364805000
H	0.592260000	-0.061199000	1.872147000
H	-0.769192000	-1.165580000	2.176887000
C	-0.248516000	0.083946000	3.875424000
H	-1.240629000	-0.049541000	4.327619000
H	-1.064595000	0.563099000	1.920343000
C	0.698505000	-0.954109000	4.484318000
H	0.306417000	-1.964874000	4.335727000
H	1.689927000	-0.902480000	4.021636000
Te	1.173861000	-0.794787000	6.624711000
C	-0.874717000	-0.719117000	7.334192000
H	-1.330869000	0.196110000	6.957847000
H	-1.410449000	-1.608896000	6.995224000
H	-0.822967000	-0.702635000	8.424279000
N	0.269187000	1.449912000	4.161810000
H	0.791818000	1.452733000	5.053134000
S	-0.881300000	2.674231000	4.276678000
O	-1.499695000	2.846875000	2.976374000

O	-1.751848000	2.493225000	5.430060000
C	0.201405000	4.059671000	4.608612000
H	0.876134000	4.194753000	3.763664000
H	-0.457702000	4.922667000	4.721063000
H	0.747712000	3.878037000	5.535147000
O	1.690109000	0.968617000	6.707706000

P-Te-2 **$C_4NH_{11}TeO$**

-3.29006042 Nimag=0

C	-0.717310000	-0.636976000	2.067415000
H	0.164958000	-1.221195000	1.783812000
H	-1.579869000	-1.312721000	2.085919000
C	-0.511573000	0.046842000	3.431499000
H	-1.426774000	0.613269000	3.655260000
H	-0.896090000	0.109592000	1.285544000
C	-0.309794000	-0.974772000	4.548923000
H	-1.217305000	-1.546667000	4.758111000
H	0.509471000	-1.669656000	4.332950000
Te	0.389255000	-0.002740000	6.392709000
C	-0.282598000	-1.680038000	7.616778000
H	-1.373667000	-1.744831000	7.603537000
H	0.172543000	-2.587919000	7.217388000
H	0.072929000	-1.499026000	8.632728000
N	0.583169000	1.022949000	3.483721000
H	0.468063000	1.738872000	2.772375000
H	1.482411000	0.576422000	3.318520000
O	2.185848000	-0.335399000	6.322097000

P-Te-2-H^+ **$C_4NH_{12}TeO^+$**

-3.22306156 Nimag=0

C	-1.001517000	-0.363804000	2.924829000
H	-1.019493000	0.722086000	2.786203000
H	-0.220607000	-0.777448000	2.279950000
C	-0.767972000	-0.739675000	4.393037000
H	-0.756056000	-1.832528000	4.464350000
H	-1.960013000	-0.768180000	2.585072000
C	0.585627000	-0.200534000	4.878398000
H	1.416714000	-0.588683000	4.282563000
H	0.629703000	0.893168000	4.846935000
Te	1.100614000	-0.675763000	6.944628000
C	1.294583000	-2.799975000	6.733290000
H	0.410018000	-3.200771000	6.239828000
H	2.202894000	-2.999943000	6.159066000
H	1.390084000	-3.200274000	7.744413000
N	-1.839749000	-0.283144000	5.315666000
H	-1.278256000	-0.483008000	6.813941000
H	-2.699414000	-0.795230000	5.128492000
H	-2.058811000	0.698743000	5.150252000
O	-0.671729000	-0.585905000	7.655723000

P-Te-3 **$C_{10}H_{22}Te_2S_2O_3$**

-7.11389304 Nimag=0

C	-0.648879000	1.599000000	1.292122000
H	0.363464000	1.282143000	1.002470000
H	-1.343579000	0.778052000	1.047309000
C	-0.665182000	1.877531000	2.798926000
H	0.038573000	2.684252000	3.019940000
C	-2.044431000	2.253235000	3.321471000
H	-2.460430000	3.062525000	2.718092000
H	-2.745805000	1.411360000	3.321845000
Te	-1.957190000	3.019442000	5.392446000
C	-3.558702000	4.443810000	5.016170000
H	-3.192634000	5.139926000	4.259799000
H	-4.452794000	3.917089000	4.673779000
H	-3.761597000	4.968383000	5.951362000
O	-1.011335000	2.784925000	0.598791000
C	-0.894872000	2.654173000	-0.806776000
H	0.138242000	2.422874000	-1.109609000
H	-1.558615000	1.868102000	-1.201544000
H	-1.184961000	3.614294000	-1.241393000
S	-0.082041000	0.381475000	3.726879000
S	1.946437000	0.372703000	3.549713000
C	2.645214000	0.534143000	6.218298000
H	1.655153000	0.088244000	6.397120000
H	3.363884000	-0.291589000	6.083151000
C	2.589167000	1.398043000	4.951240000
H	1.877950000	2.211054000	5.108452000
C	3.963056000	1.949122000	4.560792000
H	4.466637000	2.318446000	5.455980000
H	4.601727000	1.174086000	4.125365000
Te	4.017357000	3.596060000	3.136186000
C	3.426734000	5.129362000	4.546085000
H	2.403095000	4.960841000	4.877384000
H	4.121226000	5.141115000	5.386439000
H	3.489660000	6.075447000	4.004615000
O	3.021456000	1.349258000	7.319726000
C	3.065117000	0.634575000	8.540505000
H	2.083561000	0.205584000	8.798420000
H	3.804185000	-0.182319000	8.510986000
H	3.354939000	1.346925000	9.317417000
O	-0.479115000	4.095669000	5.331231000

P-Te-4 **$C_8H_{16}TeSO_2$**

-5.24540220 Nimag=0

S	2.564769000	-0.872846000	0.684217000
C	0.449526000	-2.383480000	-0.088644000
H	1.142225000	-2.892734000	-0.773502000
H	0.530648000	-2.875447000	0.896043000
C	0.843039000	-0.907988000	0.018503000
H	0.905796000	-0.491412000	-0.990838000

C	-0.090778000	-0.062977000	0.877991000
H	-0.373575000	-0.567948000	1.807173000
H	0.367057000	0.897033000	1.122111000
Te	-1.949008000	0.570384000	-0.147486000
C	-2.350679000	2.040716000	1.412705000
H	-1.514941000	2.742540000	1.419704000
H	-3.274651000	2.557702000	1.147261000
H	-2.459590000	1.544153000	2.380167000
O	-0.885109000	-2.477062000	-0.570021000
C	-1.318835000	-3.814761000	-0.744838000
H	-2.345925000	-3.771786000	-1.116038000
H	-0.695998000	-4.350108000	-1.477799000
H	-1.303447000	-4.374925000	0.203340000
C	3.298659000	0.536173000	-0.242125000
H	2.675393000	1.424545000	-0.105980000
H	4.239370000	0.704837000	0.295212000
C	3.553026000	0.251169000	-1.686706000
H	4.279875000	-0.535092000	-1.890929000
C	2.950994000	0.873865000	-2.703021000
H	2.206883000	1.651334000	-2.546118000
H	3.183784000	0.619043000	-3.733564000
O	-1.250790000	1.623188000	-1.471515000

TS-S-1 **$C_5NH_{15}S_2O_4$**

-4.94622122 Nimag=1 ν=-535.3

C	-0.324302000	0.252636000	2.452604000
H	0.690420000	0.378408000	2.063684000
H	-0.729188000	-0.691594000	2.076334000
C	-0.336618000	0.263438000	3.985491000
H	-1.376118000	0.157900000	4.313126000
H	-0.952669000	1.054119000	2.058386000
C	0.469958000	-0.932563000	4.512782000
H	0.060355000	-1.864695000	4.115729000
H	1.516581000	-0.869333000	4.197324000
S	0.586854000	-1.133827000	6.312722000
C	-1.114720000	-1.435743000	6.811794000
H	-1.728449000	-0.544398000	6.668303000
H	-1.509316000	-2.292741000	6.262693000
H	-1.068329000	-1.679836000	7.874906000
N	0.231773000	1.518517000	4.525812000
H	0.726529000	1.379472000	5.402688000
S	-0.743853000	2.871304000	4.766925000
O	-2.124262000	2.533908000	4.480099000
O	-0.351163000	3.420198000	6.048994000
C	-0.190954000	4.013773000	3.505115000
H	-0.375479000	3.587290000	2.519456000
H	-0.779353000	4.923488000	3.641671000
H	0.869555000	4.216104000	3.655759000
O	1.113273000	-3.050883000	6.243045000
O	1.884119000	-4.880346000	6.594479000
H	1.857985000	-3.189646000	6.866290000
H	2.242211000	-4.986925000	5.700793000

TS-S-2 **$C_4NH_{13}SO_2$**

-3.77415099 Nimag=1 ν=-426.8

C	-0.258773000	-0.532755000	2.384703000
H	0.701209000	-0.747170000	1.904405000
H	-0.903749000	-1.411975000	2.293449000
C	-0.062694000	-0.128939000	3.851460000
H	-1.065389000	0.035067000	4.282141000
H	-0.734826000	0.284511000	1.832805000
C	0.595220000	-1.305950000	4.604020000
H	0.148410000	-2.260417000	4.305295000
H	1.662202000	-1.335809000	4.362097000
S	0.564442000	-1.326033000	6.427064000
C	-1.154257000	-1.706689000	6.793641000
H	-1.805297000	-0.906195000	6.439520000
H	-1.427724000	-2.664295000	6.343593000
H	-1.224726000	-1.776991000	7.880553000
N	0.792445000	1.052319000	3.926591000
H	0.757983000	1.446468000	4.864441000
H	0.438240000	1.767789000	3.298665000
O	0.382552000	0.606165000	6.886629000
O	0.628039000	2.553653000	7.362526000
H	1.222754000	0.856243000	7.314414000
H	-0.110713000	2.575569000	7.987833000

TS-S-2-H^+ **$C_4NH_{14}SO_2^+$**

-3.70405868 Nimag=1 ν=-229.7

C	-0.527308000	-0.371955000	2.935153000
H	0.420116000	-0.920549000	2.952934000
H	-1.308590000	-1.062544000	2.609850000
C	-0.882469000	0.205145000	4.306928000
H	-1.833943000	0.736970000	4.239400000
H	-0.460002000	0.416936000	2.178809000
C	-0.964176000	-0.916262000	5.368440000
H	-1.359854000	-1.806655000	4.872597000
H	0.036481000	-1.190374000	5.721497000
S	-1.942763000	-0.732146000	6.898303000
C	-3.645922000	-0.710952000	6.302324000
H	-3.854841000	0.170212000	5.693281000
H	-3.864400000	-1.630076000	5.752423000
H	-4.267453000	-0.677950000	7.200345000
N	0.134306000	1.281178000	4.663062000
H	0.991330000	0.870980000	5.038474000
H	-0.216146000	2.017909000	5.375076000
H	0.400218000	1.787126000	3.816353000
O	-1.385134000	1.517456000	6.911649000
O	-0.645407000	3.033812000	6.526798000

H	-1.230219000	1.580186000	7.871616000
H	-1.453694000	3.555098000	6.390677000

TS-S-3 $C_{10}H_{24}S_4O_4$

-7.66041126 Nimag=1 ν=-464.4

C	-0.331341000	2.648394000	1.695286000
H	0.694962000	2.583756000	1.308354000
H	-0.914672000	1.827438000	1.245131000
C	-0.282543000	2.498457000	3.217799000
H	0.306272000	3.313650000	3.641947000
C	-1.685076000	2.529040000	3.827855000
H	-2.195241000	3.430952000	3.480351000
H	-2.276295000	1.656734000	3.523514000
S	-1.664607000	2.554965000	5.653692000
C	-3.331605000	3.165661000	5.980131000
H	-3.495778000	4.137372000	5.507969000
H	-4.072756000	2.443592000	5.625147000
H	-3.414360000	3.266177000	7.064184000
O	-0.906438000	3.907504000	1.374397000
C	-0.833735000	4.210048000	-0.009042000
H	0.208338000	4.246414000	-0.360999000
H	-1.384663000	3.475354000	-0.617562000
H	-1.288319000	5.194846000	-0.142961000
S	0.555263000	0.913553000	3.689971000
S	2.542010000	1.263446000	3.428664000
C	3.260599000	0.936299000	6.088043000
H	2.299694000	0.407818000	6.172052000
H	4.027322000	0.193487000	5.809929000
C	3.153664000	2.014917000	5.006173000
H	2.427158000	2.767058000	5.319252000
C	4.508876000	2.680718000	4.704378000
H	5.017207000	2.860066000	5.656043000
H	5.147855000	2.000022000	4.129898000
S	4.493024000	4.232775000	3.757458000
C	4.053098000	5.449447000	5.030740000
H	3.034848000	5.325144000	5.403501000
H	4.771778000	5.426655000	5.855429000
H	4.120941000	6.422266000	4.535979000
O	3.588078000	1.554007000	7.325469000
C	3.651009000	0.632363000	8.397616000
H	2.687348000	0.121702000	8.554227000
H	4.427475000	-0.132789000	8.235927000
H	3.898602000	1.204053000	9.295879000
O	-0.451084000	4.135425000	6.080000000
O	0.513396000	5.863121000	6.245492000
H	0.484564000	5.847020000	7.213061000
H	-0.882449000	4.918524000	5.687077000

TS-S-4 $C_8H_{18}S_2O_3$

-5.72697426 Nimag=1 ν=-422.0

S	-3.976096000	4.463612000	0.861545000
C	-2.813430000	2.970791000	2.795413000
H	-2.714315000	3.922042000	3.338244000
H	-3.820121000	2.571363000	3.011010000
C	-2.665246000	3.247174000	1.296240000
H	-1.702165000	3.729369000	1.114666000
C	-2.817753000	2.024695000	0.379859000
H	-3.699557000	1.438627000	0.657386000
H	-2.926156000	2.354070000	-0.655038000
S	-1.395494000	0.873990000	0.356935000
C	-1.925280000	-0.219044000	-0.974719000
H	-2.056453000	0.355417000	-1.894023000
H	-1.125631000	-0.950045000	-1.113288000
H	-2.846398000	-0.738982000	-0.696496000
O	-1.819661000	2.050644000	3.214125000
C	-1.899303000	1.747064000	4.595991000
H	-1.094897000	1.040104000	4.813396000
H	-1.764443000	2.645503000	5.218172000
H	-2.864073000	1.283456000	4.856435000
C	-3.135166000	5.557734000	-0.353372000
H	-2.727917000	4.958923000	-1.172412000
H	-3.977862000	6.136019000	-0.752253000
C	-2.105457000	6.462858000	0.241333000
H	-2.461260000	7.134983000	1.022221000
C	-0.822186000	6.491386000	-0.125244000
H	-0.430870000	5.834583000	-0.898208000
H	-0.123484000	7.185214000	0.334878000
O	-0.294846000	2.136682000	-0.762484000
O	1.066747000	3.080761000	-1.903474000
H	0.610137000	1.775815000	-0.740409000
H	1.217233000	3.802060000	-1.274843000

TS-Se-1 $C_5NH_{15}SeSO_4$

-4.91781343 Nimag=1 ν=-472.0

C	0.292699000	0.318593000	2.226551000
H	1.366612000	0.382953000	2.028664000
H	-0.094471000	-0.592776000	1.761745000
C	0.005877000	0.307078000	3.733578000
H	-1.080551000	0.262317000	3.866391000
H	-0.205827000	1.164009000	1.747509000
C	0.633974000	-0.939313000	4.362632000
H	0.262020000	-1.846761000	3.883985000
H	1.724585000	-0.929398000	4.271563000
Se	0.352176000	-1.238188000	6.295641000
C	-1.582371000	-1.466444000	6.334045000
H	-2.079911000	-0.529977000	6.083264000
H	-1.844301000	-2.270090000	5.645644000
H	-1.819539000	-1.758696000	7.357725000
N	0.544443000	1.516718000	4.397196000

H	0.812045000	1.344053000	5.362590000
S	-0.338916000	2.951867000	4.428783000
O	-1.645569000	2.736008000	3.839440000
O	-0.194294000	3.475639000	5.772279000
C	0.580383000	4.029967000	3.335364000
H	0.584294000	3.613204000	2.328576000
H	0.058974000	4.989341000	3.347974000
H	1.593889000	4.138645000	3.721749000
O	0.614460000	-3.313840000	6.183683000
O	1.261865000	-5.232704000	6.241542000
H	1.470083000	-3.560021000	6.594393000
H	0.563921000	-5.512433000	6.852586000

TS-Se-2 **$C_4NH_{13}SeO_2$**

-3.74597891 Nimag=1 ν=-352.7

C	-0.191364000	-0.111901000	2.386359000
H	0.818122000	-0.174260000	1.967716000
H	-0.690845000	-1.075593000	2.249199000
C	-0.148972000	0.296460000	3.865708000
H	-1.191646000	0.320327000	4.226739000
H	-0.749164000	0.634463000	1.810700000
C	0.604896000	-0.791877000	4.650863000
H	0.272495000	-1.794880000	4.368597000
H	1.679884000	-0.708170000	4.467734000
Se	0.481215000	-0.792028000	6.628129000
C	-1.326374000	-1.491381000	6.841482000
H	-2.034803000	-0.777708000	6.422262000
H	-1.399883000	-2.465026000	6.354326000
H	-1.487625000	-1.592142000	7.915542000
N	0.531274000	1.581561000	4.002350000
H	0.339835000	1.976685000	4.921171000
H	0.152305000	2.237644000	3.325942000
O	-0.147983000	1.195192000	6.919413000
O	-0.327779000	3.199547000	7.184558000
H	-1.178978000	3.136075000	7.641060000
H	0.556128000	1.627497000	7.436031000

TS-Se-2-H$^+$ **$C_4NH_{14}SeO_2^+$**

-3.67441132 Nimag=1 ν=-190.8

C	-0.288843000	-0.605830000	3.157917000
H	0.668879000	-1.126371000	3.263503000
H	-1.047079000	-1.352423000	2.910842000
C	-0.689912000	0.122958000	4.441230000
H	-1.643499000	0.632719000	4.290351000
H	-0.222473000	0.081435000	2.308014000
C	-0.770211000	-0.836565000	5.646064000
H	-0.952491000	-1.847395000	5.278453000
H	0.173413000	-0.873555000	6.199307000
Se	-2.136068000	-0.552347000	7.054210000
C	-3.749899000	-0.986587000	6.027010000
H	-3.911980000	-0.276115000	5.216022000
H	-3.675842000	-2.008704000	5.651365000
H	-4.571575000	-0.917769000	6.742609000
N	0.300257000	1.255609000	4.683268000
H	1.223778000	0.886329000	4.918764000
H	0.018826000	1.958439000	5.438365000
H	0.414492000	1.793973000	3.821665000
O	-1.358156000	1.786465000	6.898423000
O	-0.463993000	3.165678000	6.482317000
H	-2.178490000	2.248424000	7.149602000
H	0.026716000	3.263760000	7.315612000

TS-Se-3 **$C_{10}H_{24}Se_2S_2O_4$**

-7.59777410 Nimag=1 ν=-363.3

C	-0.101601000	0.860808000	2.348169000
H	0.958048000	0.652761000	2.150200000
H	-0.665917000	-0.073284000	2.188944000
C	-0.248359000	1.324997000	3.797200000
H	0.325709000	2.238410000	3.952129000
C	-1.718574000	1.514280000	4.213438000
H	-2.393477000	1.343234000	3.372366000
H	-2.012493000	0.835132000	5.019673000
Se	-2.266800000	3.270176000	4.934751000
C	-2.155261000	4.367131000	3.321701000
H	-1.132578000	4.349403000	2.950011000
H	-2.856066000	3.991165000	2.575258000
H	-2.433373000	5.375356000	3.633525000
O	-0.574558000	1.882611000	1.478097000
C	-0.370803000	1.570214000	0.109648000
H	0.697075000	1.439509000	-0.121565000
H	-0.907501000	0.654275000	-0.184339000
H	-0.759159000	2.410867000	-0.471325000
S	0.456495000	0.044987000	4.949822000
S	2.461989000	0.020131000	4.633287000
C	3.263330000	0.701511000	7.190525000
H	2.270466000	0.326861000	7.481068000
H	3.962653000	-0.151282000	7.215408000
C	3.189786000	1.283992000	5.774162000
H	2.525362000	2.149557000	5.774093000
C	4.567973000	1.672687000	5.225608000
H	5.129111000	2.184102000	6.010070000
H	5.145529000	0.788985000	4.935382000
Se	4.595731000	2.852031000	3.642719000
C	4.366687000	4.578430000	4.555607000
H	3.420329000	4.614781000	5.095510000
H	5.210099000	4.761090000	5.224101000
H	4.366842000	5.327241000	3.759929000
O	3.683808000	1.719032000	8.089335000
C	3.763021000	1.268422000	9.428793000

H	2.787742000	0.914295000	9.799499000
H	4.495211000	0.452952000	9.544110000
H	4.083262000	2.120830000	10.033624000
O	-0.372102000	3.797887000	5.751133000
O	1.229672000	4.674628000	6.579279000
H	-0.563863000	4.601116000	6.267522000
H	1.333505000	4.011721000	7.278237000

TS-Se-4 **$C_8H_{18}SeSO_3$**

-5.70056072 Nimag=1 ν=-340.2

S	3.162362000	-0.655587000	0.838422000
C	0.855646000	-1.924909000	0.180905000
H	1.470950000	-2.503376000	-0.523006000
H	0.912860000	-2.426609000	1.162561000
C	1.416422000	-0.504305000	0.274300000
H	1.438528000	-0.064499000	-0.724827000
C	0.683643000	0.426372000	1.240972000
H	0.543502000	-0.038274000	2.220329000
H	1.234549000	1.359100000	1.357546000
Se	-1.116055000	1.032247000	0.650767000
C	-1.271611000	2.510852000	1.925327000
H	-0.472443000	3.225309000	1.725232000
H	-2.244155000	2.974294000	1.749643000
H	-1.222143000	2.127629000	2.946577000
O	-0.491377000	-1.868257000	-0.263367000
C	-1.093805000	-3.146768000	-0.369837000
H	-2.116973000	-2.988962000	-0.719715000
H	-0.563171000	-3.785698000	-1.092353000
H	-1.122874000	-3.665442000	0.601373000
C	4.009154000	0.666184000	-0.122373000
H	3.500652000	1.620187000	0.039761000
H	4.983050000	0.719252000	0.379509000
C	4.173799000	0.363878000	-1.576431000
H	4.760849000	-0.524417000	-1.810287000
C	3.659518000	1.101352000	-2.563597000
H	3.057483000	1.987235000	-2.374963000
H	3.830467000	0.833439000	-3.603401000
O	-0.171774000	2.135915000	-0.897129000
O	0.444690000	3.382322000	-2.362439000
H	-0.885295000	2.626237000	-1.342008000
H	0.526610000	2.672943000	-3.017231000

TS-Te-1 **$C_5NH_{15}TeSO_4$**

-4.89727477 Nimag=1 ν=-237.6

C	0.719555000	-0.303536000	2.399220000
H	1.813201000	-0.362272000	2.365022000
H	0.321803000	-1.274587000	2.089098000
C	0.224473000	0.062507000	3.808865000
H	-0.865484000	0.125679000	3.775715000
H	0.389539000	0.445900000	1.676517000
C	0.646536000	-1.008368000	4.822733000
H	0.391633000	-2.010944000	4.472287000
H	1.724702000	-0.988858000	5.006188000
Te	-0.235508000	-0.888652000	6.802724000
C	-2.024241000	-1.970022000	6.324351000
H	-2.545091000	-1.487686000	5.497235000
H	-1.726269000	-2.991689000	6.088959000
H	-2.646882000	-1.959157000	7.219880000
N	0.701557000	1.387040000	4.251556000
H	1.686268000	1.543959000	4.057363000
S	-0.117397000	2.796541000	3.838347000
O	0.867225000	3.707352000	3.290463000
O	-1.320422000	2.445770000	3.110999000
C	-0.625486000	3.454547000	5.424285000
H	0.261254000	3.655750000	6.025980000
H	-1.161096000	4.381866000	5.210771000
H	-1.281831000	2.738007000	5.917740000
O	0.550447000	-2.903178000	7.408238000
O	1.182383000	-4.661187000	8.112717000
H	1.578800000	-4.919270000	7.267514000
H	1.184153000	-2.741189000	8.124702000

TS-Te-2 **$C_4NH_{13}TeO_2$**

-3.72686729 Nimag=1 ν=-163.9

C	-0.891686000	-0.540946000	2.105245000
H	-0.005545000	-1.135727000	1.859558000
H	-1.762111000	-1.206570000	2.095977000
C	-0.732494000	0.154199000	3.468854000
H	-1.643699000	0.738114000	3.655408000
H	-1.033682000	0.202037000	1.313177000
C	-0.591721000	-0.868647000	4.595259000
H	-1.519228000	-1.416830000	4.772206000
H	0.224786000	-1.572988000	4.413526000
Te	-0.020686000	0.134347000	6.425920000
C	-0.420107000	-1.567263000	7.686872000
H	-1.478679000	-1.826975000	7.647666000
H	0.206474000	-2.390422000	7.342690000
H	-0.140871000	-1.288022000	8.704132000
N	0.381203000	1.105720000	3.562447000
H	0.288213000	1.842001000	2.868729000
H	1.270109000	0.640123000	3.391394000
O	3.830612000	-1.428909000	5.559562000
O	2.091066000	-0.709925000	6.039414000
H	2.655271000	0.057975000	6.209999000
H	3.820799000	-2.061617000	6.292771000

TS-Te-2-H$^+$ **$C_4NH_{14}TeO_2^+$**

-3.64942974 Nimag=1 ν=-198.9

C	-2.594011000	-0.775487000	3.884504000
H	-2.128662000	-0.935382000	2.906443000

H	-2.946972000	-1.742465000	4.249913000
C	-1.613465000	-0.167696000	4.887753000
H	-2.122492000	-0.027049000	5.845772000
H	-3.472684000	-0.135515000	3.753373000
C	-0.362240000	-1.032904000	5.075963000
H	-0.674767000	-2.068790000	5.221327000
H	0.275626000	-1.017971000	4.186175000
Te	1.006248000	-0.654665000	6.744500000
C	-0.352592000	-1.255683000	8.311236000
H	-1.197731000	-0.570780000	8.374324000
H	-0.680710000	-2.278081000	8.117925000
H	0.225355000	-1.219252000	9.236345000
N	-1.233211000	1.228436000	4.450500000
H	-2.044996000	1.847438000	4.426546000
H	-0.827660000	1.234047000	3.512522000
H	-0.518123000	1.654539000	5.110742000
O	0.349269000	3.418381000	5.467531000
O	0.735958000	1.756577000	6.162134000
H	0.607596000	2.120843000	7.053529000
H	1.263395000	3.645500000	5.232554000

TS-Te-3 **$C_{10}H_{24}Te_2S_2O_4$**

-7.54688636 Nimag=1 ν=-161.9

C	-0.633193000	1.823166000	1.357953000
H	0.394062000	1.576846000	1.053674000
H	-1.293089000	1.008554000	1.015184000
C	-0.677903000	1.943272000	2.884626000
H	-0.028160000	2.760181000	3.199006000
C	-2.089559000	2.199607000	3.398715000
H	-2.481485000	3.113301000	2.948381000
H	-2.769923000	1.372972000	3.177729000
Te	-2.199241000	2.533369000	5.546599000
C	-3.751233000	4.018079000	5.426172000
H	-3.328772000	4.890199000	4.926852000
H	-4.609582000	3.614770000	4.887710000
H	-4.035075000	4.268144000	6.449692000
O	-1.032969000	3.064946000	0.795980000
C	-0.888860000	3.105736000	-0.613439000
H	0.157631000	2.953405000	-0.919179000
H	-1.513731000	2.346816000	-1.110630000
H	-1.211949000	4.098642000	-0.936488000
S	-0.035639000	0.401873000	3.686940000
S	1.991629000	0.465006000	3.494550000
C	2.485446000	0.687274000	6.221122000
H	1.460391000	0.298639000	6.310838000
H	3.171593000	-0.175654000	6.221796000
C	2.627436000	1.472179000	4.911927000
H	2.020627000	2.377607000	4.969041000
C	4.085095000	1.829707000	4.605787000
H	4.555966000	2.197030000	5.519703000
H	4.651903000	0.953893000	4.276275000
Te	4.464245000	3.376887000	3.116717000
C	4.406679000	5.038502000	4.503633000
H	3.398702000	5.159938000	4.898499000
H	5.138563000	4.875943000	5.295376000
H	4.686086000	5.921035000	3.924102000
O	2.772675000	1.559463000	7.306614000
C	2.680964000	0.918317000	8.565741000
H	1.670230000	0.519865000	8.749756000
H	3.402031000	0.090450000	8.657040000
H	2.908152000	1.671869000	9.324340000
O	-0.617215000	4.177815000	5.245349000
O	0.725765000	5.553121000	4.933794000
H	0.770686000	5.338424000	3.990855000
H	-0.074400000	4.073071000	6.040112000

TS-Te-4 **$C_8H_{18}TeSO_3$**

-5.68097770 Nimag=1 ν=-180.4

S	2.508865000	-0.778468000	0.875520000
C	0.414751000	-2.124436000	-0.263570000
H	1.124710000	-2.459267000	-1.032218000
H	0.470684000	-2.834181000	0.579117000
C	0.799187000	-0.715271000	0.187229000
H	0.835584000	-0.065572000	-0.687864000
C	-0.121289000	-0.102461000	1.239822000
H	-0.244535000	-0.753929000	2.108125000
H	0.272809000	0.858321000	1.571656000
Te	-2.135764000	0.404704000	0.544585000
C	-2.332225000	2.109051000	1.853849000
H	-1.608459000	2.866982000	1.553294000
H	-3.347565000	2.492793000	1.738808000
H	-2.171932000	1.797404000	2.887098000
O	-0.908246000	-2.095146000	-0.790136000
C	-1.345765000	-3.350808000	-1.279566000
H	-2.364307000	-3.212595000	-1.651366000
H	-0.709571000	-3.704463000	-2.104947000
H	-1.354512000	-4.116587000	-0.488389000
C	3.351794000	0.575646000	-0.050071000
H	2.711371000	1.461124000	-0.040236000
H	4.223408000	0.785449000	0.581220000
C	3.774086000	0.205825000	-1.435883000
H	4.530408000	-0.576591000	-1.502563000
C	3.292900000	0.761357000	-2.550921000
H	2.517111000	1.522496000	-2.533647000
H	3.657081000	0.448023000	-3.526553000
O	-0.921098000	1.674657000	-1.152582000
O	0.179416000	2.421380000	-2.420131000
H	-1.519430000	1.455216000	-1.881414000

H	0.079640000	3.323553000	-2.084959000

Table S8. COSMO cartesian coordinates.

Cartesian Coordinates (in Å), electronic energies relative to the atomic fragments (in Hartree) and imaginary frequencies (in cm^{-1}) of the compounds investigated (Table 3 in the main text) (level of theory: COSMO-ZORA-OLYP/TZ2P).

H_2O

-0.52411819 Nimag=0

O	0.000000000	0.000000000	-0.398464000
H	0.000000000	0.759715000	0.200426000
H	0.000000000	-0.759715000	0.200426000

H_2O_2

-0.66271840 Nimag=0

O	-0.038767000	-0.728574000	-0.312711000
O	0.038767000	0.728574000	-0.312711000
H	0.774545000	0.875935000	0.306580000
H	-0.774545000	-0.875935000	0.306580000

S-1 **$C_5NH_{13}S_2O_2$**

-4.34478339 Nimag=0

C	-0.937411000	2.777153000	0.926204000
H	-0.610214000	2.875152000	1.966500000
H	-0.527472000	3.618189000	0.360288000
C	-0.459861000	1.450317000	0.320715000
H	-0.806848000	1.379791000	-0.713012000
H	-2.026102000	2.855187000	0.900361000
C	1.075884000	1.390590000	0.323400000
H	1.454856000	2.303668000	-0.143878000
H	1.449605000	1.353842000	1.352519000
S	1.743701000	-0.035360000	-0.602172000
C	3.515558000	0.333569000	-0.505502000
H	4.025169000	-0.472457000	-1.038474000
H	3.747842000	1.286025000	-0.989059000
H	3.857590000	0.346894000	0.532726000
N	-1.009263000	0.274691000	1.027940000
H	-0.940953000	0.303664000	2.040484000
S	-2.295420000	-0.614182000	0.534324000
O	-2.163268000	-0.817750000	-0.900691000
O	-2.330103000	-1.771075000	1.420231000
C	-3.805045000	0.301616000	0.807917000
H	-3.873552000	0.556757000	1.865756000
H	-3.798977000	1.195049000	0.184060000
H	-4.626782000	-0.355605000	0.517772000

S-2 **$C_4NH_{11}S$**

-3.15628700 Nimag=0

C	-2.752031000	-0.680509000	-0.570337000
H	-3.768887000	-0.280345000	-0.643259000
H	-2.227182000	-0.444555000	-1.500028000
C	-2.040435000	-0.089569000	0.648165000
H	-2.630046000	-0.379600000	1.535652000
H	-2.828596000	-1.768350000	-0.480905000
C	-0.654249000	-0.691080000	0.915861000
H	-0.264779000	-0.302038000	1.863363000
H	-0.754448000	-1.776113000	1.015706000
S	0.594455000	-0.357652000	-0.384312000
C	1.943003000	-1.408478000	0.219258000
H	2.277931000	-1.089119000	1.209992000
H	1.641317000	-2.459093000	0.249204000
H	2.766698000	-1.293155000	-0.489173000
N	-1.959705000	1.377830000	0.519804000
H	-1.568059000	1.768037000	1.375207000
H	-2.905278000	1.751470000	0.465833000

S-2-H⁺ **$C_4NH_{12}S^+$**

-3.15594809 Nimag=0

C	-3.198589000	-1.275948000	1.027640000
H	-2.995316000	-2.301235000	1.351227000
H	-3.314681000	-0.651549000	1.915897000
C	-2.064171000	-0.725053000	0.175517000
H	-2.299223000	0.293232000	-0.142983000
H	-4.143271000	-1.259071000	0.477419000
C	-0.709180000	-0.747902000	0.881094000
H	-0.814652000	-0.238332000	1.841395000
H	-0.395422000	-1.778364000	1.074309000
S	0.578021000	0.077562000	-0.123441000
C	2.076576000	-0.742716000	0.482031000
H	2.035461000	-1.818917000	0.300023000
H	2.903018000	-0.311088000	-0.086864000
H	2.228044000	-0.540068000	1.544545000
N	-1.962430000	-1.516602000	-1.109329000
H	-2.770144000	-1.358631000	-1.710814000
H	-1.900511000	-2.518533000	-0.924722000
H	-1.118702000	-1.237385000	-1.619548000

S-3 **$C_{10}H_{22}S_4O_2$**

-7.05111031 Nimag=0

C	-0.771991000	1.266165000	1.768504000
H	0.249935000	0.950969000	1.517249000
H	-1.449135000	0.428283000	1.541355000
C	-0.848734000	1.617715000	3.256835000
H	-0.253096000	2.514051000	3.441918000
C	-2.296311000	1.825143000	3.733252000
H	-2.847389000	2.367453000	2.960197000
H	-2.799976000	0.861331000	3.861279000
S	-2.535416000	2.697972000	5.311957000
C	-2.302585000	4.431850000	4.832401000
H	-1.283431000	4.628861000	4.492336000
H	-3.019808000	4.724081000	4.061192000
H	-2.490381000	5.017874000	5.735322000

O	-1.126197000	2.414092000	0.999683000
C	-1.110259000	2.152186000	-0.401666000
H	-0.111683000	1.840478000	-0.740679000
H	-1.834599000	1.370147000	-0.671170000
H	-1.384430000	3.083377000	-0.903109000
S	-0.104547000	0.253176000	4.264409000
S	1.913685000	0.443529000	4.064007000
C	2.486270000	0.797004000	6.756444000
H	1.534405000	0.265368000	6.891335000
H	3.292822000	0.047663000	6.744600000
C	2.460826000	1.565660000	5.432342000
H	1.724725000	2.369346000	5.501506000
C	3.841422000	2.130360000	5.057588000
H	4.323488000	2.513557000	5.960990000
H	4.487699000	1.336726000	4.668471000
S	3.886115000	3.441565000	3.796460000
C	3.413737000	4.909212000	4.752261000
H	2.391412000	4.838720000	5.130427000
H	4.109677000	5.079180000	5.577692000
H	3.473078000	5.749432000	4.056235000
O	2.680419000	1.719385000	7.826906000
C	2.749494000	1.070396000	9.094505000
H	1.823021000	0.520769000	9.315415000
H	3.595207000	0.369273000	9.140054000
H	2.890337000	1.851652000	9.845197000

S-4 **$C_8H_{16}S_2O$**

-5.10945795 Nimag=0

S	2.283619000	-1.395629000	2.025043000
C	-0.098145000	-2.258584000	1.051675000
H	0.517116000	-3.126161000	0.782818000
H	-0.484371000	-2.417650000	2.064674000
C	0.782018000	-0.990440000	1.031877000
H	1.133691000	-0.809841000	0.012616000
C	0.120374000	0.273839000	1.595644000
H	-0.318834000	0.063621000	2.576186000
H	0.877987000	1.049454000	1.724663000
S	-1.163089000	1.007698000	0.501471000
C	-1.212176000	2.692814000	1.171142000
H	-0.241383000	3.186001000	1.071783000
H	-1.951923000	3.235676000	0.577684000
H	-1.527144000	2.693198000	2.217954000
O	-1.243963000	-2.191487000	0.206083000
C	-0.991753000	-2.543515000	-1.154269000
H	-0.300007000	-1.847997000	-1.645601000
H	-0.586097000	-3.562039000	-1.229822000
H	-1.953928000	-2.502228000	-1.670387000
C	3.582484000	-0.336347000	1.270906000
H	3.269107000	0.710265000	1.304561000
H	4.412342000	-0.458475000	1.976377000
C	3.989978000	-0.757849000	-0.103425000
H	4.410949000	-1.760171000	-0.185159000
C	3.871137000	-0.001804000	-1.198827000
H	3.455048000	1.003072000	-1.161422000
H	4.196861000	-0.363619000	-2.170843000

Se-1 **$C_5NH_{13}SeSO_2$**

-4.31009161 Nimag=0

C	-0.964994000	2.760950000	0.923093000
H	-0.687244000	2.849626000	1.978378000
H	-0.516507000	3.598769000	0.381986000
C	-0.474541000	1.430272000	0.333673000
H	-0.789588000	1.360687000	-0.710161000
H	-2.049918000	2.855297000	0.843935000
C	1.055617000	1.362095000	0.387259000
H	1.466711000	2.227623000	-0.135654000
H	1.409682000	1.376960000	1.421736000
Se	1.806979000	-0.245756000	-0.494677000
C	3.671877000	0.366590000	-0.551767000
H	4.237887000	-0.442111000	-1.017713000
H	3.755448000	1.272133000	-1.154468000
H	4.042591000	0.539732000	0.459655000
N	-1.050175000	0.253826000	1.019985000
H	-0.971902000	0.257029000	2.032324000
S	-2.359097000	-0.596640000	0.517803000
O	-2.246214000	-0.764011000	-0.923556000
O	-2.405590000	-1.776363000	1.372547000
C	-3.849457000	0.336927000	0.833967000
H	-3.898163000	0.569971000	1.898013000
H	-3.838846000	1.243406000	0.229530000
H	-4.685622000	-0.301287000	0.542522000

Se-2 **$C_4NH_{11}Se$**

-3.12348914 Nimag=0

C	-3.380068000	-0.841323000	0.052140000
H	-3.359845000	-1.818981000	0.546296000
H	-4.235801000	-0.283348000	0.446761000
C	-2.078408000	-0.059395000	0.303859000
H	-1.964549000	0.059601000	1.388855000
H	-3.543478000	-1.002158000	-1.019551000
C	-0.876209000	-0.850292000	-0.209273000
H	-0.834701000	-1.827771000	0.274770000
H	-0.935217000	-0.997942000	-1.292120000
Se	0.846028000	0.050020000	0.187452000
C	2.003914000	-1.526860000	-0.008525000
H	1.731592000	-2.290411000	0.721993000
H	1.933908000	-1.923235000	-1.022786000
H	3.022031000	-1.181160000	0.180508000
N	-2.075448000	1.301619000	-0.262878000

H	-2.883891000	1.807256000	0.094031000
H	-2.220064000	1.245550000	-1.270561000

Se-2-H+ **$C4NH12Se^+$**

-3.12368589 Nimag=0

C	-3.549908000	0.389875000	0.546688000
H	-4.351089000	0.544324000	-0.181494000
H	-3.757440000	-0.522678000	1.113628000
C	-2.192168000	0.322502000	-0.141508000
H	-2.027918000	1.232639000	-0.721400000
H	-3.557581000	1.233716000	1.240019000
C	-1.033353000	0.107212000	0.830386000
H	-1.009941000	0.931483000	1.544423000
H	-1.158793000	-0.823023000	1.391659000
Se	0.756102000	-0.043652000	0.009278000
C	1.070240000	1.846057000	-0.427751000
H	1.007343000	2.447231000	0.480045000
H	2.085140000	1.882737000	-0.827618000
H	0.366870000	2.198386000	-1.181699000
N	-2.193989000	-0.787474000	-1.164418000
H	-2.458627000	-1.681209000	-0.747872000
H	-1.248554000	-0.888048000	-1.552566000
H	-2.841298000	-0.595262000	-1.927952000

Se-3 **$C_{10}H_{22}Se_2S_2O_2$**

-6.98210603 Nimag=0

C	-0.792456000	1.192491000	1.747924000
H	0.221551000	0.863945000	1.480896000
H	-1.480178000	0.353868000	1.558617000
C	-0.831178000	1.582274000	3.229581000
H	-0.208157000	2.466356000	3.380988000
C	-2.258055000	1.832943000	3.729891000
H	-2.817836000	2.383205000	2.971339000
H	-2.788058000	0.892642000	3.907107000
Se	-2.444762000	2.847171000	5.412999000
C	-2.246286000	4.665943000	4.695488000
H	-1.253644000	4.809024000	4.267162000
H	-3.020349000	4.863828000	3.952717000
H	-2.374607000	5.333880000	5.549473000
O	-1.157893000	2.322749000	0.958631000
C	-1.184855000	2.023787000	-0.434985000
H	-0.199632000	1.692870000	-0.794320000
H	-1.924164000	1.242434000	-0.662839000
H	-1.464556000	2.944151000	-0.953154000
S	-0.092005000	0.221600000	4.247082000
S	1.927120000	0.416475000	4.059403000
C	2.514719000	0.728899000	6.749884000
H	1.571826000	0.181003000	6.885035000
H	3.331237000	-0.008504000	6.709957000
C	2.460526000	1.527368000	5.442876000
H	1.701824000	2.307595000	5.534190000
C	3.818465000	2.133519000	5.072133000
H	4.298987000	2.526039000	5.970223000
H	4.487850000	1.379541000	4.647983000
Se	3.804754000	3.602024000	3.753158000
C	3.336305000	5.085288000	4.954242000
H	2.343028000	4.941346000	5.380810000
H	4.085205000	5.182706000	5.741446000
H	3.338632000	5.980561000	4.329432000
O	2.711023000	1.629141000	7.838472000
C	2.814138000	0.952015000	9.088768000
H	1.901202000	0.381666000	9.313619000
H	3.672031000	0.264410000	9.102334000
H	2.956050000	1.717746000	9.855140000

Se-4 **$C_8H_{16}SeSO$**

-5.07729180 Nimag=0

S	2.458087000	0.298067000	1.345031000
C	0.706886000	-1.574895000	0.316175000
H	1.659917000	-2.068181000	0.080033000
H	0.363503000	-1.964854000	1.286559000
C	0.908703000	-0.058782000	0.405263000
H	1.057749000	0.344943000	-0.600474000
C	-0.206944000	0.716894000	1.106537000
H	-0.347096000	0.363806000	2.131576000
H	0.046063000	1.777320000	1.131172000
Se	-1.978942000	0.625085000	0.200191000
C	-2.763635000	2.181575000	1.108770000
H	-2.194468000	3.083466000	0.877554000
H	-3.779113000	2.275385000	0.718625000
H	-2.799176000	2.013914000	2.186436000
O	-0.245575000	-1.867530000	-0.703889000
C	-0.586483000	-3.249221000	-0.752062000
H	0.295203000	-3.874102000	-0.957055000
H	-1.043428000	-3.582796000	0.190789000
H	-1.308203000	-3.372530000	-1.563126000
C	3.764532000	0.183614000	0.056211000
H	4.680258000	0.219455000	0.657528000
H	3.720033000	-0.796481000	-0.425372000
C	3.739777000	1.299837000	-0.936859000
H	3.884451000	2.300018000	-0.527813000
C	3.557326000	1.145926000	-2.251704000
H	3.408079000	0.165739000	-2.700447000
H	3.560332000	1.999912000	-2.924407000

Te-1 **$C_5NH_{13}TeSO_2$**

-4.27695989 Nimag=0

C	-0.028640000	2.194479000	1.000155000
H	0.760922000	1.697253000	1.569442000
H	0.380712000	3.114610000	0.573231000

C	-0.570319000	1.292134000	-0.111452000
H	-1.402012000	1.818996000	-0.592626000
H	-0.829822000	2.463373000	1.693282000
C	0.436641000	0.990384000	-1.233624000
H	-0.061793000	0.468652000	-2.052311000
H	0.852707000	1.919234000	-1.630449000
Te	2.145645000	-0.296230000	-0.782805000
C	3.537414000	1.237840000	-0.150805000
H	3.276013000	1.619054000	0.834750000
H	4.513928000	0.751459000	-0.118262000
H	3.547471000	2.037735000	-0.891544000
N	-1.144870000	0.069750000	0.485696000
H	-0.465421000	-0.631931000	0.766967000
S	-2.539149000	-0.636208000	-0.019335000
O	-2.840658000	-0.298515000	-1.406352000
O	-2.432654000	-2.044105000	0.350055000
C	-3.803652000	0.114635000	0.987142000
H	-3.593777000	-0.108708000	2.032904000
H	-3.801968000	1.190636000	0.808823000
H	-4.755997000	-0.318508000	0.676620000

Te-2 **$C_4NH_{11}Te$**

-3.09135453 Nimag=0

C	-3.441787000	-0.833213000	0.054944000
H	-3.410189000	-1.822618000	0.524506000
H	-4.286193000	-0.284167000	0.485585000
C	-2.130510000	-0.061673000	0.290565000
H	-1.991981000	0.038088000	1.374585000
H	-3.633234000	-0.968333000	-1.015670000
C	-0.938944000	-0.835652000	-0.266855000
H	-0.838467000	-1.804040000	0.226661000
H	-1.039846000	-0.999265000	-1.343461000
Te	0.950954000	0.211181000	0.060081000
C	2.162696000	-1.586459000	0.023385000
H	1.861702000	-2.252030000	0.832971000
H	2.061706000	-2.080248000	-0.943518000
H	3.195773000	-1.265613000	0.169223000
N	-2.136189000	1.310416000	-0.249262000
H	-2.927903000	1.817984000	0.140782000
H	-2.307795000	1.276814000	-1.253551000

Te-2-H^+

-3.09170893 Nimag=0

C	-3.584125000	0.390691000	0.543192000
H	-4.372133000	0.566099000	-0.194916000
H	-3.822133000	-0.516203000	1.107246000
C	-2.218295000	0.287956000	-0.127469000
H	-2.026887000	1.193897000	-0.706095000
H	-3.578213000	1.234190000	1.236848000
C	-1.078118000	0.045416000	0.857131000
H	-1.042216000	0.871859000	1.567796000
H	-1.229349000	-0.878783000	1.421850000
Te	0.919885000	-0.140715000	0.000263000
C	1.176720000	1.957174000	-0.460259000
H	1.068629000	2.541314000	0.453680000
H	2.193313000	2.050318000	-0.845926000
H	0.461947000	2.273105000	-1.218934000
N	-2.244910000	-0.819911000	-1.153467000
H	-2.526165000	-1.709051000	-0.737957000
H	-1.302291000	-0.939785000	-1.546550000
H	-2.890624000	-0.612757000	-1.914585000

Te-3 **$C_{10}H_{22}Te_2S_2O_2$**

-6.91818731 Nimag=0

C	-0.789554000	1.137495000	1.742749000
H	0.229191000	0.825202000	1.473411000
H	-1.463805000	0.286447000	1.560867000
C	-0.829551000	1.535403000	3.222772000
H	-0.207689000	2.421179000	3.370668000
C	-2.253816000	1.776836000	3.728863000
H	-2.815184000	2.345803000	2.985299000
H	-2.787448000	0.836212000	3.889837000
Te	-2.470016000	2.901191000	5.583154000
C	-2.554987000	4.849718000	4.642136000
H	-1.622425000	5.052555000	4.115681000
H	-3.406035000	4.892957000	3.962320000
H	-2.687521000	5.569501000	5.451967000
O	-1.176845000	2.256475000	0.947381000
C	-1.193364000	1.950443000	-0.444740000
H	-0.200257000	1.638914000	-0.799889000
H	-1.915002000	1.152270000	-0.671177000
H	-1.491144000	2.862027000	-0.968413000
S	-0.083216000	0.179544000	4.241621000
S	1.936153000	0.375421000	4.049106000
C	2.524325000	0.674644000	6.737758000
H	1.573573000	0.140759000	6.875194000
H	3.329563000	-0.074579000	6.690475000
C	2.476210000	1.480722000	5.434554000
H	1.719898000	2.263366000	5.527890000
C	3.835846000	2.076036000	5.060740000
H	4.311946000	2.489520000	5.951873000
H	4.509066000	1.315999000	4.655021000
Te	3.842046000	3.700944000	3.608632000
C	3.603388000	5.287921000	5.062486000
H	2.653062000	5.178296000	5.584794000
H	4.438989000	5.273975000	5.762405000
H	3.608507000	6.219778000	4.494088000
O	2.738962000	1.565944000	7.830470000
C	2.825165000	0.881061000	9.077618000

H	1.899864000	0.329133000	9.298175000
H	3.668092000	0.175083000	9.089801000
H	2.981814000	1.639806000	9.848115000

Te-4 $C_8H_{16}TeSO$

-5.04593814 Nimag=0

S	2.495763000	0.249729000	1.345410000
C	0.761444000	-1.623812000	0.297562000
H	1.704170000	-2.096482000	-0.010598000
H	0.484128000	-2.040703000	1.277581000
C	0.941676000	-0.107475000	0.409130000
H	1.082629000	0.314882000	-0.589906000
C	-0.187111000	0.627873000	1.125192000
H	-0.324037000	0.252563000	2.141837000
H	0.031141000	1.695299000	1.169457000
Te	-2.149466000	0.533405000	0.141130000
C	-2.863981000	2.343293000	1.095944000
H	-2.240967000	3.188755000	0.802312000
H	-3.885684000	2.492932000	0.741351000
H	-2.863512000	2.217001000	2.179066000
O	-0.250870000	-1.906335000	-0.667260000
C	-0.582940000	-3.290495000	-0.726081000
H	0.289098000	-3.899837000	-1.004125000
H	-0.973202000	-3.650322000	0.236579000
H	-1.355606000	-3.401739000	-1.490433000
C	3.797676000	0.180798000	0.047730000
H	4.716132000	0.219255000	0.644742000
H	3.765960000	-0.789950000	-0.453264000
C	3.749954000	1.315947000	-0.922712000
H	3.881545000	2.309938000	-0.494540000
C	3.561753000	1.185692000	-2.239330000
H	3.424865000	0.212460000	-2.706797000
H	3.547280000	2.052910000	-2.894737000

TS-S-1 $C_5NH_{15}S_2O_4$

-4.98818787 Nimag=1 ν=-301.9

C	-0.866462000	3.255442000	0.870613000
H	-0.599225000	3.494598000	1.904858000
H	-0.416672000	4.008947000	0.218536000
C	-0.369875000	1.857658000	0.479516000
H	-0.640783000	1.659011000	-0.560224000
H	-1.950768000	3.329041000	0.769557000
C	1.161053000	1.790580000	0.607549000
H	1.600250000	2.672494000	0.132697000
H	1.468484000	1.774439000	1.657508000
S	1.887401000	0.353357000	-0.242639000
C	3.638322000	0.675204000	0.019091000
H	4.174531000	-0.174947000	-0.404819000
H	3.920830000	1.588724000	-0.507623000
H	3.854649000	0.759755000	1.085470000
N	-0.980163000	0.784193000	1.291404000
H	-1.023481000	0.973711000	2.288382000
S	-2.240042000	-0.158867000	0.809187000
O	-1.947667000	-0.609227000	-0.542946000
O	-2.405732000	-1.141623000	1.870911000
C	-3.736350000	0.812366000	0.740921000
H	-3.915099000	1.253422000	1.721715000
H	-3.631676000	1.577771000	-0.027654000
H	-4.540108000	0.122098000	0.478269000
O	1.941048000	-1.437328000	1.108511000
O	1.971453000	-2.967677000	2.125412000
H	0.973657000	-1.443078000	1.173372000
H	2.546984000	-2.581053000	2.801449000

TS-S-2 $C_4NH_{13}SO_2$

-3.80372622 Nimag=1 ν=-311.5

C	-2.774902000	-0.636322000	-0.870615000
H	-3.729991000	-0.102058000	-0.886708000
H	-2.126299000	-0.190456000	-1.630507000
C	-2.157516000	-0.563079000	0.525915000
H	-2.889693000	-0.990366000	1.228476000
H	-2.968220000	-1.675712000	-1.149868000
C	-0.906244000	-1.439479000	0.700071000
H	-0.521718000	-1.350603000	1.720333000
H	-1.153223000	-2.489015000	0.513866000
S	0.450533000	-0.987557000	-0.431740000
C	1.815282000	-1.927546000	0.267634000
H	1.973544000	-1.649278000	1.311024000
H	1.608909000	-2.996441000	0.178617000
H	2.697247000	-1.676335000	-0.324488000
N	-1.840116000	0.836391000	0.881889000
H	-1.830591000	0.935170000	1.894549000
H	-2.584766000	1.446617000	0.555472000
O	1.036601000	0.993023000	0.469395000
O	1.391427000	2.579931000	1.315915000
H	0.065089000	1.153500000	0.569079000
H	1.940010000	2.914232000	0.591746000

TS-S-2-H$^+$ $C_4NH_{14}SO_2^+$

-3.80088008 Nimag=1 ν=-279.9

C	-3.138341000	-0.798254000	1.416897000
H	-3.042791000	-1.778514000	1.893680000
H	-3.177990000	-0.035717000	2.197180000
C	-1.964477000	-0.514296000	0.488669000
H	-2.080274000	0.475575000	0.041922000
H	-4.080252000	-0.766200000	0.862869000
C	-0.614245000	-0.608298000	1.202585000
H	-0.684345000	-0.089904000	2.161270000
H	-0.333123000	-1.647765000	1.393581000
S	0.727926000	0.203340000	0.276320000

C	2.174332000	-0.441190000	1.127586000
H	2.198369000	-1.530566000	1.070979000
H	3.041018000	-0.014723000	0.620336000
H	2.154996000	-0.105888000	2.167156000
N	-1.986062000	-1.478997000	-0.673137000
H	-2.749745000	-1.263137000	-1.312082000
H	-2.118720000	-2.437457000	-0.349597000
H	-1.079071000	-1.433631000	-1.191997000
O	0.633099000	-1.223930000	-1.466137000
O	0.450271000	-2.337376000	-2.933865000
H	0.804501000	-0.494747000	-2.083647000
H	1.026590000	-3.019958000	-2.558534000

TS-S-3 **$C_{10}H_{24}S_4O_4$**

-7.69546157 Nimag=1 ν=-302.0

C	-0.772600000	0.574800000	2.951900000
H	0.263200000	0.250900000	2.781200000
H	-1.409000000	-0.323600000	2.965200000
C	-0.867500000	1.307500000	4.293200000
H	-0.313800000	2.246400000	4.226500000
C	-2.323400000	1.570600000	4.714200000
H	-2.899200000	1.857000000	3.830100000
H	-2.781100000	0.656000000	5.105300000
S	-2.600200000	2.829400000	5.998700000
C	-2.472000000	4.373700000	5.055700000
H	-1.469900000	4.521400000	4.646900000
H	-3.213900000	4.404800000	4.253800000
H	-2.681800000	5.175400000	5.768000000
O	-1.180700000	1.458500000	1.910000000
C	-1.154900000	0.833100000	0.628900000
H	-0.143700000	0.485800000	0.371900000
H	-1.843500000	-0.023000000	0.587300000
H	-1.471700000	1.584100000	-0.098600000
S	-0.066400000	0.293900000	5.620500000
S	1.941500000	0.515100000	5.362000000
C	2.487900000	1.557900000	7.880300000
H	1.559200000	1.037400000	8.150900000
H	3.325400000	0.862500000	8.043100000
C	2.423200000	1.969700000	6.406200000
H	1.644700000	2.726000000	6.285200000
C	3.776900000	2.495000000	5.906700000
H	4.208800000	3.165500000	6.652900000
H	4.483300000	1.673700000	5.755300000
S	3.754600000	3.378400000	4.320700000
C	3.167900000	5.014800000	4.789900000
H	2.138600000	4.961200000	5.149100000
H	3.819500000	5.447500000	5.551100000
H	3.200100000	5.618600000	3.881600000
O	2.644000000	2.724900000	8.682300000
C	2.742600000	2.417600000	10.071900000
H	1.840300000	1.904400000	10.433800000
H	3.617300000	1.785200000	10.280300000
H	2.851700000	3.367500000	10.600300000
O	5.925900000	3.946300000	4.361600000
O	7.701500000	4.384500000	4.364600000
H	6.083000000	3.400400000	3.575500000
H	7.885300000	3.877700000	5.169400000

TS-S-4 **$C_8H_{18}S_2O_3$**

-5.75586239 Nimag=1 ν=-308.9

S	2.414045000	-1.334811000	2.035935000
C	-0.010311000	-2.237583000	1.210767000
H	0.579408000	-3.107152000	0.899888000
H	-0.316099000	-2.388176000	2.251484000
C	0.865646000	-0.971243000	1.107198000
H	1.165001000	-0.820894000	0.066549000
C	0.226169000	0.307273000	1.666317000
H	-0.205690000	0.131545000	2.655911000
H	0.979494000	1.093186000	1.755393000
S	-1.073692000	1.015954000	0.589080000
C	-1.348190000	2.597467000	1.403522000
H	-0.441545000	3.202781000	1.340484000
H	-2.155077000	3.088739000	0.857042000
H	-1.641183000	2.445956000	2.443961000
O	-1.220495000	-2.174083000	0.454525000
C	-1.085127000	-2.567832000	-0.914666000
H	-0.419799000	-1.900793000	-1.475744000
H	-0.707965000	-3.595998000	-0.987605000
H	-2.084419000	-2.518681000	-1.352366000
C	3.668540000	-0.312084000	1.163959000
H	3.365701000	0.737937000	1.184144000
H	4.539852000	-0.420246000	1.819864000
C	3.982665000	-0.781799000	-0.218908000
H	4.393283000	-1.788728000	-0.295020000
C	3.793782000	-0.060738000	-1.327969000
H	3.385441000	0.947492000	-1.295639000
H	4.052955000	-0.455513000	-2.307088000
O	-2.985115000	0.105004000	1.386817000
O	-4.474218000	-0.715909000	2.033608000
H	-2.648796000	-0.752060000	1.070019000
H	-5.060515000	-0.326769000	1.367992000

TS-Se-1 **$C_5NH_{15}SeSO_4$**

-4.95970219 Nimag=1 ν=-284.9

C	-1.023316000	3.188436000	0.980611000
H	-0.819327000	3.297064000	2.050622000
H	-0.561668000	4.031052000	0.458699000
C	-0.461691000	1.863660000	0.445316000
H	-0.700510000	1.774156000	-0.616966000

H	-2.102506000	3.250440000	0.827535000
C	1.062743000	1.840857000	0.605878000
H	1.496713000	2.698543000	0.088567000
H	1.354523000	1.875771000	1.658478000
Se	1.908298000	0.238791000	-0.180319000
C	3.748744000	0.888206000	-0.143442000
H	4.365244000	0.066278000	-0.509097000
H	3.838901000	1.750339000	-0.804767000
H	4.025596000	1.143893000	0.879375000
N	-1.051556000	0.682317000	1.108886000
H	-1.090618000	0.726667000	2.122523000
S	-2.272049000	-0.232557000	0.498724000
O	-1.980200000	-0.464505000	-0.907881000
O	-2.389909000	-1.369124000	1.401983000
C	-3.808506000	0.674126000	0.584942000
H	-3.994927000	0.951388000	1.622721000
H	-3.741671000	1.553062000	-0.055637000
H	-4.587571000	0.001510000	0.221670000
O	2.357330000	-1.069975000	1.878613000
O	2.645564000	-1.980745000	3.341947000
H	1.477463000	-1.459811000	1.756499000
H	3.299355000	-2.576917000	2.945927000

TS-Se-2 **$C_4NH_{13}SeO_2$**

-3.77608792 Nimag=1 ν=-294.8

C	-2.856631000	-2.047065000	2.258460000
H	-3.344551000	-1.774297000	3.199739000
H	-3.618004000	-2.064686000	1.470829000
C	-1.742905000	-1.038295000	1.933110000
H	-1.006768000	-1.083772000	2.743490000
H	-2.452979000	-3.059044000	2.369066000
C	-1.051132000	-1.425930000	0.622516000
H	-0.701095000	-2.459654000	0.654063000
H	-1.733803000	-1.323054000	-0.226520000
Se	0.511189000	-0.340602000	0.088475000
C	1.856101000	-1.086426000	1.293570000
H	2.779612000	-0.548116000	1.075809000
H	1.561790000	-0.920952000	2.329152000
H	1.980589000	-2.148699000	1.079750000
N	-2.197630000	0.361511000	1.865982000
H	-2.627550000	0.619374000	2.750903000
H	-2.930314000	0.453950000	1.164176000
O	0.286313000	1.666760000	1.388135000
O	0.114950000	3.083306000	2.475128000
H	-0.634782000	1.339504000	1.587639000
H	0.568586000	2.647264000	3.211070000

TS-Se-2-H⁺ **$C_4NH_{14}SeO_2^+$**

-3.77301023 Nimag=1 ν=-260.4

C	-3.856083000	0.814645000	0.668941000
H	-4.678582000	0.996573000	-0.028522000
H	-4.040850000	-0.126758000	1.194823000
C	-2.523394000	0.787155000	-0.072561000
H	-2.383221000	1.732278000	-0.599933000
H	-3.849409000	1.624328000	1.401462000
C	-1.348149000	0.527708000	0.867521000
H	-1.367248000	1.262493000	1.673966000
H	-1.405436000	-0.467845000	1.314872000
Se	0.479128000	0.610245000	0.121146000
C	0.596512000	2.521456000	-0.256569000
H	0.423380000	3.069250000	0.670403000
H	1.615762000	2.685479000	-0.607808000
H	-0.118775000	2.806352000	-1.026883000
N	-2.559118000	-0.260662000	-1.153221000
H	-2.741410000	-1.187424000	-0.767744000
H	-1.638307000	-0.281244000	-1.657335000
H	-3.297113000	-0.059365000	-1.826802000
O	-0.039060000	-0.003277000	-2.200950000
O	-0.434930000	-0.524754000	-3.868951000
H	0.515094000	-0.790167000	-2.071658000
H	-0.162874000	0.314580000	-4.270906000

TS-Se-3 **$C_{10}H_{24}Se_2S_2O_4$**

-7.63158730 Nimag=1 ν=-274.0

C	-0.804100000	0.486100000	2.973200000
H	0.218800000	0.116400000	2.817200000
H	-1.475400000	-0.385400000	3.015500000
C	-0.871400000	1.268300000	4.289500000
H	-0.267700000	2.173500000	4.195900000
C	-2.309100000	1.617200000	4.690300000
H	-2.867900000	1.926400000	3.805000000
H	-2.824900000	0.751000000	5.114500000
Se	-2.534100000	3.050700000	6.028600000
C	-2.363900000	4.606300000	4.839700000
H	-1.371000000	4.646500000	4.390500000
H	-3.135800000	4.577100000	4.069300000
H	-2.511200000	5.480200000	5.477300000
O	-1.175200000	1.348400000	1.899900000
C	-1.170400000	0.678500000	0.641200000
H	-0.172700000	0.284000000	0.399900000
H	-1.891100000	-0.151700000	0.626600000
H	-1.456400000	1.415500000	-0.112900000
S	-0.122500000	0.257200000	5.649700000
S	1.894400000	0.430700000	5.426300000
C	2.493300000	1.460400000	7.932100000
H	1.571200000	0.938000000	8.222600000
H	3.334200000	0.763000000	8.066700000
C	2.390200000	1.886300000	6.463100000
H	1.596200000	2.630300000	6.367600000

C	3.716900000	2.431600000	5.928100000
H	4.165600000	3.109800000	6.655400000
H	4.432500000	1.629000000	5.731600000
Se	3.608900000	3.428900000	4.231600000
C	3.095000000	5.177700000	4.934300000
H	2.116100000	5.113300000	5.409800000
H	3.852400000	5.522700000	5.637900000
H	3.045800000	5.842000000	4.070600000
O	2.671600000	2.619300000	8.741700000
C	2.802600000	2.298100000	10.125100000
H	1.907800000	1.783300000	10.503800000
H	3.680200000	1.661400000	10.306700000
H	2.926800000	3.242200000	10.660500000
O	5.971900000	4.082400000	4.238500000
O	7.670600000	4.548900000	4.224600000
H	6.107700000	3.439200000	3.524600000
H	7.875100000	4.123600000	5.071100000

TS-Se-4 **$C_8H_{18}SeSO_3$**

-5.72575421 Nimag=1 ν=-286.6

S	2.313727000	-1.367694000	2.031415000
C	-0.053850000	-2.226739000	1.026015000
H	0.539016000	-3.096861000	0.722226000
H	-0.409878000	-2.398970000	2.047486000
C	0.839417000	-0.969112000	0.995210000
H	1.213839000	-0.814538000	-0.020261000
C	0.186268000	0.306124000	1.527807000
H	-0.236825000	0.151412000	2.522557000
H	0.913076000	1.117973000	1.572407000
Se	-1.281538000	1.021357000	0.391229000
C	-1.444146000	2.758781000	1.268991000
H	-0.512312000	3.312681000	1.148416000
H	-2.258064000	3.278715000	0.761602000
H	-1.683988000	2.616875000	2.323048000
O	-1.225260000	-2.121939000	0.216251000
C	-1.038699000	-2.512411000	-1.147016000
H	-0.309090000	-1.878904000	-1.665904000
H	-0.713910000	-3.559226000	-1.212274000
H	-2.008834000	-2.405560000	-1.637295000
C	3.651272000	-0.364210000	1.269287000
H	3.360717000	0.689291000	1.259215000
H	4.460905000	-0.479536000	1.998991000
C	4.079952000	-0.844783000	-0.078612000
H	4.476874000	-1.859479000	-0.114500000
C	4.007111000	-0.124670000	-1.201798000
H	3.616872000	0.891193000	-1.208770000
H	4.347115000	-0.528145000	-2.152201000
O	-3.114772000	-0.003361000	1.715439000
O	-4.278154000	-0.860041000	2.701229000
H	-2.842626000	-0.811752000	1.250393000
H	-5.060469000	-0.474228000	2.278874000

TS-Te-1 **$C_5NH_{15}TeSO_4$**

-4.93214191 Nimag=1 ν=-289.9

C	-0.691450000	-2.397026000	-0.624549000
H	-0.609023000	-1.582086000	0.100764000
H	-1.259168000	-2.032056000	-1.484786000
C	0.686272000	-2.883925000	-1.074918000
H	0.544148000	-3.725663000	-1.761821000
H	-1.260287000	-3.207696000	-0.163084000
C	1.496678000	-1.853654000	-1.874543000
H	2.423777000	-2.304915000	-2.230261000
H	0.928608000	-1.509288000	-2.741503000
Te	2.161461000	-0.048255000	-0.847746000
C	0.633667000	1.278075000	-1.590088000
H	-0.331124000	1.012504000	-1.160191000
H	0.924721000	2.279965000	-1.270852000
H	0.612333000	1.220361000	-2.678093000
N	1.478917000	-3.396172000	0.066521000
H	1.290338000	-2.959866000	0.962597000
S	1.933048000	-4.961910000	0.239594000
O	2.462452000	-5.409121000	-1.040708000
O	2.790932000	-4.993882000	1.417030000
C	0.491152000	-5.953734000	0.597668000
H	0.040480000	-5.592400000	1.522295000
H	-0.206970000	-5.882559000	-0.236720000
H	0.835933000	-6.982765000	0.713898000
O	3.646543000	0.537919000	-3.116625000
O	4.564920000	0.814378000	-4.488272000
H	4.397673000	0.376954000	-2.522335000
H	4.447199000	1.777001000	-4.508517000

TS-Te-2 **$C_4NH_{13}TeO_2$**

-3.74807938 Nimag=1 ν=-304.9

C	-3.640480000	-0.621132000	0.330288000
H	-3.802234000	-1.253588000	-0.549586000
H	-3.820203000	-1.225238000	1.225880000
C	-2.220087000	-0.031494000	0.338316000
H	-2.117903000	0.571538000	1.248856000
H	-4.382290000	0.184391000	0.314349000
C	-1.181828000	-1.154769000	0.391155000
H	-1.340537000	-1.772523000	1.276475000
H	-1.239993000	-1.800808000	-0.489500000
Te	0.908342000	-0.539711000	0.507751000
C	1.433136000	-1.055661000	-1.520991000
H	0.859866000	-0.446009000	-2.218586000
H	2.499472000	-0.849158000	-1.624048000
H	1.240397000	-2.117691000	-1.675722000
N	-1.945114000	0.879739000	-0.787265000

H	-2.675939000	1.585829000	-0.834491000
H	-1.998766000	0.370546000	-1.667723000
O	0.416840000	2.131851000	0.210744000
O	-0.062002000	3.764012000	0.047396000
H	-0.447385000	3.837434000	0.933843000
H	-0.390023000	1.753442000	-0.240089000

TS-Te-2-H$^+$ **$C_4NH_{14}TeO_2^+$**

-3.74595698 Nimag=1 ν=-287.1

C	-3.218486000	-1.309987000	1.268929000
H	-2.913652000	-2.306249000	1.603254000
H	-3.491242000	-0.720574000	2.146679000
C	-2.093419000	-0.605492000	0.515525000
H	-2.442535000	0.372061000	0.174843000
H	-4.106470000	-1.405715000	0.637244000
C	-0.833118000	-0.446886000	1.360142000
H	-1.100538000	0.067503000	2.283263000
H	-0.396269000	-1.413160000	1.623841000
Te	0.786014000	0.746675000	0.500210000
C	2.264146000	-0.819198000	0.520283000
H	1.940600000	-1.641896000	-0.115992000
H	3.178460000	-0.371624000	0.128337000
H	2.415011000	-1.145098000	1.549125000
N	-1.783860000	-1.366239000	-0.749547000
H	-2.620406000	-1.464152000	-1.323957000
H	-1.448426000	-2.307328000	-0.542160000
H	-1.056785000	-0.867642000	-1.315668000
O	0.203796000	0.003404000	-2.163751000
O	-0.204983000	-0.405104000	-3.736073000
H	0.700662000	-0.582074000	-4.037784000
H	-0.009110000	0.948656000	-2.241524000

TS-Te-3 **$C_{10}H_{24}Te_2S_2O_4$**

-7.57205253 Nimag=1 ν=-286.1

C	-1.365410000	1.815787000	3.967204000
H	-0.473709000	1.173848000	3.992558000
H	-2.225023000	1.214207000	4.300456000
C	-1.164025000	3.012596000	4.904055000
H	-0.355807000	3.636165000	4.515312000
C	-2.441547000	3.834079000	5.094098000
H	-2.942982000	3.958157000	4.132406000
H	-3.144509000	3.333630000	5.765511000
Te	-2.212908000	5.853346000	5.880590000
C	-1.950451000	6.806049000	3.953966000
H	-1.049966000	6.429900000	3.468631000
H	-2.830890000	6.632226000	3.335182000
H	-1.843098000	7.872864000	4.158477000
O	-1.577709000	2.294236000	2.640606000
C	-1.802328000	1.235259000	1.713056000
H	-0.942208000	0.551525000	1.667610000
H	-2.699589000	0.656422000	1.975420000
H	-1.946360000	1.695438000	0.732581000
S	-0.622799000	2.407088000	6.569522000
S	1.357514000	1.961788000	6.389865000
C	2.312478000	3.662651000	8.359489000
H	1.296635000	3.558617000	8.765577000
H	2.936326000	2.869335000	8.799051000
C	2.268064000	3.514331000	6.833797000
H	1.697562000	4.347038000	6.415757000
C	3.666718000	3.441304000	6.218134000
H	4.305990000	4.208269000	6.659203000
H	4.137919000	2.471542000	6.397645000
Te	3.799483000	3.744569000	4.067938000
C	3.947535000	5.897098000	4.167737000
H	3.023437000	6.305100000	4.577731000
H	4.804513000	6.177494000	4.779847000
H	4.085441000	6.238858000	3.141042000
O	2.836025000	4.947281000	8.687465000
C	2.929972000	5.149676000	10.095809000
H	1.944463000	5.078751000	10.578340000
H	3.604736000	4.417088000	10.561466000
H	3.331701000	6.154383000	10.246995000
O	6.359091000	2.764467000	3.905932000
O	7.951986000	2.162223000	3.875778000
H	6.598877000	3.614185000	4.310115000
H	7.842566000	1.556834000	4.625636000

TS-Te-4 **$C_8H_{18}TeSO_3$**

-5.70088454 Nimag=1 ν=-290.9

S	2.345295000	0.658393000	1.791198000
C	0.892873000	-1.488822000	0.826288000
H	1.898130000	-1.892304000	0.644198000
H	0.568145000	-1.836275000	1.818543000
C	0.920787000	0.041127000	0.790344000
H	1.101033000	0.373649000	-0.235752000
C	-0.327906000	0.723645000	1.341376000
H	-0.501404000	0.457692000	2.386945000
H	-0.233764000	1.807061000	1.261828000
Te	-2.195047000	0.275143000	0.288908000
C	-3.227651000	1.969073000	1.135044000
H	-2.714296000	2.886804000	0.848240000
H	-4.236692000	1.954349000	0.719712000
H	-3.268985000	1.862697000	2.219377000
O	-0.000075000	-1.957446000	-0.183222000
C	-0.205072000	-3.366757000	-0.127098000
H	0.737801000	-3.913412000	-0.270970000
H	-0.646988000	-3.669371000	0.832721000
H	-0.893906000	-3.621669000	-0.935771000
C	3.760106000	0.558140000	0.619981000

H	4.612712000	0.763167000	1.277566000
H	3.861194000	-0.466209000	0.252992000
C	3.699968000	1.551309000	-0.494560000
H	3.704953000	2.599563000	-0.195032000
C	3.642317000	1.236921000	-1.792076000
H	3.633700000	0.203448000	-2.133114000
H	3.610578000	2.008078000	-2.557657000
O	-1.626747000	1.989805000	-1.864979000
O	-1.248429000	2.987653000	-3.139981000
H	-1.363559000	1.160066000	-2.295167000
H	-2.160244000	3.161921000	-3.421831000

Table S9. SAPE cartesian coordinates.

Cartesian Coordinates (in Å), electronic energies relative to the atomic fragments (in Hartree) and imaginary frequencies (in cm^{-1}) of the compounds investigated (Table 4 in the main text) (level of theory: ZORA-OLYP/TZ2P).

S-1-RC(SAPE)

-6.03246141 Nimag=0

C	0.558256000	-0.290137000	5.068777000
H	-0.055264000	-0.309915000	4.161704000
H	0.119363000	-0.985902000	5.791322000
C	2.014718000	-0.670330000	4.760376000
H	2.590737000	-0.616337000	5.688027000
H	0.515459000	0.714221000	5.493592000
C	2.092490000	-2.112213000	4.232141000
H	1.674034000	-2.780318000	4.990891000
H	1.493884000	-2.219963000	3.320655000
S	3.805140000	-2.653027000	3.894911000
C	3.560729000	-4.445009000	3.780473000
H	3.203061000	-4.855575000	4.728692000
H	2.872526000	-4.705385000	2.972166000
H	4.540735000	-4.872926000	3.556189000
N	2.661484000	0.250630000	3.814551000
H	2.188347000	0.377919000	2.924040000
S	3.384170000	1.672510000	4.282018000
O	3.116981000	2.627312000	3.223825000
O	3.054851000	1.961062000	5.663860000
C	5.136565000	1.299752000	4.233816000
H	5.412650000	1.017612000	3.217637000
H	5.653175000	2.213840000	4.533448000
H	5.353036000	0.494571000	4.936327000
H	4.051145000	-2.096926000	1.502988000
O	4.090011000	-1.732832000	0.592930000
O	3.851650000	-2.937078000	-0.200234000
H	2.957662000	-2.752198000	-0.554688000
O	1.165942000	-2.149759000	-1.249669000
H	0.328646000	-2.608637000	-1.115256000
H	1.073259000	-1.305208000	-0.777360000
O	1.842439000	0.153369000	0.511753000
H	2.047986000	1.051564000	0.223305000
H	2.692957000	-0.324861000	0.477700000

S-2-RC(SAPE)

-4.85480612 Nimag=0

C	-2.500388000	-1.320349000	3.003514000
H	-1.866641000	-1.448711000	2.120352000
H	-2.892677000	-2.297718000	3.300877000
C	-1.717938000	-0.666281000	4.148166000
H	-2.403420000	-0.596590000	5.011705000
H	-3.353560000	-0.695832000	2.716955000
C	-0.540561000	-1.568415000	4.550572000
H	-0.880094000	-2.599671000	4.691481000
H	0.203148000	-1.566334000	3.746933000
S	0.419601000	-1.117894000	6.036222000
C	-0.690919000	-1.598970000	7.386193000
H	-1.609541000	-1.008227000	7.399420000
H	-0.931144000	-2.664349000	7.327874000
H	-0.140930000	-1.413123000	8.312151000
N	-1.221028000	0.640176000	3.710684000
H	-0.793603000	1.140472000	4.484755000
H	-1.997928000	1.217409000	3.401394000
O	0.029427000	2.928283000	6.383615000
H	-0.443282000	3.124067000	7.220716000
O	-1.184264000	3.572032000	8.995701000
H	-1.976284000	3.153526000	9.352672000
H	-0.448037000	3.213648000	9.525503000
O	1.311790000	2.320497000	9.831869000
H	2.058768000	2.843831000	10.146533000
H	1.453486000	2.237701000	8.871247000
O	1.152692000	2.132982000	6.872978000
H	0.896971000	1.240872000	6.558399000

S-2-H⁺-RC(SAPE)

-4.78757540 Nimag=0

C	0.348769000	-0.256479000	2.362218000
H	0.591758000	-1.248889000	1.967388000
H	-0.603297000	0.051395000	1.924056000
C	0.227595000	-0.265054000	3.881945000
H	0.002450000	0.745838000	4.237322000
H	1.109205000	0.453570000	2.026589000
C	-0.835807000	-1.234927000	4.399162000
H	-1.778097000	-1.004620000	3.893349000
H	-0.568149000	-2.268997000	4.150857000
S	-1.052844000	-1.074946000	6.205094000
C	-1.973788000	-2.590346000	6.579546000
H	-2.936845000	-2.593912000	6.064189000
H	-1.398661000	-3.484361000	6.327161000
H	-2.152590000	-2.571474000	7.657082000
N	1.573688000	-0.591245000	4.499019000
H	2.282827000	0.180730000	4.331766000
H	1.947666000	-1.460101000	4.113434000
H	1.477129000	-0.727426000	5.513159000
O	3.310542000	1.475799000	4.183348000
H	3.792145000	1.744644000	5.001279000
H	3.930002000	1.613997000	3.456412000
O	4.594030000	2.039967000	6.549608000
H	4.375047000	1.426158000	7.265651000
H	4.676157000	2.908897000	6.961848000

O	2.494656000	-0.970205000	7.728122000
O	3.639457000	-0.313358000	8.344165000
H	4.180596000	-1.080996000	8.597761000
H	1.833513000	-0.882061000	8.435887000

S-3-RC(SAPE)

-8.74583854 Nimag=0

C	-2.885286000	3.899976000	1.624051000
H	-2.109778000	4.102364000	0.873030000
H	-3.627705000	3.222274000	1.170720000
C	-2.239359000	3.235622000	2.841544000
H	-1.551478000	3.946221000	3.307987000
C	-3.289533000	2.791108000	3.864069000
H	-3.904452000	3.662404000	4.105243000
H	-3.939492000	2.021352000	3.432139000
S	-2.567819000	2.145095000	5.413624000
C	-4.050878000	2.106846000	6.453005000
H	-4.489654000	3.102316000	6.563970000
H	-4.793172000	1.411363000	6.051890000
H	-3.726428000	1.750623000	7.433589000
O	-3.498688000	5.114180000	2.037431000
C	-4.028448000	5.856349000	0.952262000
H	-3.246141000	6.134245000	0.229177000
H	-4.814176000	5.297176000	0.419727000
H	-4.463883000	6.766738000	1.372095000
S	-1.254067000	1.744102000	2.338961000
S	0.271544000	2.425222000	1.179128000
C	2.231412000	1.615120000	2.977194000
H	1.417755000	1.017380000	3.409457000
H	2.737888000	0.998262000	2.218551000
C	1.657832000	2.874050000	2.324311000
H	1.249230000	3.531088000	3.095376000
C	2.708535000	3.626423000	1.486643000
H	3.650624000	3.640851000	2.042268000
H	2.902102000	3.083129000	0.554653000
S	2.319468000	5.327635000	0.972461000
C	2.788440000	6.281731000	2.441709000
H	2.154316000	6.066422000	3.303469000
H	3.840507000	6.123557000	2.696535000
H	2.650063000	7.330714000	2.166309000
O	3.155759000	1.988441000	4.001448000
C	3.820976000	0.863859000	4.564218000
H	3.111130000	0.166754000	5.034005000
H	4.399105000	0.319033000	3.803419000
H	4.504113000	1.244218000	5.327144000
O	0.029588000	3.390181000	8.065131000
H	0.884862000	3.409846000	7.580260000
O	2.553884000	3.705269000	6.623068000
H	2.681761000	3.253116000	5.776519000
H	2.375685000	4.630593000	6.386421000
O	1.133873000	6.313799000	6.149299000
H	1.240603000	7.082637000	6.722055000
H	0.410057000	5.803123000	6.556610000
O	-0.749275000	4.359567000	7.300759000
H	-1.255794000	3.768203000	6.705196000

S-4-RC(SAPE)

-6.80562673 Nimag=0

S	-3.659937000	4.544922000	1.929586000
C	-2.444916000	2.592281000	3.392672000
H	-2.314380000	3.438271000	4.083454000
H	-3.400016000	2.100582000	3.645903000
C	-2.486704000	3.123160000	1.955219000
H	-1.498722000	3.529304000	1.721002000
C	-2.918870000	2.109865000	0.885169000
H	-3.877516000	1.660684000	1.166031000
H	-3.064057000	2.637357000	-0.060775000
S	-1.715818000	0.758606000	0.550657000
C	-2.433872000	0.123090000	-0.988583000
H	-2.456219000	0.885042000	-1.773372000
H	-1.790319000	-0.699114000	-1.312032000
H	-3.441745000	-0.267926000	-0.822049000
O	-1.366786000	1.680685000	3.522127000
C	-1.319771000	1.058661000	4.792387000
H	-0.456134000	0.388927000	4.786816000
H	-1.197032000	1.794132000	5.603583000
H	-2.228138000	0.467870000	4.991366000
C	-2.827159000	5.721494000	0.788143000
H	-2.623101000	5.228655000	-0.166858000
H	-3.612875000	6.466185000	0.612555000
C	-1.604597000	6.364483000	1.357663000
H	-1.764103000	6.966034000	2.252523000
C	-0.372706000	6.239833000	0.858161000
H	-0.161690000	5.640877000	-0.024502000
H	0.473335000	6.739466000	1.322601000
O	0.438447000	3.058387000	-3.787373000
H	0.526405000	2.878397000	-2.820291000
O	0.802292000	2.622905000	-1.005501000
H	0.198911000	2.046024000	-0.504661000
H	1.661044000	2.169666000	-0.994998000
O	3.354773000	1.368003000	-2.003903000
H	4.206691000	1.819885000	-1.974933000
H	2.962162000	1.638625000	-2.849547000
O	1.519584000	2.232512000	-4.321689000
H	1.007239000	1.518569000	-4.733055000

Se-1-RC(SAPE)

-5.99850631 Nimag=0

C	0.548500000	-0.273218000	4.932784000

H	-0.005179000	-0.280620000	3.987993000
H	0.066507000	-0.980833000	5.615265000
C	2.023062000	-0.650195000	4.712349000
H	2.538418000	-0.607901000	5.675791000
H	0.476562000	0.724229000	5.369542000
C	2.131509000	-2.079406000	4.168244000
H	1.698759000	-2.774186000	4.891505000
H	1.592135000	-2.182663000	3.222742000
Se	4.004290000	-2.656015000	3.872857000
C	3.668308000	-4.590706000	3.831626000
H	3.284681000	-4.929509000	4.795372000
H	2.976629000	-4.842246000	3.026505000
H	4.634750000	-5.059778000	3.637201000
N	2.732350000	0.279513000	3.817751000
H	2.329919000	0.386867000	2.889614000
S	3.355399000	1.734553000	4.331488000
O	3.135145000	2.675286000	3.250170000
O	2.908197000	2.013483000	5.681757000
C	5.120293000	1.434009000	4.422487000
H	5.485632000	1.156470000	3.433536000
H	5.574531000	2.371005000	4.751031000
H	5.314521000	0.644427000	5.148862000
H	4.087222000	-2.254039000	1.308219000
O	4.061418000	-1.908806000	0.390133000
O	3.635880000	-3.097928000	-0.345549000
H	2.729117000	-2.838929000	-0.609171000
O	0.930873000	-2.089043000	-1.127152000
H	0.074461000	-2.475516000	-0.910593000
H	0.954392000	-1.238922000	-0.656855000
O	1.979678000	0.154585000	0.535209000
H	2.218692000	1.034150000	0.216823000
H	2.782168000	-0.388291000	0.413371000

Se-2-RC(SAPE)

-4.82064953 Nimag=0

C	-2.599527000	-1.216153000	3.044793000
H	-2.040961000	-1.400969000	2.121829000
H	-3.056395000	-2.154339000	3.373897000
C	-1.684468000	-0.635165000	4.130997000
H	-2.302694000	-0.497394000	5.036180000
H	-3.410636000	-0.516807000	2.813677000
C	-0.578556000	-1.642702000	4.461832000
H	-0.996622000	-2.631640000	4.665590000
H	0.114072000	-1.726056000	3.619155000
Se	0.609007000	-1.245760000	5.996093000
C	-0.599152000	-1.722222000	7.470613000
H	-1.474922000	-1.073173000	7.488880000
H	-0.895693000	-2.768913000	7.383261000
H	-0.023802000	-1.582011000	8.387916000
N	-1.098239000	0.617761000	3.648618000
H	-0.599892000	1.089055000	4.398320000
H	-1.837307000	1.255053000	3.365405000
O	0.103418000	2.936706000	6.366388000
H	-0.427842000	3.124392000	7.169555000
O	-1.281761000	3.567255000	8.896830000
H	-2.079163000	3.128303000	9.215180000
H	-0.565271000	3.237290000	9.470392000
O	1.201104000	2.404283000	9.889137000
H	1.912880000	2.963556000	10.223074000
H	1.392357000	2.299711000	8.939461000
O	1.193558000	2.143936000	6.928674000
H	0.960152000	1.249033000	6.602733000

Se-2-H$^+$-RC(SAPE)

-4.75364227 Nimag=0

C	0.399819000	-0.374051000	2.201545000
H	0.635810000	-1.412731000	1.946003000
H	-0.485988000	-0.085237000	1.631032000
C	0.119159000	-0.208551000	3.691658000
H	-0.081329000	0.845974000	3.908163000
H	1.224172000	0.265838000	1.874915000
C	-1.048634000	-1.061198000	4.187838000
H	-1.946552000	-0.784307000	3.632418000
H	-0.861722000	-2.126570000	4.026023000
Se	-1.395647000	-0.796285000	6.125500000
C	-1.853213000	-2.645850000	6.606208000
H	-2.745557000	-2.955618000	6.062153000
H	-1.017841000	-3.320088000	6.415957000
H	-2.070786000	-2.619701000	7.675657000
N	1.361328000	-0.526606000	4.494887000
H	2.107152000	0.221670000	4.397149000
H	1.761406000	-1.423220000	4.214052000
H	1.080843000	-0.607076000	5.487123000
O	3.183946000	1.479646000	4.315371000
H	3.713267000	1.690720000	5.122325000
H	3.767359000	1.657836000	3.567741000
O	4.636776000	1.930210000	6.595492000
H	4.480355000	1.300793000	7.316496000
H	4.740109000	2.792653000	7.016299000
O	2.732774000	-0.944725000	8.059283000
O	3.950054000	-0.306977000	8.543734000
H	4.522687000	-1.080062000	8.686897000
H	2.177407000	-0.884888000	8.855499000

Se-3-RC(SAPE)

-8.67663717 Nimag=0

C	-0.277768000	4.141351000	0.743744000
H	0.761053000	4.243074000	0.401924000
H	-0.791686000	3.441781000	0.063645000

C	-0.290306000	3.592907000	2.173610000
H	0.168519000	4.329041000	2.837528000
C	-1.712995000	3.249586000	2.635256000
H	-2.410917000	3.971482000	2.207883000
H	-2.015266000	2.258171000	2.283451000
Se	-2.087195000	3.231929000	4.574275000
C	-2.465455000	5.145880000	4.811702000
H	-1.593704000	5.750730000	4.560163000
H	-3.323699000	5.434432000	4.203183000
H	-2.706853000	5.272879000	5.868934000
O	-0.921398000	5.409530000	0.740428000
C	-0.896949000	6.031647000	-0.532549000
H	0.132940000	6.207922000	-0.879779000
H	-1.423361000	5.430954000	-1.291298000
H	-1.405198000	6.993612000	-0.426822000
S	0.700031000	2.028925000	2.276540000
S	2.623094000	2.522295000	1.835151000
C	3.942550000	1.526464000	4.035761000
H	3.075251000	0.859935000	4.145699000
H	4.654668000	1.040480000	3.351374000
C	3.487693000	2.863646000	3.440209000
H	2.765143000	3.343559000	4.103784000
C	4.658878000	3.807625000	3.146499000
H	5.342172000	3.820618000	3.997570000
H	5.229092000	3.462335000	2.278620000
Se	4.189369000	5.688882000	2.763308000
C	4.436546000	6.436081000	4.564736000
H	3.721893000	6.016593000	5.272218000
H	5.462074000	6.278179000	4.902985000
H	4.252374000	7.507853000	4.461566000
O	4.560391000	1.737569000	5.306741000
C	5.147842000	0.548893000	5.825025000
H	4.396273000	-0.238896000	5.982017000
H	5.927817000	0.164312000	5.151946000
H	5.598913000	0.806271000	6.785873000
H	-0.357131000	3.220411000	6.694805000
O	-0.062555000	3.506599000	7.579784000
O	0.432424000	2.251841000	8.137495000
H	1.404693000	2.377550000	8.040865000
O	3.248808000	2.825806000	7.892559000
H	3.632149000	2.606796000	7.029861000
H	3.159221000	3.792907000	7.891108000
H	1.245992000	5.032987000	7.767109000
O	2.045857000	5.588181000	7.795889000
H	1.944448000	6.118918000	8.595144000

Se-4-RC(SAPE)

-6.77307510 Nimag=0

S	2.983515000	2.464142000	3.403925000
C	2.113983000	-0.031043000	2.744887000
H	3.193756000	-0.239064000	2.729009000
H	1.754010000	-0.212723000	3.772077000
C	1.877146000	1.429880000	2.349829000
H	2.220156000	1.558750000	1.319633000
C	0.438747000	1.926422000	2.505189000
H	0.078621000	1.750536000	3.521989000
H	0.396479000	2.997801000	2.305847000
Se	-0.888514000	1.119230000	1.249582000
C	-2.225354000	2.554219000	1.403205000
H	-1.817405000	3.508592000	1.065283000
H	-3.056282000	2.265557000	0.755823000
H	-2.581607000	2.631992000	2.431986000
O	1.435896000	-0.873405000	1.826221000
C	1.506277000	-2.244007000	2.174995000
H	0.963343000	-2.796987000	1.404462000
H	2.547104000	-2.602939000	2.206743000
H	1.038199000	-2.440334000	3.152495000
C	3.534962000	3.767667000	2.230542000
H	2.663235000	4.256277000	1.785807000
H	4.013303000	4.493209000	2.899384000
C	4.496890000	3.287614000	1.192933000
H	5.440078000	2.895272000	1.573124000
C	4.270763000	3.300087000	-0.122889000
H	3.340890000	3.668919000	-0.549224000
H	5.018403000	2.936789000	-0.823046000
O	-1.120631000	4.628796000	-3.003285000
H	-0.615892000	3.914228000	-2.545488000
O	0.435731000	2.576700000	-1.794104000
H	0.092632000	2.078210000	-1.031063000
H	0.474179000	1.934421000	-2.521902000
O	-0.008008000	1.311160000	-4.493355000
H	0.652685000	1.340201000	-5.195805000
H	-0.523963000	2.125528000	-4.608724000
O	-1.618673000	3.909742000	-4.174014000
H	-2.553957000	3.811164000	-3.935270000

Te-1-RC(SAPE)

-5.96640337 Nimag=0

C	-3.452095000	-0.700205000	2.605441000
H	-2.912984000	-1.215280000	1.803478000
H	-3.932287000	-1.457742000	3.233314000
C	-2.497212000	0.167376000	3.443231000
H	-3.080667000	0.679527000	4.213400000
H	-4.233005000	-0.082165000	2.159403000
C	-1.443993000	-0.707649000	4.129478000
H	-1.929694000	-1.397117000	4.823284000
H	-0.878340000	-1.291445000	3.399464000
Te	-0.000616000	0.429340000	5.309871000

C	0.575384000	-1.246011000	6.554701000
H	-0.279730000	-1.584682000	7.140052000
H	0.970691000	-2.050607000	5.934641000
H	1.358114000	-0.880945000	7.222026000
N	-1.823633000	1.220073000	2.659317000
H	-1.266787000	0.881570000	1.876452000
S	-2.576775000	2.641527000	2.219932000
O	-2.134661000	2.935975000	0.870260000
O	-3.984492000	2.582776000	2.559541000
C	-1.834988000	3.865529000	3.299102000
H	-0.760534000	3.897474000	3.117410000
H	-2.296324000	4.820575000	3.039604000
H	-2.051896000	3.610792000	4.336790000
O	3.450685000	-0.678202000	2.491942000
H	3.070384000	-1.213173000	1.765093000
O	2.216462000	-2.109311000	0.177942000
H	1.814350000	-2.985666000	0.185173000
H	1.485440000	-1.494107000	0.000873000
O	0.458442000	0.289833000	0.432879000
H	0.393841000	1.024006000	-0.191053000
H	1.194039000	0.524170000	1.030451000
O	2.554372000	0.475945000	2.484969000
H	2.063172000	0.341050000	3.322033000

Te-2-RC(SAPE)

-4.78828584 Nimag=0

C	-1.377357000	-3.145947000	8.502482000
H	-0.374951000	-3.585040000	8.447142000
H	-1.575768000	-2.885442000	9.548303000
C	-1.496934000	-1.915762000	7.583777000
H	-2.517500000	-1.522489000	7.678211000
H	-2.100125000	-3.914819000	8.208532000
C	-0.535965000	-0.810246000	8.017560000
H	-0.810529000	-0.406683000	8.994235000
H	0.495277000	-1.170898000	8.066042000
Te	-0.587365000	0.844401000	6.594860000
C	0.288015000	2.274754000	7.975159000
H	-0.376491000	2.429289000	8.826089000
H	1.266416000	1.925831000	8.307088000
H	0.402439000	3.211665000	7.426322000
N	-1.305471000	-2.189750000	6.153615000
H	-1.993665000	-2.860710000	5.824078000
H	-0.390499000	-2.602107000	5.983710000
O	4.742249000	-1.364246000	6.795932000
H	3.917655000	-1.230349000	6.270660000
O	2.417406000	-1.080218000	5.168712000
H	1.728161000	-0.425166000	5.374450000
H	2.819709000	-0.779598000	4.337129000
O	4.508327000	-0.273184000	3.160801000
H	4.839778000	-0.887483000	2.494734000
H	5.077420000	-0.419705000	3.933702000
O	5.719422000	-0.671912000	5.958651000
H	5.860947000	0.132914000	6.481926000

Te-2-H$^+$-RC(SAPE)

-4.72205627 Nimag=0

C	0.426400000	-0.339100000	2.188800000
H	0.644800000	-1.388000000	1.961200000
H	-0.429000000	-0.039200000	1.579100000
C	0.091000000	-0.139800000	3.665000000
H	-0.084300000	0.924200000	3.853700000
H	1.279400000	0.273500000	1.883300000
C	-1.124000000	-0.947500000	4.120600000
H	-1.993000000	-0.624900000	3.545600000
H	-0.986100000	-2.018800000	3.948000000
Te	-1.626900000	-0.725000000	6.244800000
C	-1.738700000	-2.841100000	6.686400000
H	-2.522800000	-3.290900000	6.078300000
H	-0.776500000	-3.324300000	6.521600000
H	-2.010700000	-2.907500000	7.741400000
N	1.295800000	-0.474400000	4.515200000
H	2.076900000	0.233600000	4.402000000
H	1.661800000	-1.400200000	4.287200000
H	0.991400000	-0.495300000	5.505000000
O	3.221900000	1.436200000	4.293700000
H	3.776900000	1.624700000	5.088700000
H	3.800900000	1.576000000	3.534600000
O	4.745700000	1.825800000	6.541400000
H	4.538100000	1.239400000	7.285400000
H	4.928600000	2.689800000	6.931000000
O	2.620400000	-0.830200000	8.071600000
O	3.866500000	-0.264300000	8.573200000
H	4.371800000	-1.071100000	8.772300000
H	2.041000000	-0.680100000	8.838200000

Te-3-RC(SAPE)

-8.61456805 Nimag=0

C	-0.119370000	4.439169000	0.741420000
H	0.915570000	4.567334000	0.394250000
H	-0.657324000	3.838483000	-0.010624000
C	-0.111585000	3.711279000	2.089298000
H	0.397547000	4.336294000	2.827379000
C	-1.520021000	3.375399000	2.570789000
H	-2.091443000	4.300490000	2.661527000
H	-2.032788000	2.714613000	1.867611000
Te	-1.614851000	2.430315000	4.541144000
C	-3.550617000	3.267644000	5.035681000
H	-3.501109000	4.356406000	5.013498000
H	-4.303564000	2.894289000	4.341525000

H	-3.781559000	2.930128000	6.047655000
O	-0.740639000	5.705644000	0.912186000
C	-0.703610000	6.496341000	-0.263012000
H	0.329705000	6.699489000	-0.584781000
H	-1.240983000	6.015871000	-1.096135000
H	-1.193579000	7.443873000	-0.024597000
S	0.829693000	2.118254000	1.962884000
S	2.786686000	2.612412000	1.693450000
C	3.742152000	1.425270000	4.004810000
H	2.794218000	0.868851000	3.993910000
H	4.475161000	0.855456000	3.413158000
C	3.532857000	2.807831000	3.378924000
H	2.828117000	3.385273000	3.981925000
C	4.839449000	3.582974000	3.205548000
H	5.384606000	3.592547000	4.151223000
H	5.481951000	3.111604000	2.457221000
Te	4.641164000	5.684893000	2.641821000
C	5.398009000	6.487768000	4.506189000
H	4.712663000	6.257125000	5.320256000
H	6.397810000	6.097890000	4.698221000
H	5.450678000	7.569505000	4.366049000
O	4.205166000	1.570004000	5.347726000
C	4.511430000	0.318114000	5.949935000
H	3.624871000	-0.331123000	6.006784000
H	5.300374000	-0.210232000	5.394631000
H	4.865377000	0.526436000	6.962082000
H	-0.354217000	4.029550000	6.513903000
O	-0.013395000	4.497773000	7.304407000
O	0.213597000	3.381182000	8.216725000
H	1.177249000	3.228573000	8.089344000
O	3.093330000	3.187612000	7.846721000
H	3.404072000	2.795345000	7.017970000
H	3.196496000	4.146035000	7.722782000
H	1.605553000	5.743954000	7.327941000
O	2.511782000	6.096993000	7.374738000
H	2.489054000	6.719589000	8.111428000

Te-4-RC(SAPE)

-6.74241078 Nimag=0

S	2.242281000	0.725171000	2.357318000
C	1.076091000	-1.543002000	1.411021000
H	2.098354000	-1.798722000	1.097958000
H	0.944109000	-1.898113000	2.447194000
C	0.891815000	-0.024536000	1.343033000
H	1.067523000	0.292260000	0.311539000
C	-0.456620000	0.485181000	1.844019000
H	-0.644076000	0.153212000	2.867225000
H	-0.477258000	1.575325000	1.825282000
Te	-2.216753000	-0.086556000	0.650707000
C	-3.360625000	1.659988000	1.246534000
H	-2.843839000	2.571287000	0.943430000
H	-4.321238000	1.595250000	0.731160000
H	-3.525200000	1.648245000	2.324465000
O	0.128389000	-2.163313000	0.553415000
C	0.158152000	-3.578426000	0.621714000
H	-0.596867000	-3.947773000	-0.076578000
H	1.141745000	-3.976667000	0.328676000
H	-0.081296000	-3.941954000	1.633144000
C	2.781161000	2.145310000	1.321856000
H	1.923679000	2.786088000	1.096895000
H	3.431276000	2.696855000	2.011992000
C	3.528696000	1.756512000	0.087902000
H	4.442751000	1.186159000	0.253218000
C	3.152142000	2.050653000	-1.158655000
H	2.246548000	2.612129000	-1.375058000
H	3.748128000	1.740457000	-2.012767000
O	-0.626528000	4.540298000	-1.997075000
H	-0.770617000	3.571394000	-2.130625000
O	-0.965961000	1.778094000	-2.478737000
H	-1.333655000	1.214237000	-1.775356000
H	-1.628971000	1.772731000	-3.188127000
O	-2.865080000	2.825873000	-4.581493000
H	-2.624473000	2.857406000	-5.515316000
H	-2.533653000	3.661554000	-4.215195000
O	-1.668580000	5.095671000	-2.858959000
H	-2.282048000	5.439122000	-2.190254000

S-1-PC(SAPE)

-6.11574948 Nimag=0

C	-3.226095000	-0.611412000	4.735281000
H	-2.987308000	-1.277586000	3.900836000
H	-4.016574000	-1.079409000	5.330514000
C	-1.981725000	-0.335807000	5.599076000
H	-2.260806000	0.360396000	6.393119000
H	-3.623401000	0.319578000	4.323327000
C	-1.496011000	-1.648437000	6.236438000
H	-2.274196000	-2.099992000	6.859452000
H	-1.225760000	-2.373863000	5.462528000
S	0.034143000	-1.602265000	7.269429000
C	-0.609570000	-0.689178000	8.701128000
H	-0.804062000	0.344031000	8.406906000
H	-1.504105000	-1.181168000	9.090846000
H	0.187296000	-0.713051000	9.447419000
N	-0.900866000	0.304920000	4.852058000
H	-0.469869000	-0.205756000	4.077022000
S	-0.460741000	1.875939000	4.973254000
O	-0.771790000	2.318877000	6.320323000
O	0.879077000	1.968348000	4.433671000

C	-1.516087000	2.824300000	3.867728000
H	-2.552698000	2.749198000	4.197810000
H	-1.179133000	3.861332000	3.926213000
H	-1.396580000	2.444402000	2.852089000
O	0.214848000	-3.031374000	7.730117000
H	1.085917000	-4.254866000	6.614966000
O	0.368327000	-1.291375000	2.596264000
H	1.177234000	-0.820086000	2.361812000
H	0.671818000	-2.194872000	2.814502000
O	1.217252000	-3.965922000	3.271754000
H	0.848357000	-4.715314000	2.790720000
H	1.327236000	-4.286913000	4.191760000
O	1.485366000	-4.867130000	5.961302000
H	2.378105000	-5.026244000	6.288733000

S-2-PC(SAPE)

-4.93500617 Nimag=0

C	-2.440882000	-1.763536000	3.013751000
H	-1.847081000	-1.809893000	2.095753000
H	-2.491835000	-2.763990000	3.454423000
C	-1.848001000	-0.751617000	4.001613000
H	-2.462960000	-0.795153000	4.916325000
H	-3.460380000	-1.470444000	2.739621000
C	-0.410083000	-1.151014000	4.365906000
H	-0.314507000	-2.228226000	4.537587000
H	0.264403000	-0.873692000	3.549264000
S	0.359202000	-0.388079000	5.857210000
C	-0.608406000	-1.204695000	7.160594000
H	-1.666198000	-0.949615000	7.073630000
H	-0.454174000	-2.284538000	7.086379000
H	-0.204639000	-0.844425000	8.108765000
N	-1.837524000	0.581379000	3.389673000
H	-1.487581000	1.259574000	4.061072000
H	-2.787878000	0.856604000	3.156976000
O	-0.022732000	1.075247000	5.880556000
H	0.822780000	2.490950000	6.826005000
O	0.810696000	-0.270707000	10.382086000
H	1.489919000	-0.684951000	10.926684000
H	1.085866000	0.664530000	10.331636000
O	1.595700000	2.546327000	10.106963000
H	1.036311000	3.160385000	10.596976000
H	1.476218000	2.804023000	9.170090000
O	1.204505000	3.227399000	7.343825000
H	1.972059000	3.511858000	6.834539000

S-2-H^{+}-PC(SAPE)

-4.87044719 Nimag=0

C	-0.551334000	-2.067120000	2.647492000
H	-1.033029000	-1.351893000	1.974663000
H	0.510234000	-2.111622000	2.394351000
C	-0.718853000	-1.667848000	4.114105000
H	-0.229335000	-2.414310000	4.748979000
H	-0.969712000	-3.061748000	2.460678000
C	-0.114918000	-0.285650000	4.390706000
H	0.902314000	-0.284618000	3.988260000
H	-0.680243000	0.514469000	3.903812000
S	0.079774000	0.141801000	6.179462000
C	0.963142000	1.707027000	5.937143000
H	1.946428000	1.496428000	5.509011000
H	0.385557000	2.383539000	5.303489000
H	1.084444000	2.131530000	6.936158000
N	-2.189334000	-1.731444000	4.478505000
H	-2.358673000	-1.778778000	5.505250000
H	-2.589960000	-2.585524000	4.088170000
H	-2.745161000	-0.923648000	4.086655000
O	-1.292353000	0.478942000	6.714378000
H	-2.441863000	-1.147096000	7.633129000
O	-2.871730000	-1.911029000	7.219019000
H	-3.000787000	-2.568941000	7.912903000
H	-2.658440000	1.939892000	6.073679000
O	-3.193047000	2.388998000	5.398265000
H	-3.805595000	2.959965000	5.877839000
H	-3.620356000	1.183454000	4.111620000
O	-3.592788000	0.427954000	3.480898000
H	-4.444183000	0.421970000	3.027280000

S-3-PC(SAPE)

-8.82755779 Nimag=0

C	-3.038374000	3.727757000	1.462438000
H	-2.464167000	3.687493000	0.525597000
H	-3.795845000	2.926694000	1.427160000
C	-2.079522000	3.499963000	2.634812000
H	-1.345486000	4.308593000	2.653477000
C	-2.802483000	3.462779000	3.979608000
H	-3.429713000	4.354068000	4.067068000
H	-3.429471000	2.570165000	4.090073000
S	-1.636312000	3.453553000	5.411769000
C	-2.871084000	3.811831000	6.694841000
H	-3.338396000	4.779545000	6.496751000
H	-3.612258000	3.007569000	6.704999000
H	-2.330104000	3.823753000	7.642831000
O	-3.656963000	4.997413000	1.609874000
C	-4.486720000	5.334508000	0.511308000
H	-3.917333000	5.376676000	-0.430055000
H	-5.313521000	4.616941000	0.388304000
H	-4.902937000	6.323477000	0.719092000
S	-1.142882000	1.916641000	2.392930000
S	0.354120000	2.370835000	1.091191000
C	2.402608000	1.923849000	2.920106000

H	1.636970000	1.316815000	3.422485000
H	2.964004000	1.263029000	2.240607000
C	1.731810000	3.047669000	2.126940000
H	1.285839000	3.759705000	2.823108000
C	2.711267000	3.773784000	1.186411000
H	3.671871000	3.879189000	1.699100000
H	2.897724000	3.170640000	0.290581000
S	2.208971000	5.408539000	0.566575000
C	2.640178000	6.481071000	1.964258000
H	2.055845000	6.256212000	2.859078000
H	3.708702000	6.426401000	2.191266000
H	2.401441000	7.497480000	1.640421000
O	3.280871000	2.496903000	3.888962000
C	4.007782000	1.513325000	4.613072000
H	3.337632000	0.842134000	5.170978000
H	4.638373000	0.906945000	3.945786000
H	4.647632000	2.044901000	5.321626000
O	-0.774843000	4.683771000	5.266615000
H	1.074538000	4.656206000	5.820573000
O	-0.725857000	3.310176000	9.628289000
H	-0.669227000	2.606706000	10.285067000
H	0.204961000	3.495840000	9.400396000
O	2.063893000	3.897273000	8.858223000
H	2.417825000	4.673582000	9.307955000
H	2.080737000	4.140773000	7.908071000
O	2.011225000	4.611615000	6.094680000
H	2.416980000	4.001913000	5.463707000

S-4-PC(SAPE)

-6.89021963 Nimag=0

S	-4.027894000	4.582110000	1.713318000
C	-2.442668000	2.972109000	3.204242000
H	-2.258521000	3.841676000	3.851894000
H	-3.386906000	2.506000000	3.537125000
C	-2.577381000	3.450995000	1.754147000
H	-1.706932000	4.063913000	1.505126000
C	-2.731359000	2.317215000	0.729480000
H	-3.242860000	1.452684000	1.162749000
H	-3.288538000	2.656355000	-0.146834000
S	-1.119913000	1.720176000	0.042959000
C	-1.816902000	0.407933000	-1.005203000
H	-2.554759000	0.830662000	-1.691954000
H	-0.979429000	-0.027777000	-1.553554000
H	-2.265084000	-0.353634000	-0.361108000
O	-1.378172000	2.040248000	3.298543000
C	-1.183761000	1.559658000	4.617141000
H	-0.348063000	0.856478000	4.578997000
H	-0.934547000	2.374427000	5.314779000
H	-2.075824000	1.036186000	4.996587000
C	-3.485669000	5.857237000	0.505177000
H	-3.185962000	5.372381000	-0.428126000
H	-4.415546000	6.405570000	0.312825000
C	-2.425406000	6.773507000	1.023974000
H	-2.705519000	7.377469000	1.886994000
C	-1.195460000	6.880688000	0.515426000
H	-0.870885000	6.287339000	-0.336065000
H	-0.470512000	7.570695000	0.938819000
O	-0.658496000	2.823478000	-0.883833000
H	1.148051000	3.082939000	-1.317569000
O	1.123757000	-1.359461000	-2.320278000
H	1.247048000	-1.835537000	-3.149566000
H	1.855049000	-0.713385000	-2.308660000
O	3.261423000	0.662307000	-2.234742000
H	3.939654000	0.507862000	-1.566878000
H	2.884161000	1.536005000	-2.002070000
O	2.089032000	3.198118000	-1.567334000
H	2.067494000	3.807777000	-2.314027000

Se-1-PC(SAPE)

-6.06670076 Nimag=0

C	-3.765766000	-0.367520000	4.825265000
H	-3.538800000	-0.941218000	3.921802000
H	-4.537013000	-0.904787000	5.386032000
C	-2.505057000	-0.168410000	5.686938000
H	-2.771918000	0.439575000	6.554835000
H	-4.182010000	0.596237000	4.520602000
C	-1.983186000	-1.525347000	6.173824000
H	-2.718416000	-2.058469000	6.780904000
H	-1.696121000	-2.167791000	5.336932000
Se	-0.300734000	-1.507221000	7.268368000
C	-1.113504000	-0.881991000	8.956786000
H	-1.422884000	0.157493000	8.836621000
H	-1.947463000	-1.539975000	9.203027000
H	-0.326450000	-0.961400000	9.708061000
N	-1.447146000	0.560224000	4.985707000
H	-1.059748000	0.155396000	4.128728000
S	-1.007114000	2.108527000	5.279857000
O	-1.251799000	2.374647000	6.685855000
O	0.305123000	2.277768000	4.693059000
C	-2.117225000	3.179803000	4.355469000
H	-3.136739000	3.053100000	4.721223000
H	-1.781621000	4.204011000	4.530920000
H	-2.044454000	2.934382000	3.295003000
O	-0.071618000	-3.142082000	7.550902000
H	0.630181000	-4.126430000	6.214529000
O	-0.292876000	-0.766246000	2.511764000
H	0.494133000	-0.268334000	2.258884000
H	0.030891000	-1.681638000	2.634055000

O	0.615736000	-3.464621000	2.904194000
H	0.231433000	-4.162630000	2.361868000
H	0.755672000	-3.878499000	3.784150000
O	0.972002000	-4.644507000	5.447030000
H	1.869294000	-4.891347000	5.698204000

Se-2-PC(SAPE)

-4.88536951 Nimag=0

C	-1.689395000	-1.550486000	2.150744000
H	-0.836752000	-1.584519000	1.465649000
H	-1.866633000	-2.555258000	2.546391000
C	-1.445504000	-0.542274000	3.281599000
H	-2.321148000	-0.589269000	3.952253000
H	-2.574365000	-1.261309000	1.572775000
C	-0.204838000	-0.945230000	4.083466000
H	-0.200735000	-2.009760000	4.336512000
H	0.703760000	-0.707913000	3.522507000
Se	0.077163000	-0.037875000	5.853676000
C	-1.294689000	-1.037869000	6.864543000
H	-2.268970000	-0.844146000	6.416575000
H	-1.040333000	-2.099830000	6.844857000
H	-1.238341000	-0.658372000	7.884348000
N	-1.238748000	0.793960000	2.714724000
H	-1.164668000	1.467091000	3.474357000
H	-2.050718000	1.057917000	2.163235000
O	-0.606700000	1.484214000	5.679108000
H	-0.201830000	2.859013000	6.808403000
O	1.066940000	-0.308977000	9.589247000
H	1.879096000	-0.650499000	9.980677000
H	1.136306000	0.657938000	9.708627000
O	1.181414000	2.609905000	9.823841000
H	0.644633000	2.969562000	10.539686000
H	0.784007000	2.985889000	9.009440000
O	-0.007203000	3.596379000	7.430616000
H	0.510450000	4.222115000	6.911146000

Se-2-H$^+$-PC(SAPE)

-4.81325240 Nimag=0

C	-0.647373000	-1.998919000	2.472310000
H	-1.158244000	-1.168837000	1.976257000
H	0.381935000	-2.043718000	2.102881000
C	-0.690228000	-1.850504000	4.004241000
H	-0.128258000	-2.691041000	4.432856000
H	-1.142939000	-2.929007000	2.178986000
C	0.009359000	-0.546207000	4.402873000
H	0.984352000	-0.455564000	3.915294000
H	-0.588921000	0.345773000	4.195720000
Se	0.448934000	-0.451729000	6.339637000
C	1.732441000	1.004437000	6.276619000
H	2.633031000	0.626628000	5.788410000
H	1.288682000	1.835905000	5.728704000
H	1.937876000	1.272938000	7.313617000
N	-2.048891000	-1.903154000	4.577966000
H	-2.590596000	-1.650007000	6.368282000
H	-2.473199000	-2.789565000	4.312258000
H	-2.623511000	-1.175931000	4.142846000
O	-0.846417000	0.508906000	7.030110000
H	-1.604922000	-0.145843000	7.247575000
O	-2.761622000	-1.206709000	7.239107000
H	-2.878648000	-1.892804000	7.907758000
H	-5.585443000	2.184082000	5.782407000
O	-4.791748000	2.469234000	5.311700000
H	-4.917896000	3.417489000	5.178558000
H	-3.877012000	1.309805000	3.990161000
O	-3.375219000	0.725913000	3.393295000
H	-3.817422000	0.824828000	2.541072000

Se-3-PC(SAPE)

-8.74363147 Nimag=0

C	-0.225654000	4.140592000	0.461601000
H	0.734070000	3.988316000	-0.053109000
H	-0.958077000	3.442298000	0.022840000
C	-0.035892000	3.842700000	1.952604000
H	0.689506000	4.546539000	2.367590000
C	-1.333337000	3.952014000	2.741552000
H	-1.787955000	4.928243000	2.564738000
H	-2.053335000	3.163957000	2.493240000
Se	-1.032199000	3.809538000	4.724883000
C	-2.707340000	4.739147000	5.209371000
H	-2.638570000	5.762186000	4.838018000
H	-3.549757000	4.202002000	4.766635000
H	-2.761884000	4.705250000	6.297268000
O	-0.660353000	5.483976000	0.310421000
C	-0.756877000	5.879281000	-1.047050000
H	0.214301000	5.809029000	-1.561005000
H	-1.489677000	5.269518000	-1.599333000
H	-1.086538000	6.921321000	-1.052190000
S	0.665258000	2.139467000	2.178648000
S	2.654310000	2.308431000	1.780569000
C	3.532798000	1.682163000	4.340623000
H	2.550741000	1.195523000	4.422855000
H	4.249652000	0.932752000	3.969177000
C	3.445591000	2.857709000	3.362028000
H	2.803122000	3.631108000	3.785991000
C	4.822899000	3.440166000	3.019664000
H	5.427560000	3.487686000	3.927600000
H	5.356603000	2.802425000	2.307729000
Se	4.852375000	5.243569000	2.217526000
C	4.748862000	6.305414000	3.867713000

H	3.805869000	6.137614000	4.387926000
H	5.596410000	6.080965000	4.517440000
H	4.803596000	7.347913000	3.546690000
O	3.943690000	2.167920000	5.617880000
C	4.129156000	1.123939000	6.564152000
H	3.197957000	0.564561000	6.739606000
H	4.908128000	0.418868000	6.237341000
H	4.442613000	1.592335000	7.500262000
O	0.156574000	4.950213000	5.012138000
H	1.393831000	4.639003000	6.365842000
O	-2.089275000	3.136875000	8.472568000
H	-2.439379000	2.412967000	9.004232000
H	-1.177716000	3.253957000	8.803970000
O	0.683856000	3.521201000	9.352898000
H	0.795412000	4.211052000	10.017452000
H	1.189657000	3.849130000	8.575813000
O	2.056973000	4.452518000	7.066040000
H	2.657044000	3.811751000	6.661654000

Se-4-PC(SAPE)

-6.84159898 Nimag=0

S	2.745784000	2.785491000	3.675218000
C	2.333815000	0.309083000	2.655345000
H	3.423931000	0.170082000	2.691649000
H	1.933403000	0.058646000	3.653269000
C	2.027333000	1.771310000	2.315671000
H	2.579518000	2.044837000	1.412127000
C	0.541108000	2.079905000	2.140755000
H	-0.083881000	1.518737000	2.841273000
H	0.339224000	3.146361000	2.247475000
Se	-0.149949000	1.701144000	0.288105000
C	-2.004247000	2.249736000	0.702194000
H	-1.984749000	3.285463000	1.043576000
H	-2.560667000	2.141447000	-0.229091000
H	-2.407524000	1.578953000	1.464701000
O	1.753015000	-0.532734000	1.671322000
C	2.014099000	-1.907668000	1.896403000
H	1.527552000	-2.460085000	1.088724000
H	3.093465000	-2.123907000	1.878913000
H	1.604803000	-2.247556000	2.860831000
C	3.309406000	4.283264000	2.769661000
H	2.474338000	4.700803000	2.200072000
H	3.535864000	4.978474000	3.586677000
C	4.511927000	4.058142000	1.911698000
H	5.419283000	3.764620000	2.439505000
C	4.531987000	4.179987000	0.582113000
H	3.646326000	4.456555000	0.014928000
H	5.442916000	4.004505000	0.016070000
O	0.467321000	3.005981000	-0.564187000
H	0.705013000	2.882397000	-2.347180000
O	-3.288400000	0.714541000	-2.402976000
H	-4.142822000	0.806517000	-2.840010000
H	-2.641619000	0.888582000	-3.113511000
O	-1.281906000	1.279232000	-4.469513000
H	-0.846421000	0.508669000	-4.852012000
H	-0.548041000	1.814297000	-4.096752000
O	0.818370000	2.823093000	-3.325738000
H	0.822126000	3.739696000	-3.624507000

Te-1-PC(SAPE)

-6.04249726 Nimag=0

C	3.763394000	-9.302242000	4.245588000
H	4.663115000	-9.243151000	4.865149000
H	3.084817000	-10.034126000	4.695530000
C	3.075741000	-7.930795000	4.134173000
H	2.198751000	-8.041106000	3.490142000
H	4.049628000	-9.668428000	3.256794000
C	2.623531000	-7.429873000	5.508513000
H	1.937542000	-8.135015000	5.984232000
H	3.463200000	-7.239211000	6.184024000
Te	1.622862000	-5.482621000	5.420075000
C	-0.005759000	-6.099537000	6.709302000
H	-0.583390000	-6.889656000	6.223776000
H	0.432309000	-6.444018000	7.647293000
H	-0.631489000	-5.222541000	6.882282000
N	3.913948000	-6.887683000	3.508568000
H	4.857455000	-6.839450000	3.915763000
S	4.028056000	-6.834738000	1.835227000
O	5.222619000	-7.509749000	1.361540000
O	2.729097000	-7.206718000	1.305429000
C	4.278531000	-5.081538000	1.566887000
H	5.169653000	-4.760630000	2.108178000
H	4.427465000	-4.961014000	0.491935000
H	3.396466000	-4.535617000	1.899757000
O	2.595858000	-4.494160000	6.628849000
H	4.033103000	-3.474879000	6.486327000
O	4.850987000	-2.921988000	6.464852000
H	4.551951000	-2.037563000	6.228370000
H	6.428092000	-3.629062000	5.901881000
O	7.267855000	-4.047732000	5.608624000
H	7.848453000	-3.980267000	6.375691000
H	6.934604000	-5.803136000	5.061417000
O	6.679731000	-6.699933000	4.755496000
H	7.366003000	-6.950098000	4.125489000

Te-2-PC(SAPE)

-4.86331564 Nimag=0

C	-1.851683000	-1.443037000	2.066556000
H	-1.043689000	-1.553366000	1.336691000

H	-2.141763000	-2.435287000	2.425286000
C	-1.421292000	-0.536508000	3.227349000
H	-2.268821000	-0.491375000	3.934132000
H	-2.717114000	-1.011774000	1.550393000
C	-0.220827000	-1.149491000	3.957511000
H	-0.393203000	-2.199005000	4.217557000
H	0.677307000	-1.082542000	3.336112000
Te	0.347554000	-0.222232000	5.865626000
C	-1.219389000	-1.190659000	6.997307000
H	-2.175233000	-0.851394000	6.597002000
H	-1.111590000	-2.274084000	6.909651000
H	-1.088599000	-0.880028000	8.033564000
N	-1.048668000	0.784336000	2.710549000
H	-0.893163000	1.414796000	3.495594000
H	-1.822632000	1.168870000	2.175465000
O	-0.351055000	1.478107000	5.687872000
H	-0.170602000	2.835899000	6.823895000
O	1.208620000	-0.135789000	9.547118000
H	2.023220000	-0.412432000	9.982293000
H	1.158343000	0.824465000	9.726998000
O	0.985577000	2.733625000	9.916942000
H	0.353846000	3.017494000	10.587870000
H	0.616079000	3.075315000	9.072438000
O	-0.095509000	3.591845000	7.455687000
H	0.396484000	4.264646000	6.971543000

Te-2-H$^+$-PC(SAPE)

-4.80560737 Nimag=0

C	-1.190000000	-0.431287000	2.095271000
H	-0.386179000	-1.081200000	1.739447000
H	-2.145903000	-0.897938000	1.844627000
C	-1.103783000	-0.201303000	3.602670000
H	-1.929837000	0.450853000	3.908704000
H	-1.138012000	0.519099000	1.553644000
C	-1.157420000	-1.504648000	4.406118000
H	-2.073027000	-2.051082000	4.167398000
H	-0.299001000	-2.148407000	4.200084000
Te	-1.195552000	-1.153738000	6.580935000
C	0.121697000	-2.788011000	7.059052000
H	-0.375892000	-3.720962000	6.783223000
H	1.055606000	-2.650454000	6.513796000
H	0.291530000	-2.754632000	8.136656000
N	0.153421000	0.550673000	3.969799000
H	0.131137000	1.496466000	3.590230000
H	1.022564000	0.079849000	3.619038000
H	1.826846000	0.428347000	7.481954000
O	3.711760000	-1.479227000	5.600118000
H	3.564098000	-0.861731000	6.343872000
H	4.603394000	-1.827376000	5.719041000
O	2.489150000	-0.785379000	3.226546000
H	3.107799000	-0.563264000	2.520508000
H	3.043500000	-1.019108000	4.008066000
O	-0.033146000	0.304285000	6.578785000
O	2.757349000	0.224249000	7.672540000
H	3.101353000	0.982947000	8.158674000
H	0.195006000	0.591967000	5.051928000

Te-3-PC(SAPE)

-8.68907469 Nimag=0

C	-0.226349000	4.093522000	0.416719000
H	0.756152000	3.977144000	-0.063361000
H	-0.923097000	3.385620000	-0.062814000
C	-0.089079000	3.773533000	1.908761000
H	0.604568000	4.484167000	2.364720000
C	-1.416824000	3.821017000	2.653668000
H	-1.891657000	4.791171000	2.494091000
H	-2.108091000	3.031897000	2.336419000
Te	-1.169943000	3.605419000	4.830502000
C	-2.856299000	4.891862000	5.250727000
H	-2.597746000	5.884756000	4.879694000
H	-3.748133000	4.500623000	4.755308000
H	-2.986003000	4.893295000	6.332926000
O	-0.690155000	5.428162000	0.273497000
C	-0.732863000	5.851212000	-1.077981000
H	0.263215000	5.818366000	-1.546220000
H	-1.421568000	5.235183000	-1.678159000
H	-1.090241000	6.884162000	-1.077033000
S	0.640255000	2.082990000	2.136811000
S	2.634804000	2.272525000	1.771943000
C	3.519034000	1.633028000	4.321860000
H	2.541140000	1.139111000	4.414670000
H	4.232651000	0.894331000	3.923747000
C	3.404986000	2.823972000	3.363136000
H	2.738772000	3.572384000	3.795738000
C	4.766182000	3.441595000	3.030943000
H	5.352678000	3.537139000	3.946836000
H	5.337300000	2.809720000	2.344256000
Te	4.763178000	5.431784000	2.141435000
C	4.822474000	6.509839000	4.018618000
H	3.927876000	6.299679000	4.603418000
H	5.725355000	6.244946000	4.569085000
H	4.849991000	7.569419000	3.756613000
O	3.951995000	2.098620000	5.598743000
C	4.142410000	1.040958000	6.527610000
H	3.208511000	0.488340000	6.711373000
H	4.908534000	0.331906000	6.179340000
H	4.475900000	1.493447000	7.464706000
O	0.232555000	4.768276000	5.103865000

H	1.445862000	4.569351000	6.406273000
O	-1.953686000	3.183880000	8.400423000
H	-2.308071000	2.433829000	8.891690000
H	-1.061707000	3.314980000	8.781099000
O	0.752699000	3.571329000	9.388566000
H	0.857933000	4.273889000	10.040612000
H	1.274858000	3.879204000	8.611148000
O	2.129026000	4.434340000	7.106444000
H	2.735219000	3.789180000	6.719637000

Te-4-PC(SAPE)

-6.81949519 Nimag=0

S	2.183278000	1.429837000	1.745763000
C	0.665454000	-0.807483000	1.313585000
H	1.601686000	-1.302988000	1.024824000
H	0.564287000	-0.895019000	2.408175000
C	0.727151000	0.663411000	0.899873000
H	0.933688000	0.721008000	-0.171048000
C	-0.518352000	1.476581000	1.230169000
H	-0.780335000	1.434459000	2.290675000
H	-0.392601000	2.523301000	0.946292000
Te	-2.374027000	0.992607000	0.132237000
C	-3.401641000	0.167610000	1.851946000
H	-3.443530000	0.961932000	2.598794000
H	-4.409694000	-0.099059000	1.529804000
H	-2.870386000	-0.709369000	2.222087000
O	-0.441331000	-1.433500000	0.667149000
C	-0.525566000	-2.823515000	0.934883000
H	-1.397259000	-3.203407000	0.395709000
H	0.370644000	-3.356003000	0.583259000
H	-0.652379000	-3.026125000	2.009917000
C	3.274473000	1.895422000	0.340327000
H	2.705871000	2.501930000	-0.369464000
H	4.007383000	2.549893000	0.827971000
C	3.956376000	0.744623000	-0.325579000
H	4.617915000	0.156521000	0.310906000
C	3.805312000	0.410105000	-1.609528000
H	3.147372000	0.964974000	-2.273544000
H	4.341695000	-0.434268000	-2.034922000
O	-3.158865000	2.661548000	0.145870000
H	-3.115556000	3.908860000	-1.060113000
O	0.213942000	1.767344000	-2.780863000
H	0.090960000	1.136345000	-3.499624000
H	-0.229190000	2.577919000	-3.106664000
O	-1.167387000	4.127816000	-3.687186000
H	-0.638614000	4.931915000	-3.744858000
H	-1.864018000	4.337551000	-3.022507000
O	-3.089574000	4.641731000	-1.728584000
H	-4.005682000	4.747894000	-2.008233000

S-1-TS(SAPE)

-6.00309807 Nimag=1 ν=-239.5

C	-3.318200000	-0.143700000	4.706800000
H	-3.120900000	-0.354800000	3.651900000
H	-3.866800000	-0.987300000	5.136100000
C	-2.014100000	0.089400000	5.478500000
H	-2.274500000	0.310900000	6.519000000
H	-3.965800000	0.732800000	4.775600000
C	-1.164200000	-1.188800000	5.437700000
H	-1.737200000	-2.039000000	5.815400000
H	-0.853500000	-1.424500000	4.414500000
S	0.407600000	-1.177900000	6.351100000
C	-0.106200000	-1.144500000	8.071500000
H	-0.600200000	-0.199400000	8.301500000
H	-0.754000000	-1.999500000	8.274800000
H	0.810100000	-1.229300000	8.658700000
N	-1.238600000	1.213600000	4.908200000
H	-0.234100000	1.083000000	4.979500000
S	-1.530100000	2.799400000	5.392500000
O	-2.510000000	2.808500000	6.460800000
O	-0.227100000	3.411300000	5.557100000
C	-2.289700000	3.543400000	3.952900000
H	-3.229300000	3.037500000	3.732600000
H	-2.472200000	4.588200000	4.212800000
H	-1.596600000	3.471600000	3.114600000
O	0.700500000	-3.113500000	6.386200000
H	1.145200000	-3.197400000	5.516800000
O	1.658300000	-3.030800000	3.502400000
H	2.544700000	-2.756700000	3.239300000
H	1.701900000	-4.020300000	3.499100000
O	1.764500000	-5.739000000	3.954800000
H	1.122200000	-6.337700000	3.557400000
H	1.430100000	-5.582900000	4.900700000
O	0.857400000	-5.124800000	6.347800000
H	1.553900000	-5.151000000	7.018800000

S-2-TS(SAPE)

-4.82817884 Nimag=1 ν=-225.2

C	-2.451400000	-1.058500000	3.240200000
H	-1.873500000	-1.156100000	2.315700000
H	-2.843900000	-2.041700000	3.516900000
C	-1.595600000	-0.460200000	4.364600000
H	-2.222300000	-0.434600000	5.271400000
H	-3.307700000	-0.407900000	3.033200000
C	-0.398800000	-1.399300000	4.630300000
H	-0.703700000	-2.446400000	4.532300000
H	0.383800000	-1.205200000	3.890200000
S	0.485000000	-1.343800000	6.221800000
C	-0.759600000	-1.893400000	7.404100000

H	-1.605900000	-1.205600000	7.423600000
H	-1.086100000	-2.908100000	7.159500000
H	-0.267400000	-1.886700000	8.378300000
N	-1.123000000	0.863100000	3.960900000
H	-0.713000000	1.349900000	4.753200000
H	-1.914800000	1.424100000	3.660300000
O	0.440100000	0.730000000	6.539100000
H	0.908300000	0.668800000	7.396300000
O	1.568700000	0.319000000	9.400800000
H	2.509600000	0.269700000	9.606500000
H	1.331000000	1.257400000	9.582800000
O	0.853400000	3.021200000	9.420700000
H	0.049700000	3.331400000	9.853000000
H	0.616600000	2.952300000	8.449400000
O	0.292600000	2.614700000	6.818800000
H	1.071700000	2.828100000	6.285000000

S-2-H$^+$-TS(SAPE)

-4.76404431 Nimag=1 ν=-201.7

C	-0.651935000	-2.095581000	2.560281000
H	-0.914358000	-1.238446000	1.934553000
H	0.368065000	-2.402058000	2.317033000
C	-0.749972000	-1.762880000	4.047828000
H	-0.439416000	-2.636828000	4.630751000
H	-1.312420000	-2.929675000	2.304057000
C	0.125098000	-0.554983000	4.427925000
H	1.063203000	-0.609341000	3.869336000
H	-0.366244000	0.390153000	4.180904000
S	0.596546000	-0.523445000	6.191336000
C	1.165474000	1.171130000	6.390321000
H	2.030116000	1.342381000	5.743676000
H	0.355239000	1.867324000	6.165353000
H	1.467797000	1.268775000	7.435217000
N	-2.187270000	-1.498018000	4.426763000
H	-2.239061000	-1.170627000	5.410970000
H	-2.747166000	-2.345670000	4.336053000
H	-2.634247000	-0.738010000	3.823495000
O	-1.374922000	-0.277242000	6.785493000
H	-1.374106000	-0.888716000	7.541379000
O	-3.322434000	-0.180903000	7.074453000
H	-3.325735000	0.166043000	7.980090000
H	-3.663077000	1.232347000	6.074316000
O	-3.728395000	1.931506000	5.370237000
H	-4.508983000	2.457222000	5.580479000
H	-3.537073000	1.196019000	3.853969000
O	-3.224950000	0.613556000	3.105574000
H	-3.918116000	0.630810000	2.434793000

S-3-TS(SAPE)

-8.71996937 Nimag=1 ν=-235.5

C	-2.825500000	4.099900000	1.799600000
H	-2.152600000	4.251800000	0.944000000
H	-3.633200000	3.421500000	1.476600000
C	-2.025700000	3.466400000	2.941200000
H	-1.246400000	4.161700000	3.259400000
C	-2.920000000	3.139300000	4.138600000
H	-3.444400000	4.050200000	4.438200000
H	-3.659300000	2.370100000	3.887500000
S	-1.993700000	2.547400000	5.587200000
C	-3.294300000	2.575700000	6.829900000
H	-3.663000000	3.595900000	6.954700000
H	-4.099700000	1.891400000	6.550700000
H	-2.833300000	2.239200000	7.760500000
O	-3.356000000	5.340500000	2.241100000
C	-4.007800000	6.059100000	1.206600000
H	-3.319800000	6.293300000	0.380000000
H	-4.866700000	5.501400000	0.800800000
H	-4.366300000	6.992900000	1.646600000
S	-1.191600000	1.913700000	2.362700000
S	0.378100000	2.530300000	1.224900000
C	2.307200000	1.523800000	2.963400000
H	1.471000000	0.899000000	3.306900000
H	2.842700000	0.966700000	2.178000000
C	1.773000000	2.843200000	2.401000000
H	1.377700000	3.447600000	3.219500000
C	2.848200000	3.637100000	1.635400000
H	3.793100000	3.551500000	2.179800000
H	3.013500000	3.194400000	0.646600000
S	2.520200000	5.400700000	1.331700000
C	3.094400000	6.157900000	2.877300000
H	2.513800000	5.843000000	3.745800000
H	4.156500000	5.957100000	3.044000000
H	2.958600000	7.234400000	2.744700000
O	3.179900000	1.810100000	4.053900000
C	3.802100000	0.645800000	4.577600000
H	3.062100000	-0.074700000	4.958100000
H	4.423400000	0.144700000	3.820000000
H	4.439200000	0.969400000	5.404200000
O	-1.221000000	4.359400000	6.100900000
H	-0.314000000	4.034100000	6.287100000
O	1.567600000	3.254700000	6.652400000
H	2.095600000	2.890900000	5.930100000
H	1.921400000	4.161600000	6.781100000
O	2.097300000	6.030500000	6.804700000
H	2.339300000	6.429200000	7.648200000
H	1.095300000	6.136100000	6.750100000
O	-0.556000000	6.100400000	6.676900000
H	-0.873100000	6.533400000	5.872300000

S-4-TS(SAPE)

-6.78404818 Nimag=1 ν=-221.1

S	-3.387400000	4.664300000	1.488700000
C	-2.309900000	2.722200000	3.040000000
H	-1.850600000	3.538600000	3.616100000
H	-3.331700000	2.575400000	3.432700000
C	-2.364200000	3.137500000	1.566800000
H	-1.360100000	3.412600000	1.236300000
C	-2.955700000	2.090600000	0.610200000
H	-3.849900000	1.627700000	1.040600000
H	-3.227600000	2.570600000	-0.331200000
S	-1.803800000	0.744800000	0.147900000
C	-2.771900000	-0.045400000	-1.149100000
H	-2.992100000	0.668300000	-1.945900000
H	-2.157700000	-0.856200000	-1.546200000
H	-3.694200000	-0.460200000	-0.733500000
O	-1.556800000	1.529000000	3.173400000
C	-1.414200000	1.114600000	4.520200000
H	-0.819500000	0.197800000	4.509400000
H	-0.892200000	1.872200000	5.125300000
H	-2.389100000	0.904500000	4.988500000
C	-2.468400000	5.697400000	0.273500000
H	-2.320900000	5.133100000	-0.650700000
H	-3.189200000	6.498100000	0.067300000
C	-1.184300000	6.256500000	0.794900000
H	-1.270900000	6.909400000	1.663600000
C	0.017300000	6.001600000	0.271100000
H	0.143100000	5.348400000	-0.588300000
H	0.914000000	6.448100000	0.693700000
O	-0.706800000	1.993100000	-1.190500000
H	0.165800000	1.710700000	-0.847800000
O	2.069100000	1.155900000	-0.077200000
H	2.432200000	0.271700000	-0.203300000
H	2.519800000	1.703800000	-0.755700000
O	2.862800000	2.834600000	-2.209400000
H	3.245300000	3.696400000	-2.008700000
H	1.886100000	3.007900000	-2.327900000
O	0.178100000	3.175200000	-2.359200000
H	-0.166300000	2.733600000	-3.147600000

Se-1-TS(SAPE)

-5.97545994 Nimag=1 ν=-151.7

C	-3.906000000	-0.111100000	4.577000000
H	-3.703500000	-0.297100000	3.517500000
H	-4.373200000	-1.007300000	4.996300000
C	-2.615600000	0.238800000	5.339800000
H	-2.874300000	0.425900000	6.384300000
H	-4.622800000	0.711100000	4.647000000
C	-1.642200000	-0.946600000	5.269100000
H	-2.114700000	-1.862200000	5.630400000
H	-1.312500000	-1.132500000	4.241800000
Se	0.059000000	-0.819600000	6.255100000
C	-0.584800000	-0.973100000	8.086700000
H	-1.141100000	-0.075500000	8.353400000
H	-1.184400000	-1.879600000	8.170600000
H	0.309500000	-1.058900000	8.705900000
N	-2.025100000	1.483300000	4.833900000
H	-1.369200000	1.412600000	4.066600000
S	-1.721600000	2.832300000	5.748600000
O	-1.849600000	2.489200000	7.151700000
O	-0.514400000	3.423800000	5.212000000
C	-3.072300000	3.947100000	5.365300000
H	-4.014800000	3.488800000	5.667400000
H	-2.895100000	4.859000000	5.939200000
H	-3.062400000	4.160600000	4.296100000
O	0.390500000	-2.988700000	6.221200000
H	0.743400000	-2.997700000	5.313100000
O	0.976600000	-2.737600000	3.023400000
H	1.814000000	-2.500700000	2.608000000
H	1.008600000	-3.720600000	3.077500000
O	1.110800000	-5.476900000	3.610200000
H	0.431800000	-6.087100000	3.301100000
H	0.909600000	-5.339700000	4.584100000
O	0.523000000	-4.899700000	6.163400000
H	1.293600000	-4.923100000	6.748200000

Se-2-TS(SAPE)

-4.79990238 Nimag=1 ν=-165.4

C	-2.089100000	-0.832900000	2.450800000
H	-1.416300000	-0.843000000	1.587500000
H	-2.464200000	-1.846900000	2.618400000
C	-1.377700000	-0.285500000	3.696800000
H	-2.099100000	-0.336500000	4.530000000
H	-2.947700000	-0.200400000	2.200800000
C	-0.190500000	-1.203000000	4.030700000
H	-0.468800000	-2.256100000	3.935600000
H	0.642600000	-0.998900000	3.351900000
Se	0.647000000	-1.115100000	5.821800000
C	-0.812800000	-1.832900000	6.907300000
H	-1.685000000	-1.185900000	6.820000000
H	-1.039000000	-2.851800000	6.587800000
H	-0.448100000	-1.824800000	7.935000000
N	-0.910800000	1.074400000	3.429500000
H	-0.613300000	1.519800000	4.292700000
H	-1.683400000	1.631200000	3.075800000
O	0.346200000	1.090700000	6.235200000
H	0.615700000	0.985600000	7.168400000
O	0.946500000	0.557700000	9.308000000

H	1.832400000	0.544200000	9.689000000
H	0.636300000	1.478600000	9.458400000
O	0.111800000	3.245400000	9.246400000
H	-0.765100000	3.512900000	9.544300000
H	0.044200000	3.201700000	8.251800000
O	0.008100000	2.903100000	6.553500000
H	0.837300000	3.195800000	6.148100000

Se-2-H^{+}-TS(SAPE)

-4.73725564 Nimag=1 ν=-127.4

C	-0.691700000	-2.171800000	2.498500000
H	-0.936700000	-1.299500000	1.886600000
H	0.304500000	-2.521300000	2.217600000
C	-0.723000000	-1.843900000	3.990900000
H	-0.439900000	-2.739300000	4.555600000
H	-1.397700000	-2.974000000	2.261800000
C	0.218400000	-0.686400000	4.346100000
H	1.167800000	-0.820200000	3.823900000
H	-0.201400000	0.281600000	4.064300000
Se	0.701800000	-0.589900000	6.269600000
C	1.049100000	1.324700000	6.399500000
H	1.862700000	1.582300000	5.719700000
H	0.132200000	1.866000000	6.166000000
H	1.350300000	1.502300000	7.433100000
N	-2.128400000	-1.506500000	4.423200000
H	-2.116800000	-1.175300000	5.414300000
H	-2.736800000	-2.322300000	4.357900000
H	-2.557000000	-0.727500000	3.836900000
O	-1.445500000	-0.386400000	6.857100000
H	-1.455200000	-1.050700000	7.566900000
O	-3.359100000	-0.236100000	7.161000000
H	-3.315700000	0.147400000	8.051400000
H	-3.667300000	1.191300000	6.143500000
O	-3.706100000	1.905300000	5.454900000
H	-4.485800000	2.435100000	5.658300000
H	-3.475000000	1.214400000	3.910300000
O	-3.148600000	0.657600000	3.151000000
H	-3.826000000	0.703300000	2.465600000

Se-3-TS(SAPE)

-8.65296200 Nimag=1 ν=-189.3

C	-0.221400000	4.165700000	0.978200000
H	0.792700000	4.436000000	0.656200000
H	-0.669700000	3.533600000	0.193400000
C	-0.134900000	3.392000000	2.293600000
H	0.306200000	4.026200000	3.062600000
C	-1.501200000	2.841200000	2.743500000
H	-2.301100000	3.220500000	2.104600000
H	-1.538800000	1.748000000	2.703800000
Se	-2.115300000	3.225600000	4.581400000
C	-2.597600000	5.109600000	4.394400000
H	-1.735000000	5.667900000	4.035400000
H	-3.440800000	5.201400000	3.708300000
H	-2.876200000	5.434700000	5.397400000
O	-1.002900000	5.337100000	1.182200000
C	-1.058400000	6.163000000	0.030900000
H	-0.057800000	6.510200000	-0.268200000
H	-1.517800000	5.641500000	-0.823900000
H	-1.672400000	7.029700000	0.289100000
S	0.969200000	1.907400000	2.101300000
S	2.840200000	2.595100000	1.712500000
C	4.093700000	1.490100000	3.920200000
H	3.235600000	0.801800000	3.914900000
H	4.881200000	1.047700000	3.287300000
C	3.659600000	2.846600000	3.353500000
H	2.923900000	3.295700000	4.022200000
C	4.842500000	3.799800000	3.146100000
H	5.502300000	3.741600000	4.014000000
H	5.433100000	3.516600000	2.268800000
Se	4.413900000	5.708500000	2.880700000
C	4.192700000	6.225200000	4.764300000
H	3.377100000	5.669700000	5.226500000
H	5.127400000	6.075100000	5.307700000
H	3.950100000	7.290300000	4.746800000
O	4.564600000	1.673300000	5.249600000
C	4.992500000	0.464700000	5.850600000
H	4.176500000	-0.273100000	5.911400000
H	5.832100000	0.009400000	5.301200000
H	5.323100000	0.711300000	6.862900000
O	-0.118000000	3.574100000	5.563000000
H	-0.563800000	3.769600000	6.408300000
O	-1.814300000	4.529700000	8.132800000
H	-2.209200000	3.952900000	8.797200000
H	-0.944600000	4.778000000	8.518000000
O	0.867700000	4.986200000	8.871200000
H	1.217100000	5.883300000	8.918300000
H	1.178700000	4.634500000	7.990000000
O	1.498600000	4.001200000	6.422300000
H	1.790000000	3.080400000	6.486600000

Se-4-TS(SAPE)

-6.75629800 Nimag=1 ν=-163.0

S	2.777800000	2.879800000	3.044900000
C	2.067000000	0.308600000	2.510000000
H	3.137100000	0.155800000	2.308800000
H	1.908000000	0.162100000	3.592700000
C	1.685900000	1.733600000	2.102000000
H	1.923200000	1.870400000	1.044600000
C	0.230400000	2.124200000	2.362800000

H	-0.069800000	1.890500000	3.387800000
H	0.094400000	3.190700000	2.185000000
Se	-1.099800000	1.265100000	1.158600000
C	-2.563200000	2.509500000	1.527400000
H	-2.262000000	3.516700000	1.238300000
H	-3.406900000	2.184100000	0.916000000
H	-2.834000000	2.464700000	2.583800000
O	1.283600000	-0.611300000	1.767300000
C	1.568200000	-1.963700000	2.076700000
H	0.921100000	-2.577400000	1.445300000
H	2.618700000	-2.215900000	1.863800000
H	1.360400000	-2.192600000	3.134200000
C	3.232600000	4.125700000	1.769000000
H	2.324800000	4.527200000	1.311300000
H	3.681500000	4.921600000	2.375600000
C	4.199200000	3.626300000	0.744600000
H	5.171000000	3.313200000	1.126500000
C	3.940700000	3.541000000	-0.562800000
H	2.980400000	3.829300000	-0.982600000
H	4.694400000	3.173900000	-1.255100000
O	-0.340500000	2.604100000	-0.611600000
H	-0.359700000	1.849800000	-1.232400000
O	-0.346100000	0.188300000	-2.710100000
H	-1.151700000	-0.164700000	-3.105000000
H	0.001100000	0.808400000	-3.383700000
O	0.607100000	2.382900000	-4.279900000
H	1.542800000	2.420000000	-4.509300000
H	0.526300000	2.925700000	-3.453900000
O	0.290600000	3.716400000	-1.884300000
H	-0.518400000	4.246600000	-1.911500000

Te-1-TS(SAPE)

-5.95067977 Nimag=1 ν=-163.6

C	-3.277300000	-1.772600000	3.526100000
H	-2.677100000	-2.290300000	2.769500000
H	-3.770400000	-2.532800000	4.139600000
C	-2.403400000	-0.853900000	4.397400000
H	-3.054400000	-0.354800000	5.118300000
H	-4.049800000	-1.190700000	3.018300000
C	-1.340300000	-1.670300000	5.143500000
H	-1.785400000	-2.534900000	5.640500000
H	-0.569000000	-2.044600000	4.464600000
Te	-0.231500000	-0.649200000	6.706600000
C	-1.645200000	-1.029400000	8.283500000
H	-2.612700000	-0.596100000	8.030600000
H	-1.706200000	-2.107600000	8.427900000
H	-1.249900000	-0.552100000	9.181700000
N	-1.759300000	0.216300000	3.613100000
H	-1.346000000	-0.107600000	2.744100000
S	-2.528500000	1.671800000	3.299200000
O	-2.378800000	1.925300000	1.879700000
O	-3.829200000	1.670800000	3.938500000
C	-1.514100000	2.870000000	4.160500000
H	-0.506600000	2.844300000	3.744400000
H	-1.976200000	3.843600000	3.985100000
H	-1.504400000	2.633900000	5.224300000
O	0.798200000	-2.841900000	7.563500000
H	1.439900000	-2.855200000	6.827500000
O	2.676600000	-3.161000000	4.955300000
H	3.504800000	-2.695800000	4.788700000
H	2.957600000	-4.018900000	5.334300000
O	3.237500000	-5.496100000	6.525000000
H	2.941800000	-6.374400000	6.259100000
H	2.557600000	-5.184800000	7.168800000
O	1.287500000	-4.381200000	8.191700000
H	1.672800000	-4.015500000	9.001600000

Te-2-TS(SAPE)

-4.77624673 Nimag=1 ν=-146.2

C	-0.950000000	-3.346500000	8.751300000
H	0.102300000	-3.617100000	8.890400000
H	-1.357300000	-3.052900000	9.725600000
C	-1.099700000	-2.216200000	7.716400000
H	-2.170400000	-1.994200000	7.615300000
H	-1.490000000	-4.240800000	8.421800000
C	-0.401600000	-0.942000000	8.189300000
H	-0.919300000	-0.489200000	9.037200000
H	0.641600000	-1.125800000	8.457600000
Te	-0.340500000	0.513400000	6.579800000
C	0.134900000	2.173900000	7.886200000
H	-0.669600000	2.329700000	8.606300000
H	1.078900000	1.970200000	8.392500000
H	0.239100000	3.058700000	7.255200000
N	-0.628100000	-2.540300000	6.365500000
H	-1.105800000	-3.358800000	6.000100000
H	0.368300000	-2.746300000	6.368000000
O	2.239000000	-0.296400000	6.450500000
H	2.281500000	-0.011000000	5.516600000
O	2.625500000	0.442400000	3.344100000
H	2.791300000	1.330800000	3.007900000
H	3.505800000	0.020800000	3.361900000
O	5.108200000	-0.907400000	4.012400000
H	5.237400000	-1.819700000	3.727500000
H	4.716500000	-0.979200000	4.910900000
O	3.790600000	-0.923200000	6.527400000
H	4.148000000	-0.227800000	7.098700000

Te-2-H$^+$-TS(SAPE)

-4.71318934 Nimag=1 ν=-141.5

C	-0.720500000	-0.004200000	2.142800000
H	-0.332400000	-0.930500000	1.710000000
H	-1.807000000	-0.005600000	2.029400000
C	-0.359200000	0.125500000	3.622700000
H	-0.781800000	1.059800000	4.007900000
H	-0.333600000	0.842000000	1.565500000
C	-0.865200000	-1.053600000	4.454800000
H	-1.923200000	-1.206400000	4.236500000
H	-0.332400000	-1.977100000	4.216900000
Te	-0.761100000	-0.818200000	6.633400000
C	0.189200000	-2.704200000	7.045800000
H	-0.461900000	-3.508400000	6.704000000
H	1.163500000	-2.732200000	6.560500000
H	0.303100000	-2.751600000	8.130000000
N	1.136500000	0.265900000	3.776800000
H	1.464000000	1.122700000	3.330300000
H	1.666400000	-0.538300000	3.344500000
H	1.402500000	0.307100000	4.791000000
O	4.476000000	-1.647100000	4.958900000
H	4.270100000	-0.840400000	5.470400000
H	5.438500000	-1.708300000	4.933500000
O	2.639600000	-1.874700000	2.930700000
H	2.986200000	-2.112700000	2.062300000
H	3.408500000	-1.887900000	3.549100000
O	1.775700000	0.171100000	6.518000000
O	3.412200000	0.677700000	6.410300000
H	3.658200000	0.465600000	7.326200000
H	1.462900000	0.987900000	6.943200000

Te-3-TS(SAPE)

-8.60086348 Nimag=1 ν=-168.4

C	-3.588500000	4.275400000	1.744200000
H	-2.952300000	4.357200000	0.851200000
H	-4.474500000	3.677000000	1.473800000
C	-2.799700000	3.577700000	2.857100000
H	-1.942500000	4.195700000	3.131300000
C	-3.653700000	3.312400000	4.092500000
H	-4.029800000	4.262600000	4.475200000
H	-4.501800000	2.660500000	3.867700000
Te	-2.571400000	2.414500000	5.758800000
C	-3.784800000	3.350300000	7.272100000
H	-3.620700000	4.426300000	7.221200000
H	-4.832800000	3.089000000	7.123400000
H	-3.438200000	2.964900000	8.232400000
O	-3.972400000	5.566600000	2.194800000
C	-4.627700000	6.323400000	1.192000000
H	-3.982300000	6.481200000	0.313900000
H	-5.559500000	5.839700000	0.858100000
H	-4.870200000	7.293700000	1.633000000
S	-2.137700000	1.955200000	2.250600000
S	-0.608500000	2.406200000	0.981800000
C	1.399900000	1.201800000	2.471500000
H	0.568800000	0.635900000	2.916100000
H	1.764700000	0.636700000	1.599400000
C	0.905900000	2.584100000	2.032700000
H	0.619700000	3.163600000	2.912600000
C	1.945800000	3.350100000	1.214000000
H	2.907900000	3.313600000	1.728700000
H	2.080800000	2.905600000	0.224500000
Te	1.551300000	5.477400000	0.912800000
C	3.186600000	6.177600000	2.149800000
H	3.041000000	5.866200000	3.183400000
H	4.133200000	5.811300000	1.752300000
H	3.163700000	7.267600000	2.089400000
O	2.445200000	1.363800000	3.428600000
C	3.015700000	0.125200000	3.827300000
H	2.270200000	-0.532000000	4.299900000
H	3.461900000	-0.405500000	2.972900000
H	3.798900000	0.353300000	4.554200000
O	-1.104400000	4.545600000	5.968500000
H	-0.288900000	4.005800000	6.017300000
O	1.586400000	2.934600000	6.168400000
H	1.935800000	2.543600000	5.356500000
H	2.091200000	3.760400000	6.284200000
O	2.483400000	5.715900000	6.411000000
H	2.758400000	6.056300000	7.270300000
H	1.517000000	5.909300000	6.365000000
O	-0.295300000	6.044900000	6.212400000
H	-0.534300000	6.406600000	5.346400000

Te-4-TS(SAPE)

-6.73071491 Nimag=1 ν=-150.2

S	2.307400000	0.923000000	2.188100000
C	1.019000000	-1.237200000	1.148200000
H	1.987300000	-1.455400000	0.675800000
H	1.022500000	-1.701500000	2.149300000
C	0.845100000	0.279000000	1.262800000
H	0.897600000	0.708300000	0.259700000
C	-0.430800000	0.732500000	1.966400000
H	-0.519200000	0.284100000	2.958500000
H	-0.439200000	1.817900000	2.067000000
Te	-2.305700000	0.321700000	0.906200000
C	-3.383300000	1.897400000	1.918700000
H	-2.920900000	2.854900000	1.678500000
H	-4.407700000	1.875600000	1.541800000
H	-3.385300000	1.717600000	2.994400000
O	-0.042700000	-1.763400000	0.365000000
C	-0.006600000	-3.175100000	0.249800000

H	-0.853000000	-3.465700000	-0.377200000
H	0.924900000	-3.517800000	-0.226100000
H	-0.101300000	-3.665900000	1.231200000
C	2.737700000	2.438900000	1.240100000
H	1.854800000	3.077600000	1.151800000
H	3.444500000	2.939300000	1.913100000
C	3.368500000	2.171500000	-0.088000000
H	4.312900000	1.627900000	-0.059400000
C	2.853100000	2.541200000	-1.263000000
H	1.907700000	3.072400000	-1.338100000
H	3.370000000	2.317700000	-2.192900000
O	-1.509900000	2.275300000	-0.818700000
H	-1.762600000	1.697900000	-1.566000000
O	-2.193500000	0.579000000	-3.468500000
H	-3.092400000	0.452000000	-3.793700000
H	-1.834300000	1.298000000	-4.021900000
O	-1.021200000	3.015900000	-4.545200000
H	-0.116500000	2.994000000	-4.878400000
H	-0.934500000	3.271000000	-3.601000000
O	-0.958200000	3.553000000	-1.743900000
H	-1.678700000	4.157300000	-1.512900000
